# Transcranial photobiomodulation therapy with 808 nm light changes expression of genes and proteins associated with neuroprotection, neuroinflammation, oxidative stress, and Alzheimer’s disease: Whole RNA sequencing of mouse cortex and hippocampus

**DOI:** 10.1371/journal.pone.0326881

**Published:** 2025-07-18

**Authors:** Binjun Li, Iuliia Golovynska, Yurii V. Stepanov, Sergii Golovynskyi, Andrii Golovynskyi, Denis Kolesnik, Liudmyla I. Stepanova, Puxiang Lai, Fangrui Lin, Junle Qu

**Affiliations:** 1 College of Physics and Optoelectronic Engineering, Center for Biomedical Photonics, Key Laboratory of Optoelectronic Devices and Systems of Ministry of Education and Guangdong Province, Shenzhen University, Shenzhen, P. R. China; 2 R.E. Kavetsky Institute of Experimental Pathology, Oncology and Radiobiology, NAS of Ukraine, Kyiv, Ukraine; 3 V.M. Glushkov Institute of Cybernetics, NAS of Ukraine, Kyiv, Ukraine; 4 Institute of Biology and Medicine, Taras Shevchenko National University of Kyiv, Kyiv, Ukraine; 5 Department of Biomedical Engineering, The Hong Kong Polytechnic University, Hong Kong SAR, China; Massachusetts General Hospital, UNITED STATES OF AMERICA

## Abstract

Light therapy, using red and near-infrared (NIR) irradiation, is currently applied for the treatment of various neurodegenerative diseases, such as Alzheimer’s disease (AD). Transcranial photobiomodulation therapy (tPBMT) can alleviate neurodegeneration, neuronal loss, and β-amyloid peptide plaque burden. Alternatively, potential early inhibition of oxidative stress, neuroinflammation, apoptosis, and amyloidogenic cellular pathways may constrain pathological changes with aging. In this research, we conduct an 808-nm tPBMT with a 30-day course of daily 1-hour sessions for mice and assess its influence on molecular mechanisms related to the potential onset of neurodegeneration. To comprehensively identify molecular mechanisms of tPBMT on the brain cells, the next-generation whole RNA sequencing of over 30,000 mRNA of the cortex and hippocampus of BALB/c mice is performed. After tPBMT, transcriptional alterations are found in 1,005 genes in the hippocampus and 1,482 genes in the cortex. Pathway-gene enrichment network analysis identifies genes associated with about 20 pathways of neurodegeneration, and a disease-gene network is constructed. Particularly, tPBMT alters the transcription and expression of the essential genes associated with oxidative stress (NF-κBIα, JUN, JUND, and PKC genes), inflammation (DOCK4/6, IL-1RAPL1, and TNFαIP6), and apoptosis (CASP3, TNFαIP6, AKT3, CDKN1A, CYP51, RASA2, and RESTAT). Additionally, 808-nm light modulates the main risk genes for AD (BACE1, BACE2, PSEN2, APH1B, GATA2, YY2, RELA, STAT3, JUN, JUND, ARNTL, CREB3L1, CELF2, E2F4, ELK3, and CEBPD), involved in APP processing supporting AD development. Moreover, the APP concentration is reduced after tPBMT. Hence, PBMT may help inhibit the development of different neurodegeneration types and maintain normal brain conditions.

## Introduction

Low-intensity light therapy, also called photobiomodulation (PBM) and utilizing 600–980 nm red and near-infrared (NIR) irradiation, is a promising noninvasive approach now actively used in clinics and widely researched. Transcranial PBM therapy (tPBMT) has been applied for the treatment of various neurodegenerative diseases, such as Alzheimer’s disease (AD), Parkinson’s disease (PD), and amyotrophic lateral sclerosis (ALS) [1–6]. The depositions of misfolded β-amyloid (Aβ), tau, and α-synuclein are the main drivers and earliest signatures in these disorders. Among them, AD is the most common cause of dementia, characterized by a progressive decline in memory, thinking, and behavior. The characteristic hallmark of AD is senile plaques containing toxic oligomers р40/42/43 of Aβ. Several studies show that red/NIR-PBM can considerably attenuate Aβ burden and plaque development [2–11], improve spatial memory [10,12], preserve motor and cognitive skills [13], reduce neuronal loss and microgliosis in transgenic animal models [13,14], as well as decelerate neurodegenerative disease progression. Notable that it is difficult to perform even such moderate improvements through pharmacological interventions.

The already explored pathways of initial red/NIR light-to-cell interaction are limited with the relation to (i) mitochondrial cytochrome-c-oxidase (CCO) photoacceptor absorbing light within 600–980 nm [15,16] and (ii) calcium (Ca) ion channels activated due to the light-induced depolarization of the cellular membrane [17–19]. Photoactivated CCO causes changes in the mitochondrial ultrastructure, resulting in an increase of the mitochondrial membrane potential [20] and triggering photochemical reactions by several intracellular pathways mediated through reactive oxygen species (ROS) [20], adenosine triphosphate [21], and caspase family members [22,23]. A light-increased cytosolic Ca^2+^ concentration can activate NO synthesis [24], as well as the phosphoinositide-3-kinase/serine-threonine kinase (PI3K/AKT) and mitogen-activated protein kinase (MAPK) signaling pathways [25]. Through such events, red/NIR light can significantly reduce neuroinflammation and oxidative stress as well as restore mitochondrial homeostasis [5,19,26–35], in turn, enhancing cell proliferation and survival along with suppressing neuronal apoptosis and aging [6,9,11,19,34–38].

Despite extensive scientific research in the field of neuro-PBM (or photoneuromodulation), the study of cellular and molecular mechanisms by which light protects against neurodegeneration is still in its earliest stages. During AD pathogenesis, Aβ is produced through the increased expression of transmembrane β-secretase β-site amyloid precursor protein (APP) cleaving enzyme1 (BACE, β-secretase) and γ-secretase, which affect APP biogenesis. The γ-secretase consists of presenilin (PSEN), presenilin enhancer 2 (PEN2), nicastrin (NCT), and anterior pharynx-defective 1 (APH1) [39,40]. Investigating the Aβ-related pathways in pathological AD mouse models, our recent study [6] shows that NIR-tPBMT can downregulate Ca^2+^/calmodulin-dependent BACE1/2, PSEN1/2, and APP and normalize cholesterol homeostasis via the HMGCR, DHCR7, and INSIG1 genes in the brain cortex and hippocampus. This confirms the report that NIR-tPBMT can reduce the APP and BACE gene levels via the activation of the cyclic adenosine monophosphate/protein-kinase-A (cAMP/PKA) and PKA/sirtuin-1 signaling pathways [5]. Also, it can suppress c-Jun N-terminal kinase 3 (JNK3) via the MAPK signaling [25].

Therefore, systematically applied red/NIR-PBMT may be promising in combating neurodegenerative diseases, partially restoring synaptic plasticity in pathologically degenerating neurons, reducing existing Aβ load, and slightly improving a patient's condition. Nonetheless, toxic Aβ also accumulates in the brains of healthy people, and this may transform in some conditions into a pathological neurodegeneration [41,42]. A potential early inhibition of oxidative stress, neuroinflammation, apoptosis, and amyloidogenic cellular pathways may constrain pathological changes with aging. As a rule, this also relates to other diseases. Yet, an early prevention of neurodegeneration and other pathologies using red/NIR-PBMT has not been comprehensively studied.

In this research, we conducted an 808-nm tPBMT with a 30-day course of daily 1-hour sessions for mice in vivo and assessed its influence on molecular mechanisms. Our therapy was piloted on BALB/c mice as a model of a normal healthy organism. Particularly, NIR-tPBMT was applied to modulate the expression of AD-related genes. This is important for assessing the light effect on the brain, exploring new molecular mechanisms of tPBMT against neurodegenerative diseases, and preventing their development using noninvasive therapy. To identify molecular mechanisms of light influence on brain cells in vivo, we performed the next-generation whole RNA sequencing (RNA-Seq) of the hippocampus and cortex of mice after the 808-nm NIR-tPBMT course. Transcriptional alterations of genes and pathway-gene enrichment network analysis are provided, particularly focusing on the dozens of known genes of neurodegenerative diseases.

## Materials and methods

### Animals and preparation

Healthy female BALB/c mice, 3 months old (18–20 g), were obtained from Guangdong Medical Laboratory Animal Center. The mice were kept in collective laboratory cages under a 12 h light/dark cycle and standard feeding. Both the NIR-tPBMT experimental and control groups contained 9 mice. The heads of the mice were shaved weekly. For each of the three types of measurements, three mice were randomly chosen from each group. The mice were sacrificed 1 h after NIR-tPBMT by cervical dislocation [43] to prevent chemical anesthesia from interfering with the aims of a research project, and the brains were extracted to prepare for assaying. For RNA-seq and Western blot (WB) analyses, the frontal cortex and hippocampus tissues were extracted and immediately frozen at – 80°C. For histological examination, the formalin-fixed paraffin-embedding method was used. The animal studies were carried out at Shenzhen University, China, in strict accordance with the recommendations about the general ethical principles of animal experiments in the “Guide for the Care and Use of Laboratory Animals” by the National Institutes of Health and the experimental protocols approved by the Shenzhen University Bioethics Committee for Animal Experiments.

### Near-infrared light treatment

**Fig 1:**
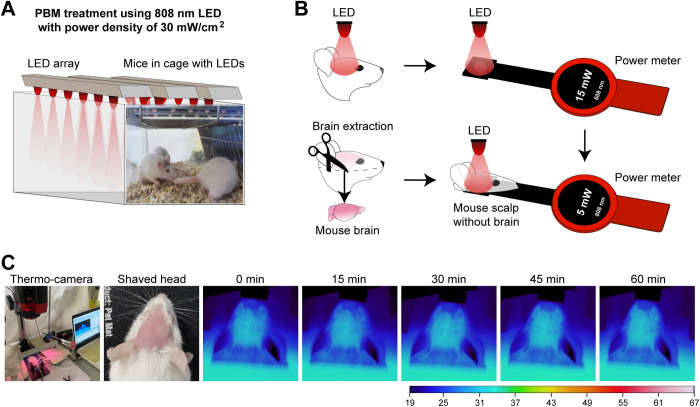
Transcranial light therapy (tPBMT). **(A)** The experimented mice with shaved heads were placed in a chamber illuminated by an 808-nm LED array with a power density of 30 mW/cm^2^; a schematic illustration of LED arrays showed the distribution of light in a chamber. **(B)** Schematically illustrated measurements of the power density of incident light at the top of the mouse skull and the light penetration through the skull tissues. **(C)** The monitoring of temperature on a shaved mouse head during PBM treatment. The thermal imaging camera shows that the temperature of the head skin does not rise above 34°C.

The experimented mice with shaved heads were placed in a chamber illuminated by a CW 808 nm NIR light-emitting diode (LED) array and exposed for 1 h (Fig 1A). The power density of incident light at the top of the mouse skull was measured to be 30 ± 3 mW/cm^2^, resulting in an incident light dose of 108 ± 11 J/cm^2^ (Fig 1B). The control group was also kept in a similar chamber for 1 h except for NIR-tPBMT. The light treatment was performed for 30 consecutive days before sample collection. The measurement of light penetration through the skull tissues showed the intensity of light reaching the mouse brain to be 10 ± 2 mW/cm^2^, resulting in an irradiation dose of 36 ± 7 J/cm^2^ during 1h. These light power density and dose were chosen because light irradiation with similar parameters was shown to provide different positive effects on cells and tissues while not causing cell phototoxicity [2–11,18,35]. The diffuse light was measured using a power meter with a detector area of 0.5 cm^2^, as schematically shown in Fig 1B. A NIR light wavelength of 808 nm (the NIR-I optical window) has been chosen for therapy due to its better penetration through biological tissue caused by lower absorption and scattering [44]. Using a thermal imaging camera, the monitoring of temperature changes on a mouse head was provided when designing the experiment (this anesthetized mouse was not from the two experimental groups). Fig 1C shows that the temperature of a mouse's head skin during 1 h does not rise above 34°C.

### Next-generation whole-transcriptome RNA sequencing

Total RNA was isolated from the tissue samples using an RNAmini kit (Qiagen, Germany). RNA quality was examined by gel electrophoresis and with Qubit (Thermo, Waltham, USA). All samples showed their quality within the normal range (Figs A and B in S3 File). The pie chart shows the distribution of read comparison for exons, introns, and gene interval regions of all samples in Fig C in S3 File. The RNA samples of the control and light-treated groups were separated into independent pools, and strand-specific libraries were constructed using a TruSeq RNA sample preparation kit (Illumina, USA). The sequencing was carried out using an Illumina Novaseq 6000 instrument. The raw data were handled by Skewer and checked by FastQC v0.11.2. Clean reads were aligned to the Mus musculus mouse genome using STAR (Fig C in S3 File). The expression of gene transcript was calculated by fragments per kilobase of exon model per million mapped reads (FPKM) using Perl. The data of sequencing were presented using the 2-fold units. DESeq2 software was used to screen differentially expressed genes between different sample groups to meet the requirements of |log2FC| ≥ 1 and *P* ≤ 0.05 (*N* = 3). Then, the enrichment analysis for molecular functions, biological processes, cellular components, and signaling pathways was performed using standard databases. All details on the used RNA sequencing method are in S3 File. The raw sequence data reported in this paper were deposited in the Genome Sequence Archive (GSA, Genomics, Proteomics & Bioinformatics 2021) in National Genomics Data Center (Nucleic Acids Res 2022), China National Center for Bioinformation/ Beijing Institute of Genomics, Chinese Academy of Sciences (https://ngdc.cncb.ac.cn/bioproject/browse/PRJCA022123).

### Western blot analysis

WB analysis to determine proteins in the mouse brain was performed according to the standard method described in S3 File. The samples were bound to the monoclonal antibodies (Thermo Fisher Scientific, USA, Table A in S3 File) after blocking the non-specific binding of the antibodies. The density of each band was normalized to β-actin. The original WB images are presented in S1 File and the raw data sets are given in S2 File.

### Immunohistochemistry

The formalin-fixed paraffin-embedding method was used for the histological examination of a whole mouse brain. Briefly, the brains were fixed in 4% PFA, then rinsed in PBS, dehydrated in increasing concentrations of ethanol (70º, 80º, 90º, 96º), cleared in xylene, soaked in paraffin wax, and embedded in boxes with paraffin (Thermo Fisher Scientific, USA). Serial sections (4 μm thickness) were sliced using a microtome and serially glued onto glass slides. The tissue slices were stained with Arginase 1 antibody, inducible nitric oxide synthase (iNOS) antibody [45], and DAPI fluorescent probe (Thermo Fisher Scientific, USA). Histological images were obtained by exploiting the Nikon fluorescent microscope and analyzed using ImageJ software. The fluorescence intensity was used to evaluate the staining, the raw data sets are given in S2 File.

### Statistical analysis

Control and sample measurements of WB and immunohistochemical analyses were subjected to statistical analysis using the Student’s *t*-test (*N* = 3) in OriginLab. The results were expressed as the mean ± standard deviation (M ± SD); significance was set at *P* < 0.05.

## Results

### Transcriptome profiling reveals cerebral regulatory mechanisms of NIR light

**Fig 2:**
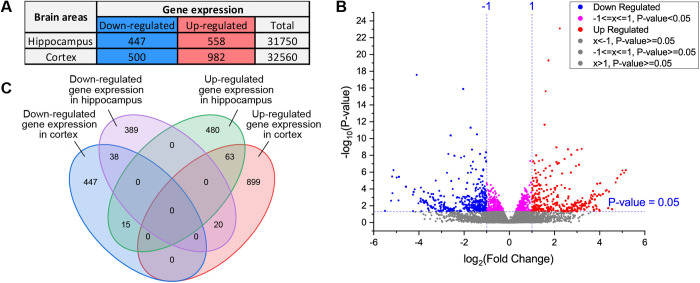
Whole-transcriptome analysis of two brain regions of BALB/c mice. **(A)** Statistically significant differentially expressed coding RNAs in the hippocampus and cortex of BALB/c mice after transcranial 808 nm light therapy. **(B)** Differential expression gene screening. Each point of the volcano chart represents a gene; the abscissa represents the value of log2FC, and │log2FC│ ≥ 1 is set as a significantly different gene, where the dots of ≥1/ ≤ –1 represent genes significantly upregulated/downregulated in the experimental group compared to the control group; the ordinate represents –log10 (*P*-value) and when *p* ≤ 0.05, so, significantly different genes. Therefore, the blue and purple dots on the left of 0 represent significantly downregulated genes, the red and purple dots on the right represent significantly upregulated genes, and the gray dots represent the non-significant difference genes. **(C)** Venn diagram for the indicated comparisons showing overlap among the up- and down-regulated genes identified by RNA-seq in different brain regions after light therapy.

**Fig 3:**
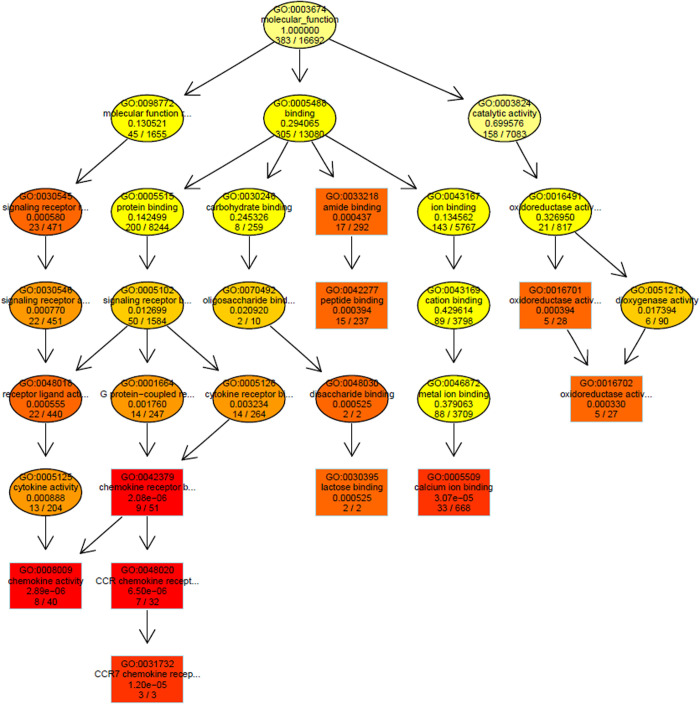
Significant enrichment tree chart of genes modulated by 808 nm light in the hippocampus. Each gene ontology (GO) category of molecular functions shows the top 10 GOs with significant enrichment (in the boxes). Each node represents a GO, with the color depth indicating the degree of enrichment. The darker the color, the higher the degree of enrichment, and each node displays a GO name and *P*-value. The tree charts for biological process, molecular function, and cellular component changes in the hippocampus and cortex are in Figs D-I in S3 File.

Whole-transcriptome analysis of the hippocampus and frontal cortex of BALB/c mice (Fig 2 and GSA, PRJCA022123) confirmed that tPBMT using 808 nm NIR light modulates the cellular mechanisms and revealed new changes in gene transcription. Genes with significantly altered expression following NIR-tPBMT were classified into ontologies and subjected to enrichment analysis, with ontologies being grouped into three categories: biological process (BP), molecular function (MF), and cellular component (CC). The DEG analysis resulted in the formation of respective significant enrichment tree charts, where each gene ontology (GO) category shows the top 10 GOs with significant enrichment and Reactome pathway analysis (Figs D-I in S3 File). The representative enrichment tree chart for BP changes in the hippocampus is presented in Fig 3.

Investigating molecular function, we attempted to identify potential transcription factors (TFs), signal transducers, and activators of transcription that regulate differentially expressed metabolic genes. We performed the enrichment analysis to identify differentially expressed metabolic genes in comparison to the control untreated mice and those after the tPBMT course. They are 23 TFs in the cortex (Fig E in S3 File) and 16 TFs in the hippocampus (Fig H in S3 File), which are mostly associated to activating/basic/general TFs and those related to GATA binding protein 2 (GATA2), yin yang 2 TF (YY2), v-rel reticuloendotheliosis viral oncogene homolog A (RELA), signal transducer and activator of transcription 3 (STAT3), Jun proto-oncogene (JUN), aryl hydrocarbon receptor nuclear translocator-like (ARNTL), and cAMP responsive element binding protein 3-like 1 (CREB), CUGBP Elav-like family member 2 (CELF2), ELK3, and CCAAT/enhancer binding protein Δ (CEBPD), runt, myelocytomatosis, and upstream. Although only a small subset of mRNAs is associated with the differential expression after NIR-tPBMT, this allows for the identification of potential transcriptional regulators related to the changes in mRNA expression, which may contribute to a distinct transcriptional response to NIR light.

Investigating biological processes, we used our RNA-Seq data after light exposure (Fig 1A) to study the gene expression of the Kyoto Encyclopedia of Genes and Genomes (KEGG) database involved in metabolic pathways. Analysis of these 1,005 genes in the hippocampus and 1,482 genes in the cortex reveals that these differentially expressed genes (Table B-D in S3 File) have been significantly enriched in the pathways associated with angiogenesis, adipogenesis, allograft rejection, apical surface and junction, apoptosis, cholesterol homeostasis, coagulation, complement, DNA repair, epithelial mesenchymal transition, E2F and myelocytomatosis targets, glycolysis, G2M checkpoint, hypoxia, metabolism (bile acid, fatty acid, HEME, and xenobiotic), mitotic spindle, myogenesis, oxidative phosphorylation, pancreas beta cells, peroxisome, responses (androgen, estrogen, inflammatory, IFN-α, IFN-γ, UPR, and UV), signaling (IL-2-STAT5, IL-6/JAK/STAT3, hedgehog, KRAS, mTORC1, NOTCH, PI3K/AKT/mTOR, and Wnt/β-catenin), protein secretion, P53, ROS pathway, spermatogenesis, transforming grow factor β and tumor necrosis factor α (TNFα) signaling via nuclear factor κB (NF-κB).

**Fig 4:**
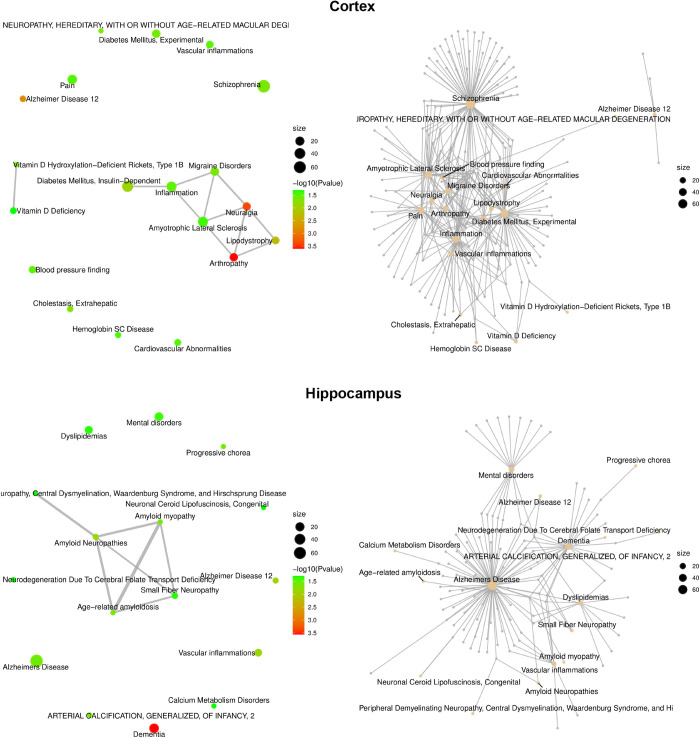
Network diagrams for the cortex and hippocampus modulated by 808 nm light, according to the sorting data of –log10 (*P*-value). **(Left)** Disease network diagram, where the ratio of the number of overlapping genes to the number of unique genes of the two is ≥ 20%, a node size indicates the total number of candidate genes belonging to a disease, and the color indicates –log10 (*P*-value). **(Right)** Network diagram of the disease and candidate genes, where a node size indicates the total number of candidate genes belonging to a disease. The high-resolution diagrams can be found in Figs N and O in S3 File.

Additionally, GO-enriched analysis of terms shows that among the phenotype-related biological processes of GO, there are 768 and 1,077 genes of pathways for the cortex and hippocampus, respectively (Figs J-M in S3 File). Among them, 20 pathways in the cortex and 18 in the hippocampus are associated with neurodegeneration. Network diagrams of diseases for the cortex and hippocampus are presented in Fig 4, respectively (the list of all genes corresponding to each of these pathways is presented in GSA, PRJCA022123). Focusing on preventing neurodegeneration and maintaining normal brain conditions using red/NIR-PBMT, we analyzed pathways like oxidative stress, neuroinflammation, neurogenesis, and neuronal apoptosis.

### NIR-tPBMT changes expression of genes and proteins related to oxidative stress

Oxidative stress can serve as either a basis for the initiation of neurodegenerative diseases via the generation of ROS and the release of pro-inflammatory cytokines [46], stimulating an excessive Aβ production or an activator of its progression and increasing the expression of amyloidogenic γ-secretase subunits such as BACE and PSEN [47,48]. The JUN activity is regulated by JNKs, stimulating a change in the redox potential. The combination of JUN and c-FOS forms a redox-sensitive activator protein (AP1) sensitive to ROS generated during oxidative stress. The BACE gene expression is partially regulated by TFs like AP1 and NF-κB, so the upregulation in the JUN expression can increase the secretion of BACE1 and PS1 [49].

**Fig 5:**
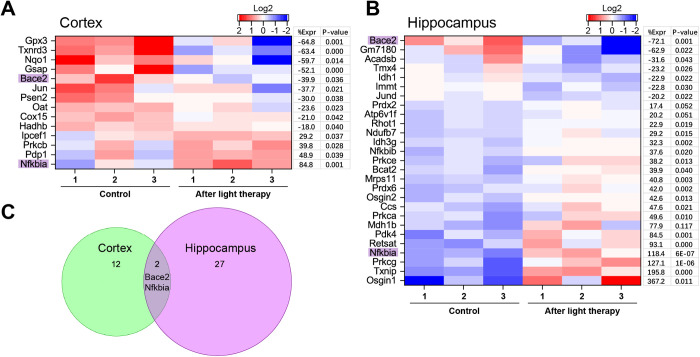
Oxidative stress modulated by 808 nm tPBMT in mice. Oxidative stress RNA-seq heatmaps of light-modulated genes for the cortex **(A)** and hippocampus **(B)**, showing the gene expression log2 level, percentage of gene expression (%Expr), and *P*-value. **(C)** A Venn diagram showing the overlap of two genes between the cortex and hippocampus results.

**Fig 6:**
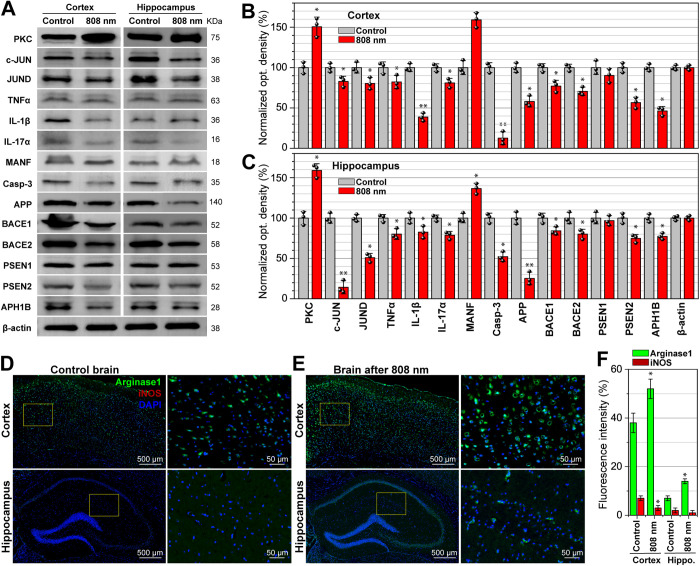
Western blotting of proteins and immunohistochemical analysis of microglia phenotype for the mouse brain after 808 nm tPBMT. **(A)** WB protein bands and their normalized integrated optical density for **(B)** the cortex and **(C)** hippocampus areas. The representative fluorescence microscopy images of the brain tissue **(D)** before and **(E)** after 808 nm tPBMT, stained with Arginase1 antibody (green), iNOS antibody (red), and DAPI (blue); and **(F)** the relative fluorescence intensities of the antibodies. The data are presented as M ± SD (*N* = 3), *P < 0.05, **P < 0.01 for data with a statistically significant difference.

Sorting out the genes relevant to the modulation of oxidative stress according to GO, 12 genes in the cortex and 27 in the hippocampus changed their RNA expression after NIR-tPBMT (Fig 5). The protein expression was additionally measured using WB analysis (Fig 6A–C). In particular, there was an increase in the expression of NF-κB inhibitor α (NF-κBIα), which encodes proteins preventing TF NF-κB from transporting into the nucleus and preserving it sequestered in an inactive state. An enlarged ROS generation removes the inhibition of NF-κB, which ensures its translocation into the nucleus [46]. Although we found a decreased RNA expression of JUN in the cortex and JUND in the hippocampus, WB confirmed a decreased protein expression of both с-JUN and JUND in both brain regions. Remarkably that the BACE1/2 and PSEN2 expressions were also suppressed after tPBMT.

On the other hand, the activity of many proteins depends on redox potential, so they can be deactivated by elevated concentrations of ROS at oxidative stress. One such protein is protein kinase C (PKC) because it contributes to the activation of α-secretase and nonamyloidogenic α-secretase-mediated APP cleavage and subsequently reduces Aβ plaque formation [50]. An increased expression of RNA/protein of PKC (the Prkca, Prkcb, Prkce, and Prkcg genes) was observed in both experimented brain regions after tPBMT.

### NIR-tPBMT modulates expression of genes and proteins related to neuroinflammation and neurogenesis

**Fig 7:**
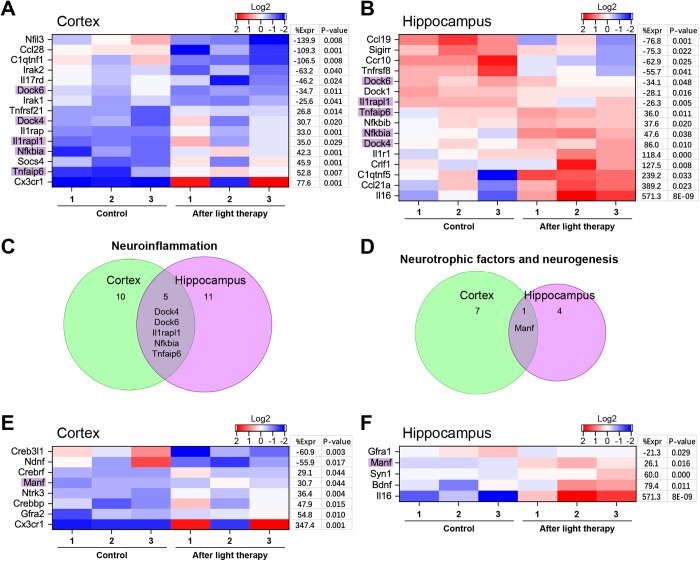
Neuroinflammation inhibition and neurogenesis activation by 808 nm tPBMT in mice. **(A,B)** Neuroinflammation and **(E,F)** neurotrophic factors/neurogenesis heatmaps of light-modulated genes for the cortex **(A,E)** and hippocampus **(B,F)**, showing gene expression log2 level, percentage of gene expression (%Expr), and *P*-value. A Venn diagram showing the overlap of **(C)** five genes for neuroinflammation and **(D)** one gene for neurotrophic factors/neurogenesis between the cortex and hippocampus data.

Neuroinflammation is an intrinsic brain response mediated by chemokines, cytokines, ROS, and other messengers. It is closely linked to the immune mechanisms of the tissues since various neuronal cell damages cause microglial activation, leading to the secretion of pro-inflammatory cytokines [46]. In our results, light modulated the expression of 10 genes in the cortex and 11 genes in the hippocampus (Fig 7A–C), which are relevant to inflammation, according to GO. Five of these genes overlap both brain regions: X-linked interleukin-1 (IL-1) receptor accessory protein-like 1 (IL-1RAPL1), TNFα induced protein 6 (TNFαIP6), NF-κBIα, dedicator of cytokinesis 4 (DOCK4), and DOCK6.

WB analysis showed a decreased expression of pro-inflammatory cytokines TNFα, IL-1β, and IL-17α after tPBMT (Fig 6A–C). At the same time, the RNA expression of the TNFαIP6 gene was increased after tPBMT (Fig 7A–C). TNFαIP6 can be induced by pro-inflammatory cytokines, such as TNFα and IL-1, probably as a protector of inflammation. TNFαIP6 modulates the polarization of macrophages/microglia from pro- to anti-inflammatory phenotype [51]. Light therapy increased the expression of the IL-1RAPL1 gene in the cortex and slightly decreased it in the hippocampus. IL-1RAPL1 encodes the IL-1 accessory protein, which also plays a role in supporting the memory system and learning abilities [52]. Both the transcriptome and WB analyses showed that IL-17 was downregulated in the brain after tPBMT. IL-17, encoded by the IL-17RD gene, is a highly versatile pro-inflammatory cytokine that regulates the activity of NF-κB and MAPK and is involved in various inflammatory diseases [53]. NIR light also increased the expression of NF-κBIα, which is involved in the regulation of pro-inflammatory cytokines via NF-κB activity [46].

NIR light increased the DOCK4 gene expression, especially in the hippocampus, while the DOCK6 gene expression decreased in both regions. These genes are guanine nucleotide exchange factors, which are activators of small G-proteins, vascular development, and cell proliferation. In particular, DOCK4, as a part of the trimeric complex, is required for the normal development of neuron dendrites and the regulation of cell proliferation [54].

To assess the inflammatory processes in a normal mouse brain and their changes after 808-nm tPBMT, we performed immunohistochemical analysis of microglia phenotype (Fig 6D–F). Arginase 1 and iNOS immunoreactivity were used as markers for microglia polarized to anti- and pro-inflammatory phenotypes, respectively [45]. The control brain had predominantly inactivated (45%) or anti-inflammatory microglia (38%) and a minor amount of pro-inflammatory microglia (7%). Since there was initially a small amount of pro-inflammatory microglia, the effect of tPBMT was negligible, yet anti-inflammatory microglia increased by 14% in the cortex and 7% in the hippocampus. It should be noted that TNFαIP6, which modulates the microglia from a pro- to anti-inflammatory phenotype [51], was upregulated by tPBMT.

Inflammatory processes that occur in patients with neurodegeneration disorders have a destructive effect on neuronal cells. Neurotrophic factors, including brain-derived neurotrophic factor (BDNF), neuronal growth factor (NGF), glial cell-derived neurotrophic factor (GDNF), mesencephalic astrocyte-derived neurotrophic factor (MANF), and others, can mitigate neurodegeneration and contribute to the restoration of damage [28,34]. Although we observed only an increased expression of BDNF in the hippocampus, our attention was drawn to an increased expression of the MANF gene in the cortex and hippocampus (Figs 6A–C and 7D–F ), which contributes to the maintenance of neuronal vital activity [55].

### NIR-tPBMT changes expression of genes and proteins related to neuronal apoptosis

**Fig 8:**
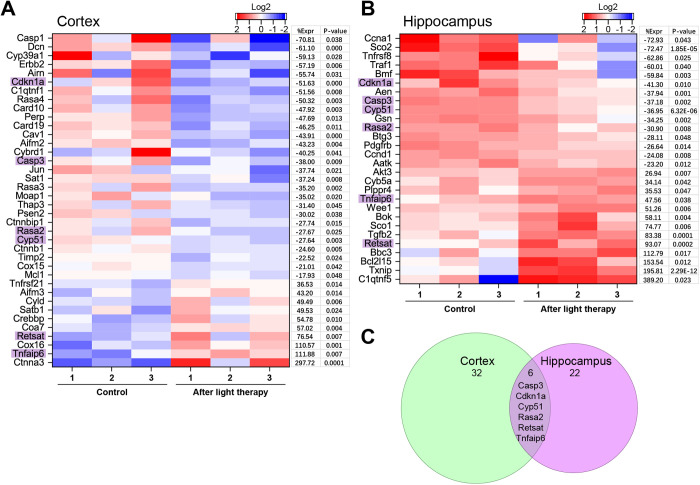
Neuronal apoptosis suppressed by 808 nm tPBMT in mice. Neuronal apoptosis processes heatmaps of light-modulated genes for the cortex **(A)** and hippocampus **(B)**, showing gene expression log2 level, percentage of gene expression (%Expr), and *P*-value. **(C)** A Venn diagram showing the overlap of six genes between the cortex and hippocampus data.

Activated apoptosis and accelerated brain cell death are hallmarks of neurodegenerative conditions. In our experiments, the transcriptome analysis revealed a change in the expression of new genes: 32 in the cortex and 22 in the hippocampus, which may play an additional role in apoptosis regulation and neurodegeneration development (Fig 8). Six of these genes overlap both brain regions: caspase-3 (Casp-3), TNFαIP6, cyclin-dependent kinase inhibitor 1A (CDKN1A), cytochrome P450 Family 51 (CYP51), RAS P21 protein activator 2 (RASA2), and retinol saturase (RESTAT).

The Casp-3 gene expression was downregulated by NIR-tPBMT in both brain regions (Fig 8). WB also confirmed a decrease in the Casp-3 concentration, particularly by 19% in the cortex (Fig 6A–C). The programmed cell death is triggered via the activation of the caspase pathway with the major role of mitochondria [56]. This principally proves a known light-induced suppression of apoptosis via the caspase pathway.

Additionally, an activated expression of the TNFαIP6 gene was detected after NIR-tPBMT. TNFαIP6 is known to participate in the inhibitory activity of serine IαI proteases, reducing inflammation and apoptosis [57]. Similarly, NIR light can activate the transcription of the AKT3 gene in the hippocampus. AKT3 protein, the most active in the nervous system, is a key regulator of a chemical signaling called the PI3K-AKT-mTOR pathway, which is essential for the normal development of many parts of the body, including the brain, as well as in the activation of anti-apoptotic pathways [58,59].

NIR light reduced the expression of CDKN1A. CDKN1A is a protein whose concentration is known to be increased during the resting phase of cells, acting as a regulator of cell cycle progression at the G1 phase and promoting DNA repair. On the contrary, a decreased concentration of CDKN1A in cells is associated with the activation of cell cycle [60].

Also, the Retsat gene expression was upregulated by NIR-tPBMT. The Retsat gene participates in the metabolic process of retinol. Retinol metabolite (retinoic acid) is an important biologically active molecule for neurogenesis and neuronal functional activity. A decreased level of retinoic acid and its receptors is accompanied by a decrease in cognitive abilities at the development of neurodegeneration [61].

### NIR-tPBMT modulates AD risk gene and protein expression

AD development is associated with the amyloidogenic and non-amyloidogenic pathways of proteolytic cleavages of transmembrane APP. The non-amyloidogenic pathway is associated with the cleavage of APP by the enzyme α-secretase, which prevents the formation of toxic Aβ peptides. The amyloidogenic pathway is associated with two membrane-bound proteases, namely, aspartic protease BACE1 and γ-secretase. The γ-secretase complex is formed by four subunits: PSEN1/2, NCT, APH-1, and PEN-2 [62]. As a result of the sequential cleavages of APP by these secretases, toxic Aβ40/42/43 peptides are produced. The overexpression of PSENs causes γ-secretase activation, triggering Aβ-42(43) secretion in the brain [63]. On the contrary, a decreased content of PSEN subunits reduces the production of Aβ in neurons [47]. The inhibition of the expression of the γ-secretase subunits, including PSENs, is now an important strategy to reduce Aβ production in AD patients. However, there are almost no drugs for clinical use aimed at blocking the expression/activity of BACE or γ-secretase.

**Fig 9:**
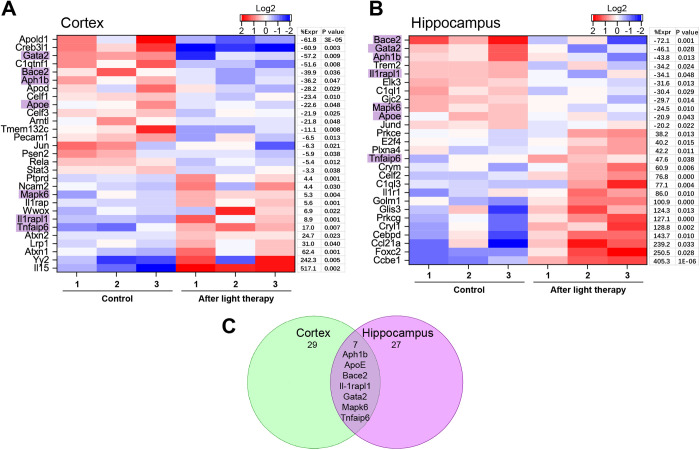
AD risk gene expression modulated by 808 nm tPBMT in mice. Alzheimer’s disease risk gene expression heatmaps of light-modulated genes for the cortex **(A)** and hippocampus **(B)**, showing gene expression log2 level, percentage of gene expression (%Expr), and P-value. **(C)** A Venn diagram showing the overlap of seven genes between the cortex and hippocampus results.

In our experiments (Fig 9), WB showed a decrease in BACE1, BACE2 (a close homologue of BACE1), PSEN2, and APH1 homolog B γ-secretase subunit (APH1B) in both brain regions after tPBMT (Fig 6A–C). At the same time, the expression of BACE2 in both the cortex and hippocampus was reduced, while a significant decrease in the expression of PSEN2 γ-secretase subunits occurred only in the cortex. The expression of APH1B was also reduced in both brain regions. This subunit is the stabilizing cofactor of PSEN and also affects the activity of γ-secretase [63]. Importantly, WB showed a decreased APP by 42% and 75% after tPBMT in the cortex and hippocampus, respectively (Fig 6A–C); at the same time, the transcriptome analysis did not distinguish a significant reduction in APP expression.

Also, according to the transcriptome analysis, NIR light modulated the expression of transcriptional genes that are risk genes for AD [64]: they are GATA2, YY2, RELA, STAT3, JUN, ARNTL, and CREB3L1 in the cortex; and CELF2, GATA2, E2F4, ELK3, CEBPD, and JUND in the hippocampus. The expression changes also occurred in metabolic AD risk genes and transporters: BACE2, PSEN2, APH1B, apolipoprotein D (ApoD), apolipoprotein L domain containing 1 (APOLD1), ataxin 1 (ATXN1), ATXN2, transmembrane protein 132C (TMEM132C), CELF1/3, neural cell adhesion molecule 2 (NCAM2), WW domain-containing oxidoreductase (WWOX), platelet/endothelial cell adhesion molecule 1 (PECAM1), protein tyrosine phosphatase receptor type D (PTPRD), TNFαIP6, IL-1R accessory protein (IL-1RAP), IL-1RAPL1, MAPK6, low density lipoprotein receptor-related protein 1 (LRP1), and C1q tumor necrosis factor related protein 1 (C1QTNF1) in the cortex; and BACE2, golgi membrane protein 1 (GOLM1), plexin A4 (PLXNA4), GLIS family zinc finger 3 (GLIS3), triggering receptor expressed on myeloid cells 2 (TREM2), protein kinase C ε (PRKCE), crystallin λ1 (CRYL1), chemokine C-C motif ligand 21A serine (CCL21A), forkhead box C2 (FOXC2), collagen and calcium binding EGF domains 1 (CCBE1), gap junction protein γ2 (GJC2), TNFαIP6, IL-1R1, IL-1RAPL1, MAPK6, complement component 1 q subcomponent-like 1 (C1QL1), and C1QL3 in the hippocampus.

## Discussion

Neurodegenerative diseases are multifaceted disorders, which imperceptible pathological processes begin from disruption of brain cell metabolism, oxidative stress, neuroinflammation, after which the misfolded proteins are accumulated ensuing neuron death. The studies of brain gene expression are needed to identify early molecular mechanisms underlying the onset of neurodegenerative diseases to develop treatments for mental and neurological disorders. Our experiments were conducted on BALB/c mice as a model of a normal healthy organism, aiming to study possible changes in gene transcription after tPBMT and to analyze changes in the expression of AD risk genes. This seems an important option in assessing the light effect on the brain, finding new molecular mechanisms of tPBMT against neurodegenerative diseases, and inhibiting their development using noninvasive therapy. The photoneuromodulation technique uses light to influence energy metabolism pathways in the brain. The whole RNA-Seq study of the expression of the brain is extremely helpful to elucidate all altered molecular mechanisms. Our results show that numerous genes, associated with about 20 pathways of neurodegeneration including oxidative stress, neuroinflammation, neurogenesis, and neuronal apoptosis, are activated after tPBMT in the cortex and hippocampus.

Particularly, RNA-Seq and WB analyses showed a light-induced change in the transcription of the number of genes involved in the regulation of oxidative stress. In particular, an increased expression of PKC and NF-κBIα (a NF-κB inhibitor) and a decreased expression of JUN were observed in both experimented brain regions. The BACE1/2 expression was also suppressed after tPBMT, possibly due to an altered expression of JUN and NF-κBIα. Therefore, the preservation of normal redox metabolism or the reduction in oxidative stress may be potentially realized by inhibiting the NF-κB signaling pathway using PBMT [27,46,65]. This can also contribute to sustaining a physiological concentration of Aβ in the brain.

Our results also showed the photomodulation of the expression of genes and cytokines, which are related to inflammation (NF-κBIα, TNFα, IL-1β, IL-17, and DOCK), regulation of cell neurogenesis (DOCK, BDNF, and MANF), polarization of microglia from a pro- to anti-inflammatory phenotype (TNFαIP6), development of vessels and neuron's dendrites (DOCK), and the effects of thermogenesis on memory and learning systems (IL-1RAPL1). Also, microglia reliably shifted to the anti-inflammatory phenotype in the cortex and hippocampus after tPBMT. It should be added that NIR light was previously reported to reduce the secretion of pro-inflammatory cytokines and stimulate that of anti-inflammatory cytokines [5,19,26,30–33,35]. Particularly, PBMT can reduce pro-inflammatory cytokines by inhibiting the NF-κB signaling pathways, resulting in mitigated inflammatory responses [66,67]. Also, it can be assumed that NIR-PBM can help strengthen the extracellular matrix, the violation of which occurs during inflammation [68]. The effects of damage-repairing neurotrophic factors explain the interest in studying PBMT aimed to alter their expression in the brain [69,70]. Particularly, an upregulated MANF has not previously been recorded after NIR light exposure; the uniqueness of this gene is that it selectively promotes the survival of neurons and inhibits cell death induced by endoplasmic reticulum stress/depletion [71]. Therefore, our results may support and uncover possible pathways associated with the NIR-PBMT ability to restore neurons [11,18,34,72], improve spatial memory [12], and preserve cognitive skills [13] in neurodegenerative disorders. Previous studies on tPBMT [5,19,29–35] support the idea of a new strategy for neuroprotection, which is due to a decrease in the expression of pro-inflammatory cytokines and an increase in the expression of anti-inflammatory cytokines, as well as neurotrophic factors.

The activation of apoptosis and accelerated death of neurons are typical features of neurodegenerative diseases. We found many apoptosis-related genes that were photomodulated (Casp-3, TNFαIP6, CDKN1A, CYP51, RASA2, and RESTAT). This confirms previous observations in various in vitro models of neurotoxicity on neuronal survival that red/NIR light can significantly decrease neuronal apoptosis by reducing pro-apoptotic factors, such as Bcl-2 antagonist X (Bax), Bcl-2-associated death promoter (BAD), and caspase-3 [34,36–38,59]. Also, NIR light turned out to be a novel modulator of retinol metabolism in the brain, targeting the preservation of neuronal survival. Furthermore, NIR light may modulate the DNA repair pathways to prevent apoptosis and age-related neurodegenerative diseases. It should be emphasized that the transcriptional changes found in most of these genes following tPBMT have not been previously reported. We believe that such identified gene transcriptional changes of numerous genes may help to explain the initial mechanisms of red/NIR light neuroprotective properties with the reduced apoptosis and inflammation, previously reported [5,6,9,11,19,26–38].

We also focused on the effect of tPBMT on the expression of AD risk genes and molecular networks involved in related pathological processes. We found that NIR light reduces the expression of TFs, which are key players in the formation of Aβ and misfolded proteins, including APP. They are BACE1, BACE2, PSEN2, APH1B, JUN, GATA2, YY2, RELA, STAT3, ARNTL, CREB3L1, CELF2, E2F4, ELK3, CEBPD, and many others. These genes are of interest because the proteins they encode can regulate many cellular metabolic pathways, including neuronal survival and plasticity. It was reported that genes that are TFs change their expression in the blood of patients with mild cognitive impairment and AD dementia [64].

Red/NIR-tPBMT is known to alleviate Aβ burden and plaque development [2–11], which both accelerate neurodegeneration and oxidative damage. Meanwhile, the available literature lacks information on the light effect on the expression of key genes associated with the risk of AD, such as APP, BACE, and γ-secretase subunits. We showed that 808-nm tPBMT can downregulate AD-risk genes BACE, PSEN, and APP in APOE mice [6]. These proteins in the amyloidogenic pathway are all Ca^2+^-dependent [73,74]; thus, their light-induced downregulation is probably controlled by light-induced Ca^2+^ cellular uptake and Ca^2+^/calmodulin signaling via the PKA activation [6]. Furthermore, NIR-tPBMT was reported to reduce the BACE and APP gene levels after the triggering of the cAMP/PKA and PKA/sirtuin-1 signaling pathways [5]. Also, it can suppress JNK3 by the activation of the extracellular signal-regulated kinase and MAPK-phosphatase-7 signaling [25]. Yet, prior to our study, it was unclear how PBMT affects the expression of genes associated with AD onset in healthy, non-aged mice. Our previous and current results indicate that NIR light can influence the key stages in Aβ generation and, as a consequence, can serve as an important tool to alleviate dementia in its early stages. Moreover, a constantly increasing number of reports indicates the unidirectional effect of tPBMT on different animal groups and mouse types.

Therefore, PBMT demonstrates positive therapeutic effects in mitigating neurodegenerative pathologies [1–14], showing its potential for clinical use. Our studies support the idea of a neuroprotective effect of tPBMT by activating TFs and gene expression. It provides an opportunity to understand the molecular processes underlying the influence of tPBM on neuronal metabolism, neurogenesis, and synapse formation, as well as the activation of anti-inflammatory and anti-apoptotic mechanisms. However, the question of whether it is possible to achieve similar effects in humans remains open. In tPBMT applications, achieving adequate dose delivery to provide optimal stimulation is not a trivial task due to the exponential decrease in light intensity as it passes through body tissues [75]. Moreover, the light dose delivered to deeper brain parts is always much smaller than that for the cortex due to a high scattering and multiple absorption of light. On the other hand, PBMT is a type of supportive therapy used in medicine. A systematic application of PBMT for months can substantially enhance the conditions of an unhealthy brain, skin, and other organs or facilitate healing, lower pain, etc., but it cannot cure some diseases [21,76]. So, PBM can be considered for daily use to maintain health. In this purpose, our results point out that tPBMT may potentially help inhibit the development of different forms of neurodegeneration or other diseases.

## Supporting information

S1 File The original uncropped and unadjusted images underlying of WB results. (PDF)

S2 File The raw data behind the graphics of Western blotting of proteins and immunohistochemical analysis. (PDF)

S3 File Methods and results. (PDF)
