# Supplementary material for: Transcranial photobiomodulation therapy with 808 nm light changes expression of genes and proteins associated with neuroprotection, neuroinflammation, oxidative stress, and Alzheimer’s disease: Whole RNA sequencing of mouse cortex and hippocampus
Source: PLoS One. 2025 Jul 18;20(7):e0326881. doi: 10.1371/journal.pone.0326881 (PMC12273915; doi:10.1371/journal.pone.0326881)
Supplement: S1 File — (PDF) [file pone.0326881.s001.pdf]

S1 raw images

Supporting Information

Transcranial photobiomodulation therapy with 808 nm light changes expression of genes and proteins associated with neuroprotection, neuroinflammation, oxidative stress, and Alzheimer's disease: Whole RNA sequencing of mouse cortex and hippocampus

Binjun Li [1], Iuliia Golovynska [1,*], Yurii V. Stepanov [2], Sergii Golovynskyi [1], Andrii Golovynskyi [3], Denis Kolesnik [2], Liudmyla I. Stepanova [4], Puxiang Lai[5], Fangrui Lin [1,*], Junle Qu [1]

[1] *Center for Biomedical Photonics, College of Physics and Optoelectronic Engineering, Key Laboratory of Optoelectronic Devices and Systems of Ministry of Education and Guangdong Province, Shenzhen University, Shenzhen 518060, P. R. China*

[2]*R.E. Kavetsky Institute of Experimental Pathology, Oncology and Radiobiology, NAS of Ukraine, Kyiv 03022, Ukraine*

[3]*V.M. Glushkov Institute of Cybernetics, NAS of Ukraine, Kyiv 03187, Ukraine*

[4]*Institute of Biology and Medicine, Taras Shevchenko National University of Kyiv, Kyiv 01601, Ukraine*

[5]*Department of Biomedical Engineering, The Hong Kong Polytechnic University, Hong Kong SAR, China*

[*] Corresponding author's e-mail: iuliia@szu.edu.cn (I. Golovynska), lfr1993@163.com (F. Lin)

The original uncropped and unadjusted images underlying of WB results

Protein Kinase C (PKC)

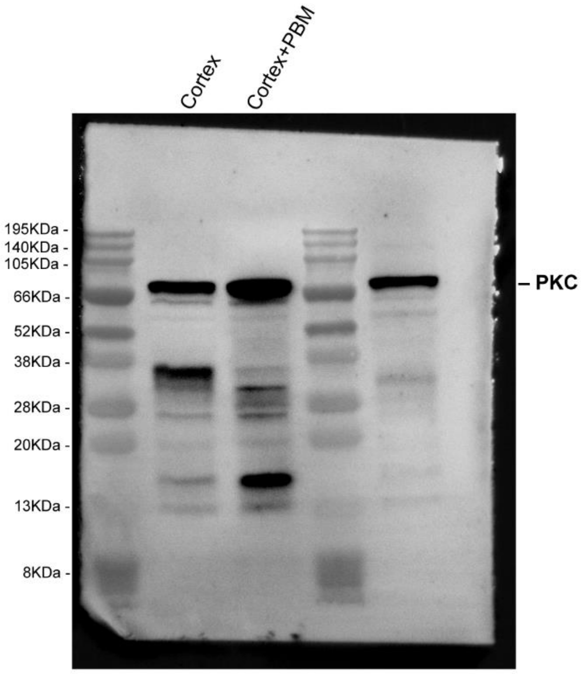

Protein Kinase C (PKC)

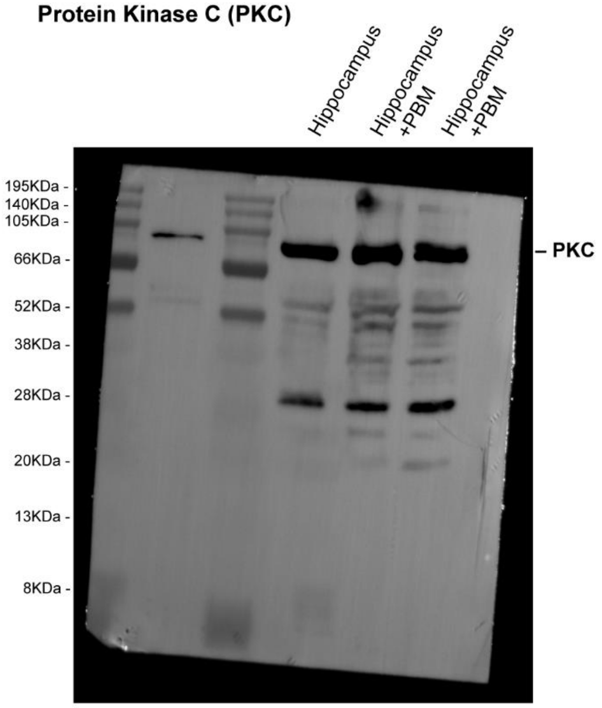

Jun Proto-Oncogene (c-JUN)

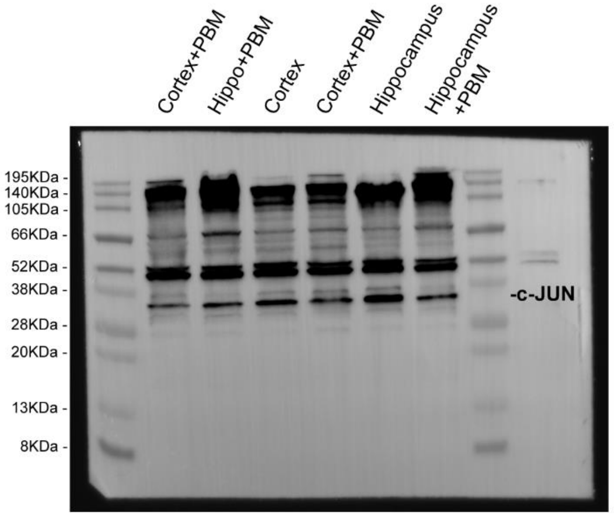

Jun D Proto-Oncogene (JUND)

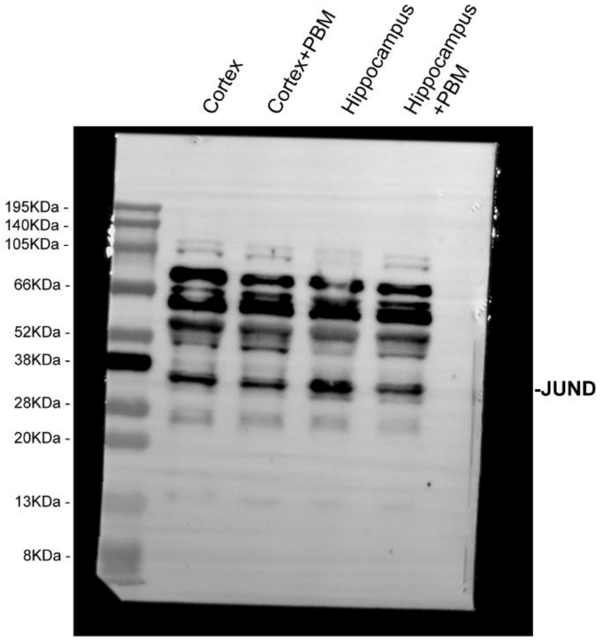

Mesencephalic Astrocyte-Derived Neurotrophic Factor (MANF)

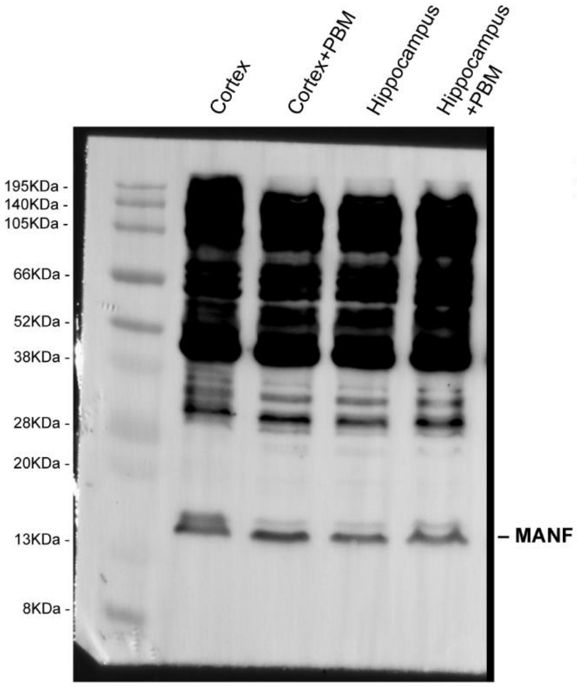

Caspase-3

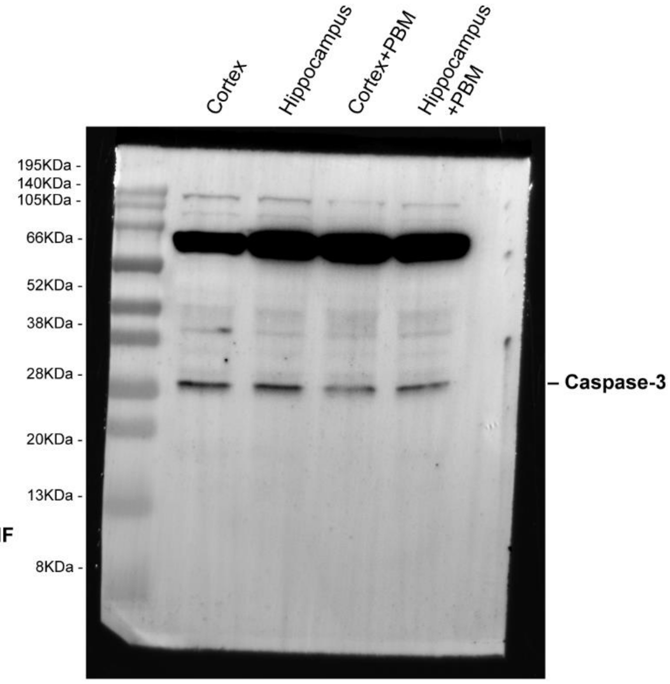

Amyloid Precursor Protein (APP)

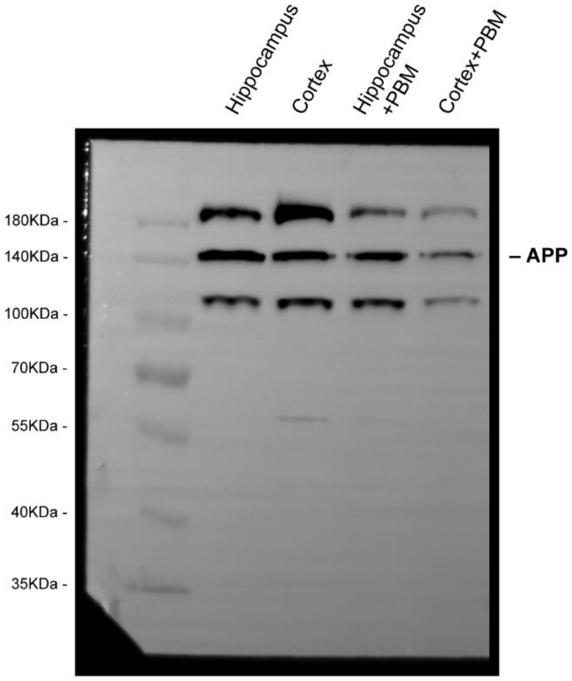

Aph1 Homolog B, Gamma Secretase Subunit

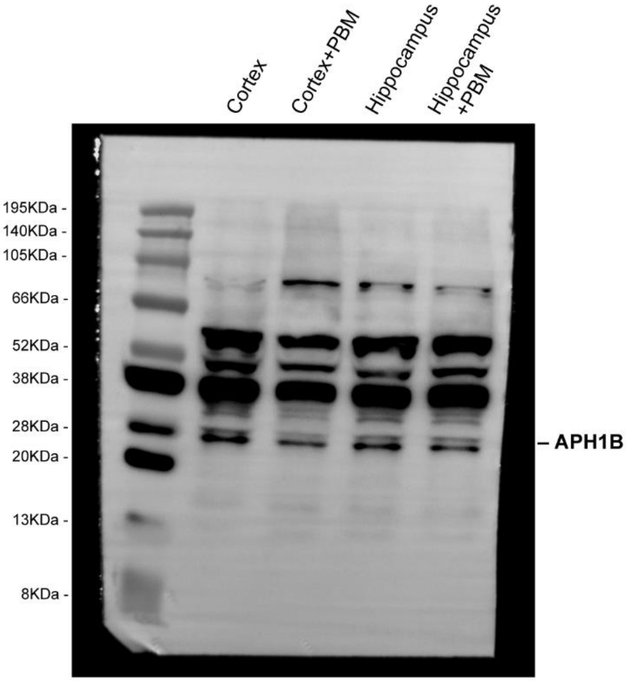

Presenelin-2 (PSEN2)

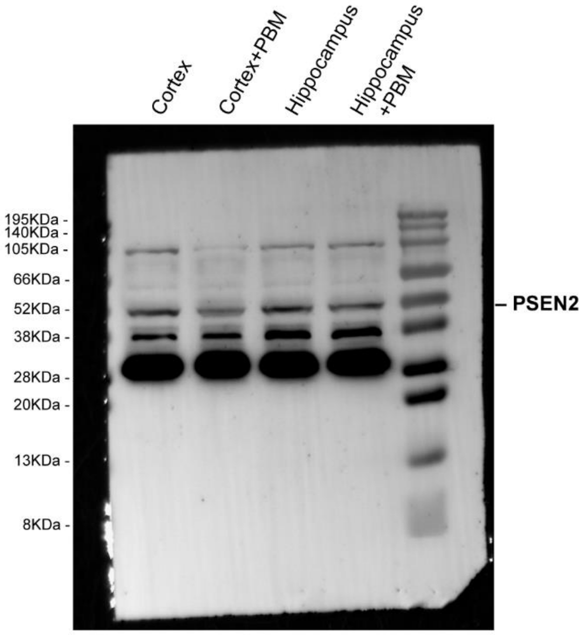

β-Secretase 1 (BACE1)

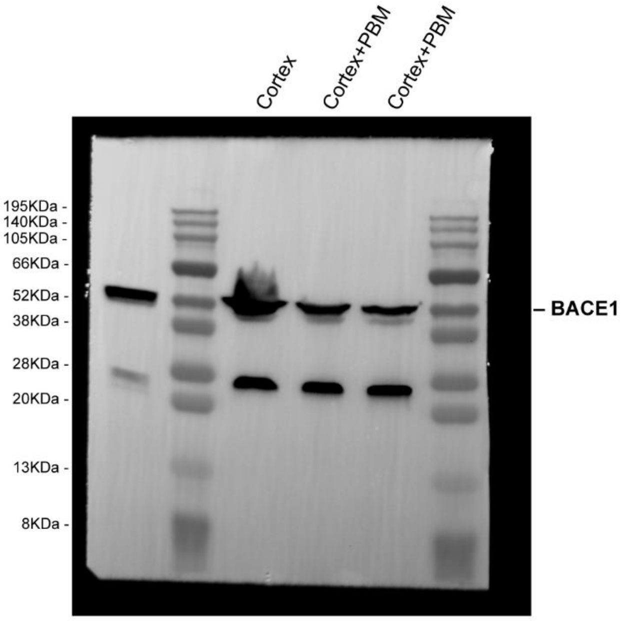

β-Secretase 2 (BACE2)

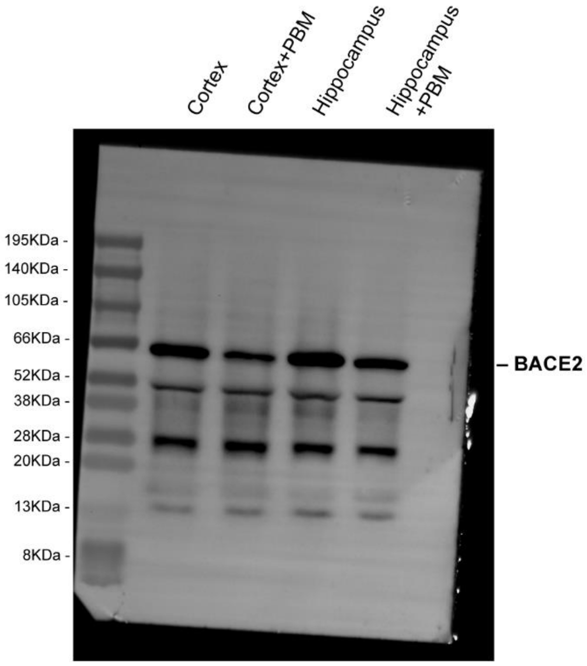

β-Secretase 1 (BACE1)

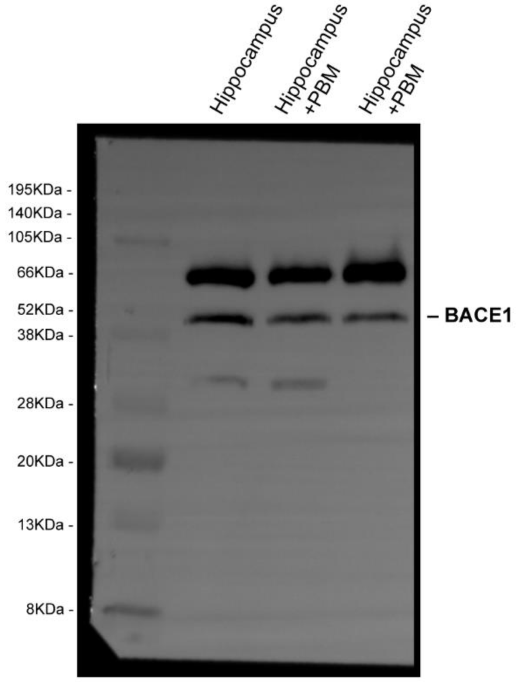

Interleukin-1β

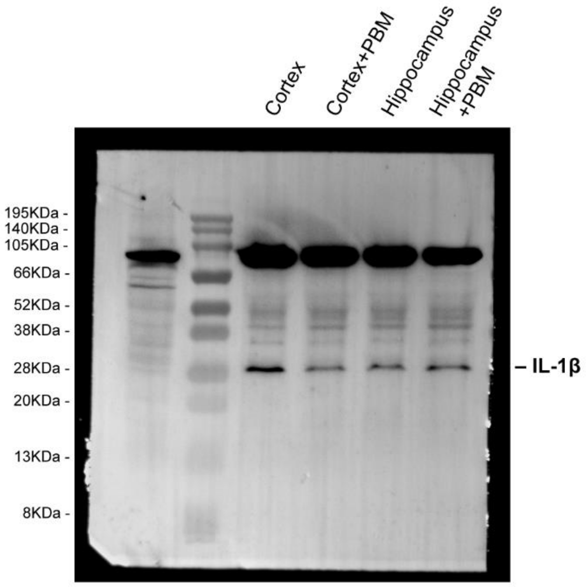

Tumor Necrosis Factor α

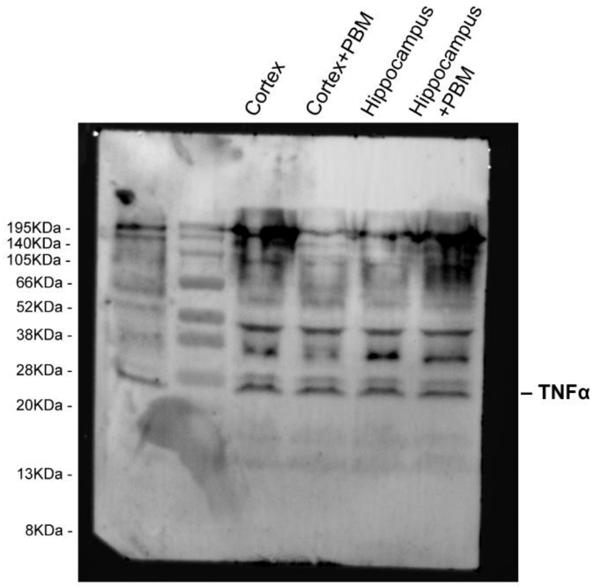

Presenelin-1 (PSEN1)

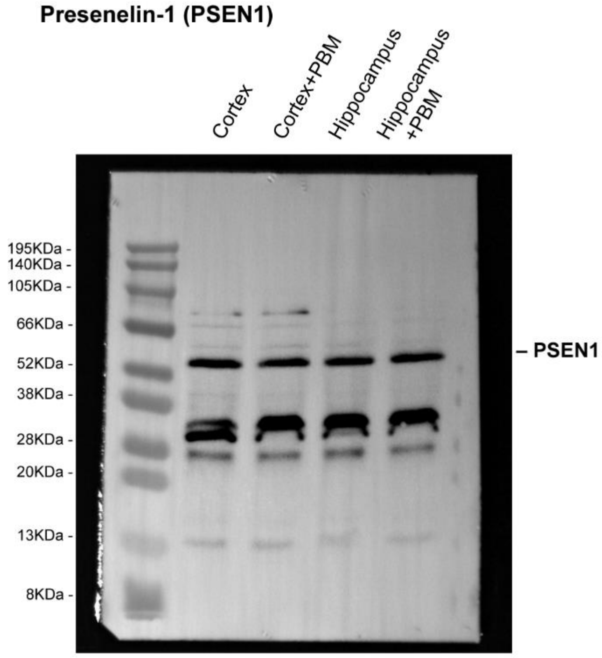

Interleukin-17α

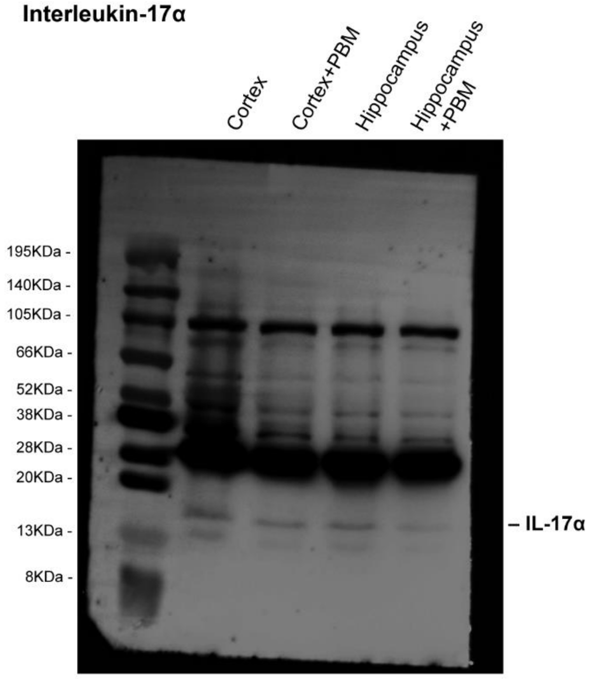

β-Actin

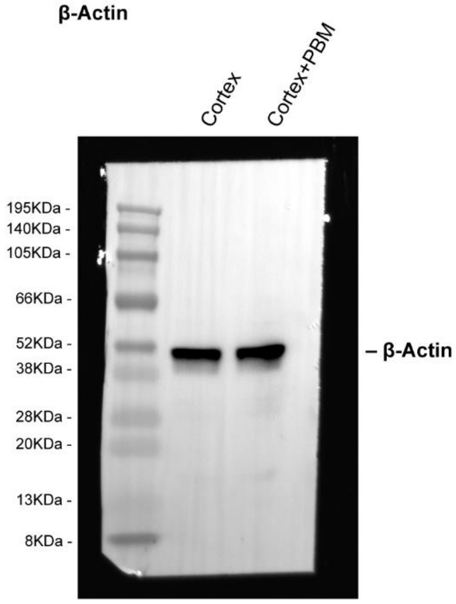

GAPDH

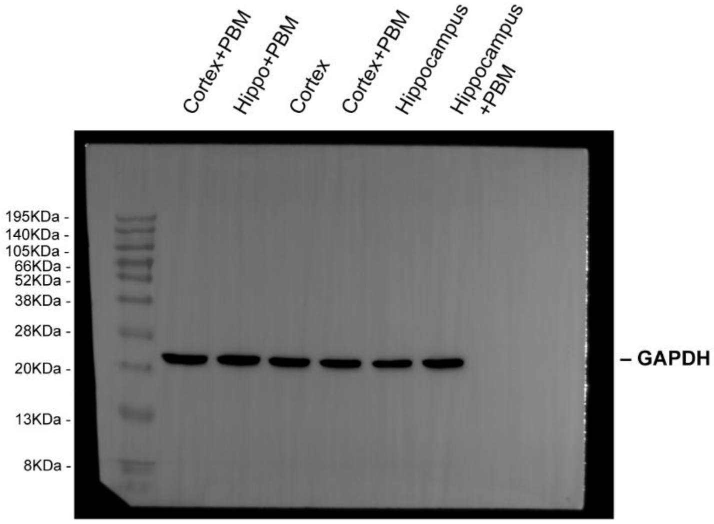

β-Actin

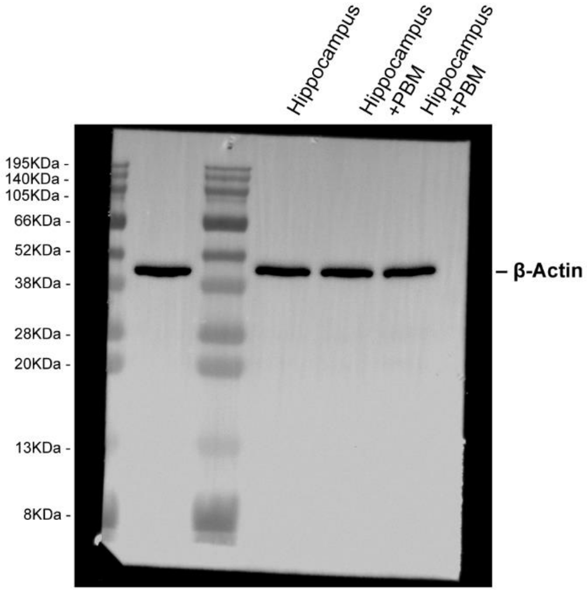

β-Actin

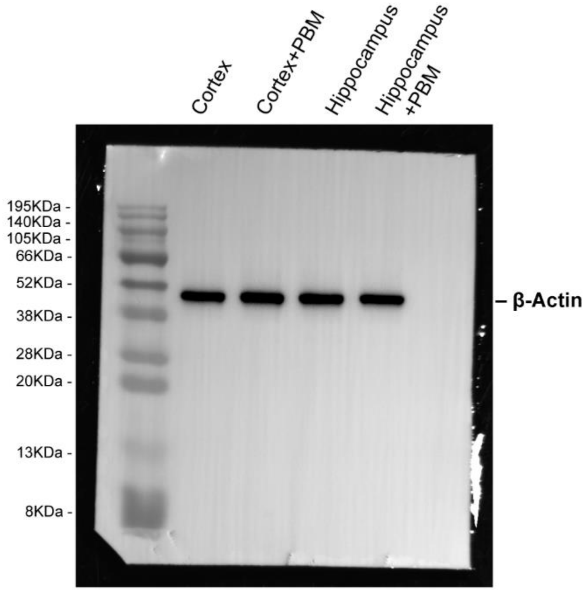

β-Actin

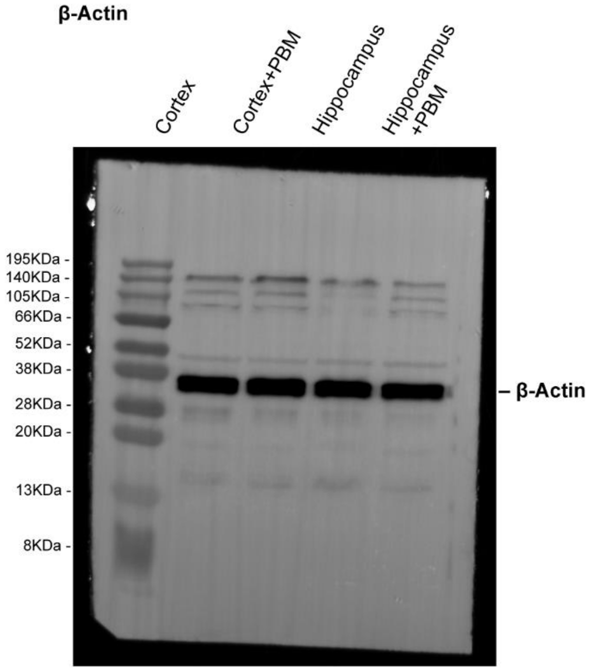

β-Actin

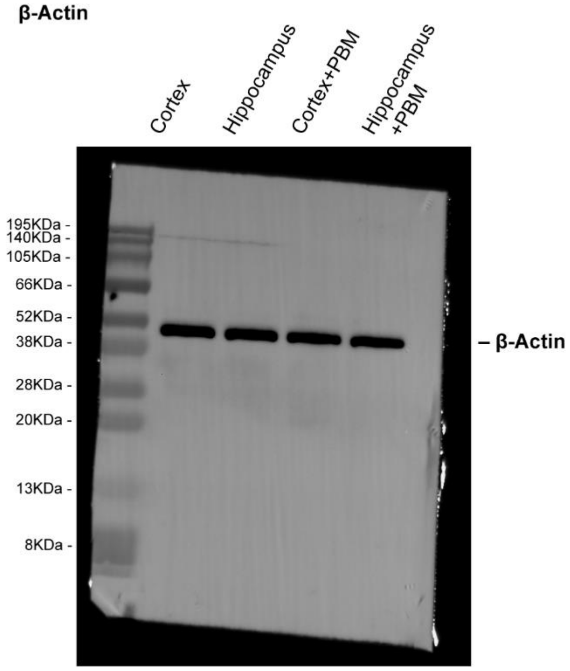

β-Actin

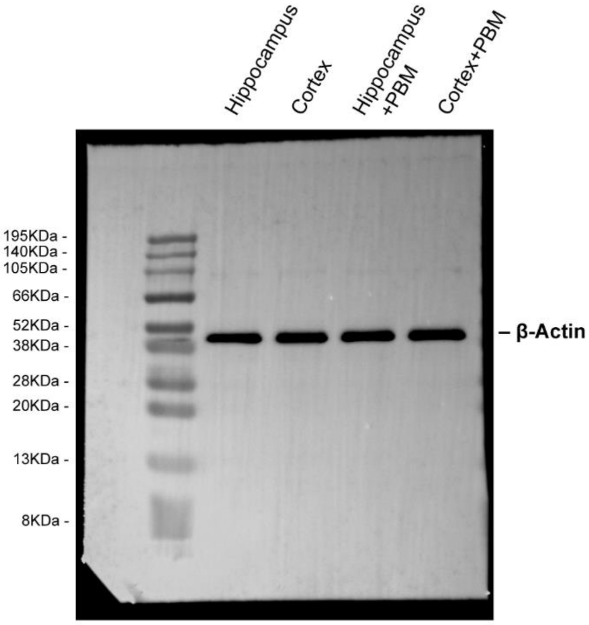

β-Actin

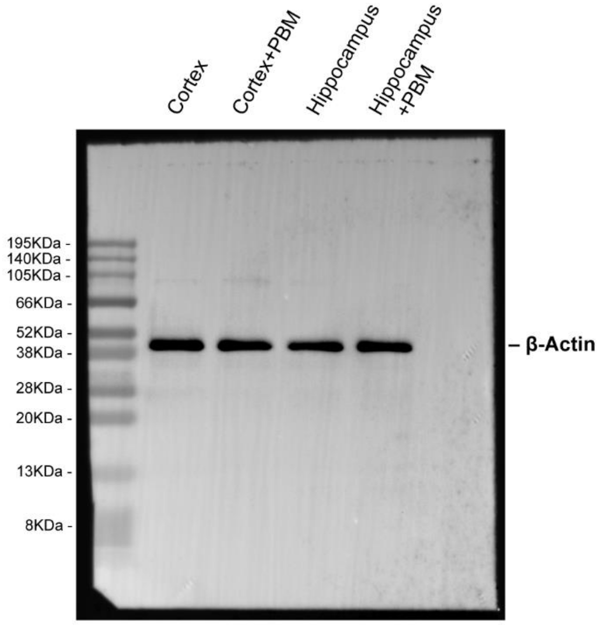

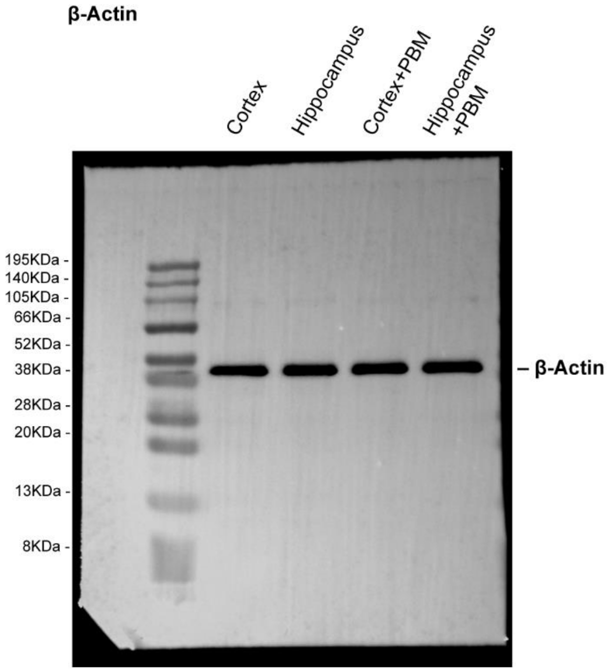
β-Actin
Cortex
Hippocampus
Cortex+PBM
Hippocampus +PBM
195KDa -
140KDa -
105KDa -
66KDa -
52KDa -
38KDa -
28KDa -
20KDa -
13KDa -
8KDa -
– β-Actin

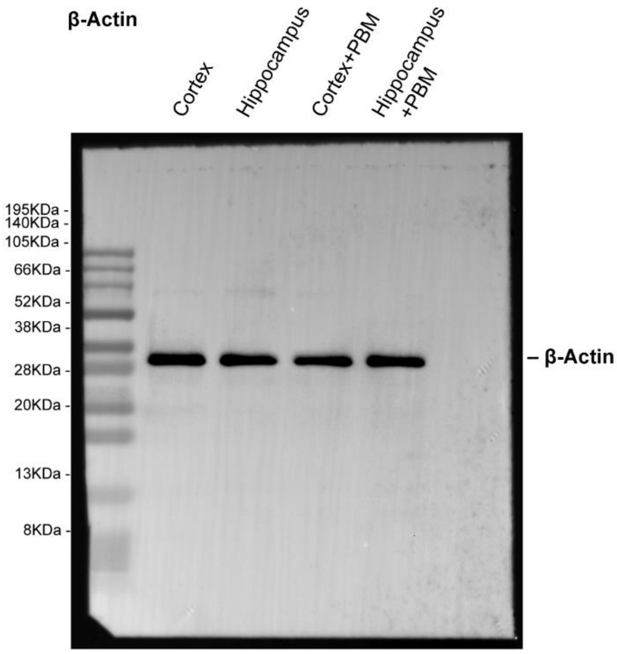
β-Actin
Cortex
Hippocampus
Cortex+PBM
Hippocampus +PBM
195KDa -
140KDa -
105KDa -
66KDa -
52KDa -
38KDa -
28KDa -
20KDa -
13KDa -
8KDa -
– β-Actin

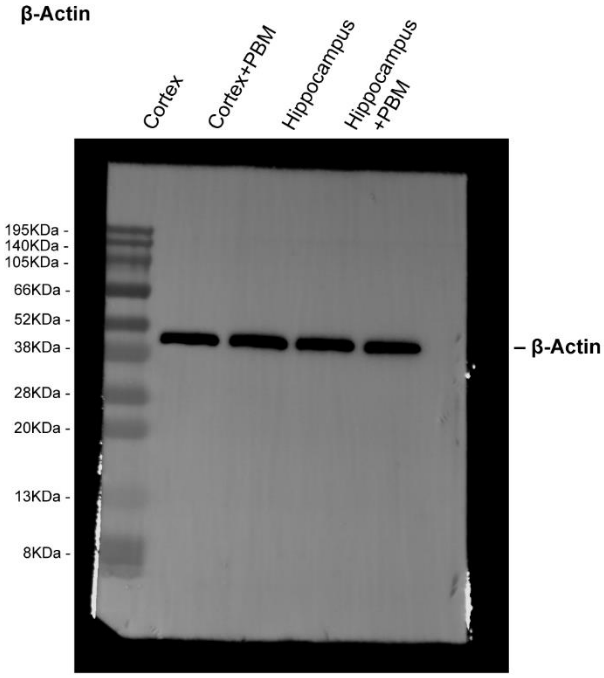
β-Actin
Cortex
Cortex+PBM
Hippocampus
Hippocampus +PBM
195KDa -
140KDa -
105KDa -
66KDa -
52KDa -
38KDa -
28KDa -
20KDa -
13KDa -
8KDa -
– β-Actin
