# Supplementary material for: Transcranial photobiomodulation therapy with 808 nm light changes expression of genes and proteins associated with neuroprotection, neuroinflammation, oxidative stress, and Alzheimer’s disease: Whole RNA sequencing of mouse cortex and hippocampus
Source: PLoS One. 2025 Jul 18;20(7):e0326881. doi: 10.1371/journal.pone.0326881 (PMC12273915; doi:10.1371/journal.pone.0326881)
Supplement: S2 File — (PDF) [file pone.0326881.s002.pdf]

S2 Raw Data

Transcranial photobiomodulation therapy with 808 nm light changes expression of genes and proteins associated with neuroprotection, neuroinflammation, oxidative stress, and Alzheimer's disease: Whole RNA sequencing of mouse cortex and hippocampus

Binjun Li [1], Iuliia Golovynska [1,*], Yurii V. Stepanov [2], Sergii Golovynskyi [1], Andrii Golovynskyi [3], Denis Kolesnik [2], Liudmyla I. Stepanova [4], Puxiang Lai[5], Fangrui Lin [1,*], Junle Qu [1]

[1] *Center for Biomedical Photonics, College of Physics and Optoelectronic Engineering, Shenzhen University, Shenzhen 518060, P. R. China*

[2]*R.E. Kavetsky Institute of Experimental Pathology, Oncology and Radiobiology, NAS of Ukraine, Kyiv 03022, Ukraine*

[3]*V.M. Glushkov Institute of Cybernetics, NAS of Ukraine, Kyiv 03187, Ukraine*

[4]*Institute of Biology and Medicine, Taras Shevchenko National University of Kyiv, Kyiv 01601, Ukraine*

[5]*Department of Biomedical Engineering, The Hong Kong Polytechnic University, Hong Kong SAR, China*

[*] Corresponding author's e-mail: iuliia@szu.edu.cn (I. Golovynska), lfr1993@163.com (F. Lin)

Table A. Raw data of Western blotting of different proteins evaluated as normalized integrated optical density for the mouse cortex and hippocampus brain areas before and after 808 nm tPBMT.

Protein	PKC	c-JUN	JUND	TNFα	IL-1β	IL-17α	MANF	Casp3	APP	BACE1	BACE2	PSEN1	PSEN2	APH1B	βactin
**Area**	**The cortex control**														
**1**	101	105.7	97.6	91.9	98	104.1	97	93.4	106.2	102	101	92.8	105.6	104.9	99.2
**2**	106.6	99.6	107.6	105.1	96.4	101	109.8	105.1	99	103.6	95.4	109.2	100.5	99	97.4
**3**	92.4	94.7	94.8	103	105.6	94.9	93.2	101.5	94.7	94.4	103.6	98	93.9	96.1	103.4
**Mean**	100	100	100	100	100	100	100	100	100	100	100	100	100	100	100
**SD**	7.2	5.5	6.7	7.1	4.9	4.7	8.7	6	5.8	4.9	4.2	8.4	5.9	4.5	3
**Area**	**The cortex after 808 nm tPBMT**														
**1**	153.6	84.9	73.6	80.2	44	82	167.9	10.6	65.7	69.1	66.5	98.2	62.8	49	96.9
**2**	137.4	88.1	88.2	91.3	38.2	86.2	149.4	5.8	55.5	79.1	68.6	81.2	57.6	39.9	103
**3**	160.7	75.7	79.4	75.1	34	74.8	160.2	21.4	52.8	83.2	76.6	91.3	49.4	50	100.1
**Mean**	150.6	82.9	80.4	82.2	38.7	81	159.2	12.6	58	77.1	70.6	90.2	56.6	46.3	100
**SD**	11.9	6.4	7.4	8.3	5	5.8	9.3	8	6.8	7.2	5.3	8.6	6.8	5.5	3.1
**Area**	**The hippocampus control**														
**1**	107.4	98	104.7	102	94	101	106.8	93.4	99	93.2	94.2	94.9	105.8	99.4	100.8
**2**	101.5	95	96.3	103.2	99	103.1	97	105.1	93	104.8	107.8	108.1	101	105.3	96.8
**3**	91.1	107	99	94.8	107	95.9	96.2	101.5	108	102	98	97	93.2	95.3	102.4
**Mean**	100	100	100	100	100	100	100	100	100	100	100	100	100	100	100
**SD**	8.6	6.2	4.3	4.5	6.6	3.7	5.9	6	7.5	6	7	7.1	6.4	5	2.9
**Area**	**The hippocampus after 808 nm tPBMT**														
**1**	165.2	10.8	45.1	87.7	82.1	73.4	129.6	58.7	23.2	80.4	81.8	103.6	76.6	81.6	100.2
**2**	162.4	23.6	55.3	77.7	90.4	82.2	137.4	50.7	33.9	89.9	85.2	91.4	78.7	77	96.9
**3**	149.4	8.5	52.6	75.2	75.3	80.8	142.8	47.2	18.5	82.2	73.6	95.4	69.1	73.6	102.9
**Mean**	159	14.3	51	80.2	82.6	78.8	136.6	52.2	25.2	84.2	80.2	96.8	74.8	77.4	100
**SD**	8.4	8.1	5.3	6.6	7.6	4.7	6.6	5.9	7.9	5	6	6.2	5	4	3

Table B. The relative fluorescence intensities of Arginase1 and iNOS antibodies used for staining the mouse cortex and hippocampus brain areas before and after 808 nm tPBMT.

**Area**	**The cortex**				**The hippocampus**			
**Experiment**	**Control**		**After 808 nm tPBMT**		**Control**		**After 808 nm tPBMT**	
**Marker**	**Arginase1**	**iNOS**	**Arginase1**	**iNOS**	**Arginase1**	**iNOS**	**Arginase1**	**iNOS**
**1**	33.7	5.6	47.8	3.2	6.3	0.9	15.3	0.8
**2**	39.3	7.4	55.9	3.8	8.1	2.1	13.3	0.4
**3**	41.3	7.9	52.5	2.1	6.4	3.2	13.7	2.1
**Mean**	38.1	7	52.1	3	6.9	2.1	14.1	1.1
**SD**	3.9	1.2	4.1	0.9	1	1.2	1.1	0.9
