# Supplementary material for: Transcranial photobiomodulation therapy with 808 nm light changes expression of genes and proteins associated with neuroprotection, neuroinflammation, oxidative stress, and Alzheimer’s disease: Whole RNA sequencing of mouse cortex and hippocampus
Source: PLoS One. 2025 Jul 18;20(7):e0326881. doi: 10.1371/journal.pone.0326881 (PMC12273915; doi:10.1371/journal.pone.0326881)
Supplement: S3 File — (PDF) [file pone.0326881.s003.pdf]

S3 Supporting Information

Methods and Results

Transcranial photobiomodulation therapy with 808 nm light changes expression of genes and proteins associated with neuroprotection, neuroinflammation, oxidative stress, and Alzheimer's disease: Whole RNA sequencing of mouse cortex and hippocampus

Binjun Li [1], Iuliia Golovynska [1,*], Yurii V. Stepanov [2], Sergii Golovynskyi [1], Andrii Golovynskyi [3], Denis Kolesnik [2], Liudmyla I. Stepanova [4], Puxiang Lai[5], Fangrui Lin [1,*], Junle Qu [1]

[1] *Center for Biomedical Photonics, College of Physics and Optoelectronic Engineering, Key Laboratory of Optoelectronic Devices and Systems of Ministry of Education and Guangdong Province, Shenzhen University, Shenzhen 518060, P. R. China*

[2]*R.E. Kavetsky Institute of Experimental Pathology, Oncology and Radiobiology, NAS of Ukraine, Kyiv 03022, Ukraine*

[3]*V.M. Glushkov Institute of Cybernetics, NAS of Ukraine, Kyiv 03187, Ukraine*

[4]*Institute of Biology and Medicine, Taras Shevchenko National University of Kyiv, Kyiv 01601, Ukraine*

[5]*Department of Biomedical Engineering, The Hong Kong Polytechnic University, Hong Kong SAR, China*

[*] Corresponding author's e-mail: iuliia@szu.edu.cn (I. Golovynska), lfr1993@163.com (F. Lin)

Methods

1. Next-generation whole-transcriptome RNA sequencing

RNA extraction. Mice were sacrificed 1 h after tPBMT by cervical dislocation to prevent chemical anesthesia from interfering with the aims of the research project. The skull was carefully opened, and the brain was extracted. After brain dissection on a chilled surface, the cortex and hippocampus tissues were isolated and immediately collected in tubes for flash freezing in nitrogen. Total RNA was isolated from each cortex and hippocampus sample of two groups (control and experiment) using an RNAmini kit (Qiagen, Germany). RNA quality was examined by gel electrophoresis and with Qubit (Thermo, Waltham, USA) (Fig. A and B).

RNA sequencing. RNA samples from the two mouse groups (control and light-treated) were separated into two independent pools, each comprised of three distinct samples of equal amounts. Strand-specific libraries were constructed using a TruSeq RNA sample preparation kit (Illumina, USA), and sequencing was carried out using an Illumina Novaseq 6000 instrument. The raw data was handled by Skewer, and data quality was checked by FastQC v0.11.2 (www.bioinformatics.babraham.ac.uk/projects/fastqc/). The read length was 2×150 bp. Clean reads were aligned to the Mus musculus mouse genome using STAR (Fig. C). The list of analyzed genes is in the end of this supplementary file.

Pathway and gene ontology analysis. The expression of gene transcript was calculated by fragments per kilobase of exon model per million mapped reads (FPKM) using Perl. The gene set enrichment analysis was performed for differentially expressed transcripts (DETs), which were determined using the MA-plot-based method with random sampling (MARS) model in the differentially expressed coding genes (DEG)seq package between two groups. The sequencing data were presented using the 2-fold units calculated from the gene expression data according to FPKM. Accordingly, DETs were calculated as a 2-fold change. The thresholds for determining DETs are $P < 0.05$ and absolute fold change ≥ 2. Then, DETs were chosen for molecular functions, biological processes, cellular components, and signaling pathway enrichment analysis using the Gene Ontology (GO) and Kyoto Encyclopedia of Genes and Genomes (KEGG) databases. Both differential gene and GO enrichment analyses were corrected using multiple hypothesis tests. GO enrichment analysis of background genes used genes with GO-annotated information. The pathway analysis was conducted via the Enrichr web gene list enrichment analysis tool (www.genome.jp/kegg). R software was further used to generate dot-plot graphs, using the ggplot2 library, and the GO chord graphs, using

the Goplot library. GO semantics classes and pathways were considered significant for $P < 0.05$. Analyses were carried out by using all DEGs (ALL DEGs) as well as the separated upregulated (ALL UP) and downregulated (ALL DOWN) DEGs of each brain region for two groups. Only a statistically significant KEGG pathway and GO terms were taken into consideration for further analysis and interpretation.

Differential expression gene screening and statistics. For experimental design, DESeq2 software was used to screen differentially expressed genes between different sample groups to meet the requirements of $|log2FC| \geq 1$ and P-value < 0.05. The sorting of genes was performed using a homemade software protocol.

2. Western Blot analysis

Western blot (WB) analysis was performed to determine proteins in the mouse brain. The total protein concentration of each sample was measured by the BCA protein assay kit (Thermo Fisher Scientific, USA). To block the non-specific binding of the antibodies to the polyvinylidene difluoride membrane, the samples were placed in a diluted protein solution with 4% bovine serum albumin (Thermo Fisher Scientific, USA) for 1.5 h at room temperature. Then, samples were bound to the monoclonal antibodies (1:500-1:2000, Thermo Fisher Scientific, USA) for 2 h (Table S1). An enhanced chemiluminescence kit (Thermo Fisher Scientific, USA) was used to detect immunoreactive bands for 1 h. The bands were scanned and digitalized, and the density of each band was quantified using FIJI software and normalized to β-actin. The original uncropped and unadjusted images underlying of the WB results (Fig. 6 of the main text) are presented in Fig. D.

Table A. The list of antibodies for Western blot analysis

Antibody	Host/Isotype	Dilution	Manufacturer
PKC alpha Monoclonal Antibody	Rabbit / IgG	1:1000	Thermo Fisher Scientific
c-Jun Monoclonal Antibody	Rabbit / IgG	1:1500	Thermo Fisher Scientific
JunD Recombinant Rabbit Monoclonal Antibody	Rabbit / IgG	1:1000	Thermo Fisher Scientific
TNF alpha Monoclonal Antibody	Rabbit / IgG	1:1000	Thermo Fisher Scientific
IL-1 beta Monoclonal Antibody	Rabbit / IgG	1:1000	Thermo Fisher Scientific
IL-17A Monoclonal Antibody	Rabbit / IgG	1:1000	Thermo Fisher Scientific
MANF Monoclonal Antibody	Rat / IgG	1:1500	Thermo Fisher Scientific
Caspase-3 Recombinant Monoclonal Antibody	Rabbit / IgG	1:1000	Thermo Fisher Scientific
Amyloid Precursor Protein Monoclonal Antibody	Rat / IgG	1:1000	Thermo Fisher Scientific
BACE1 Recombinant Monoclonal Antibody	Rabbit / IgG	1:1500	Thermo Fisher Scientific
BACE2 Recombinant Monoclonal Antibody	Rabbit / IgG	1:1500	Thermo Fisher Scientific
Presenilin 1 Monoclonal Antibody	Goat / IgG	1:500	Thermo Fisher Scientific
Presenilin 2 Monoclonal Antibody	Goat / IgG	1:500	Thermo Fisher Scientific
APH1B Polyclonal Antibody	Goat / IgG	1:2000	Thermo Fisher Scientific
Actin Monoclonal Antibody	Rabbit / IgG	1:1000	Thermo Fisher Scientific

Results

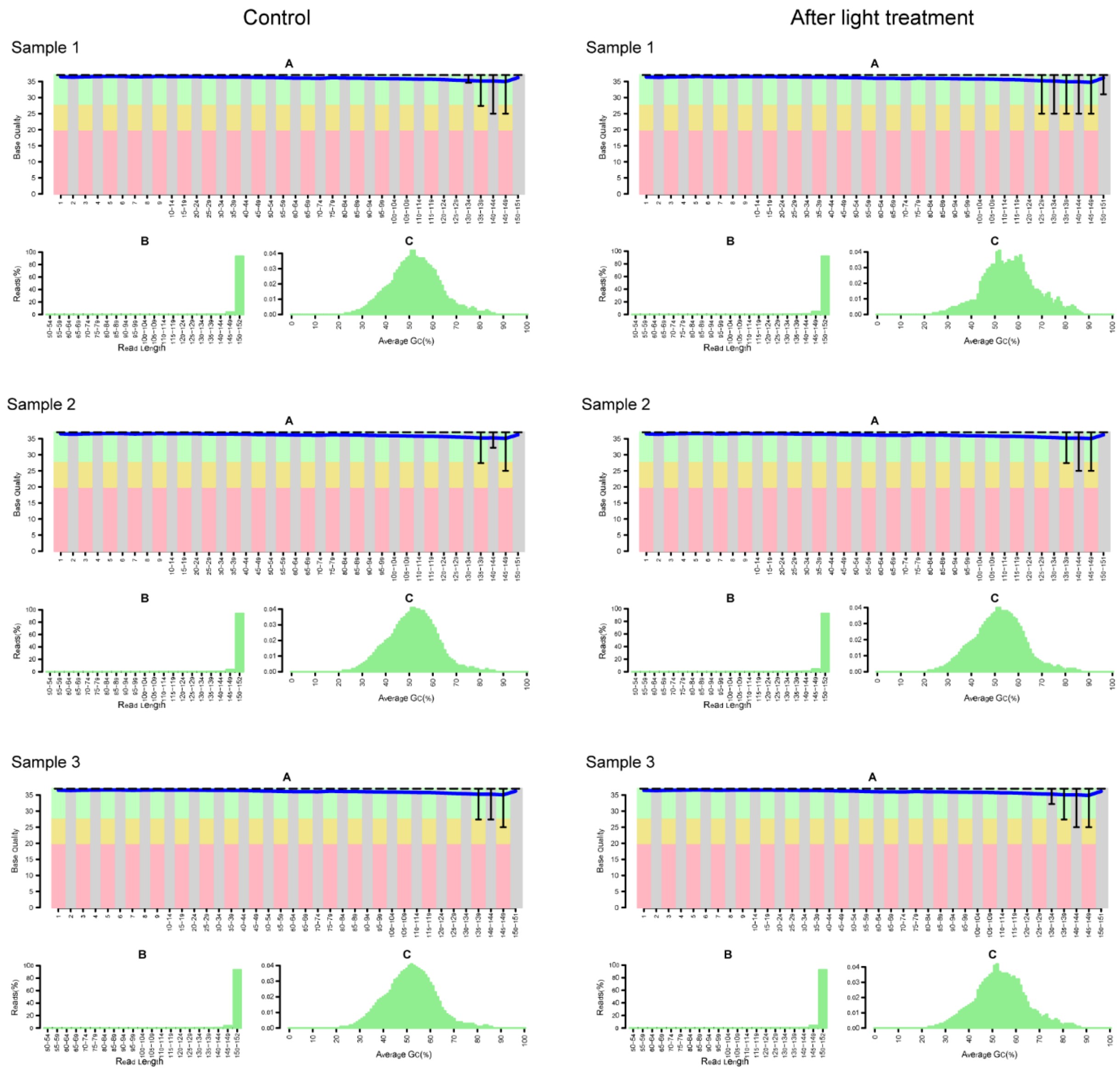

Figure A. Sample quality analysis of the control cortex area and after light therapy, using the GC content distribution: **(A)** The mass score distribution diagram of each base position of the sequence (the abscissa - the base position of the sequence and the ordinate - the mass score distribution of the base position). **(B)** Sequence length distribution diagram (the abscissa - the sequence length and the ordinate - the ratio of the number of sequences). **(C)** GC percentage sequence number density distribution map (the abscissa - the GC percentage and the ordinate - the sequence number ratio).

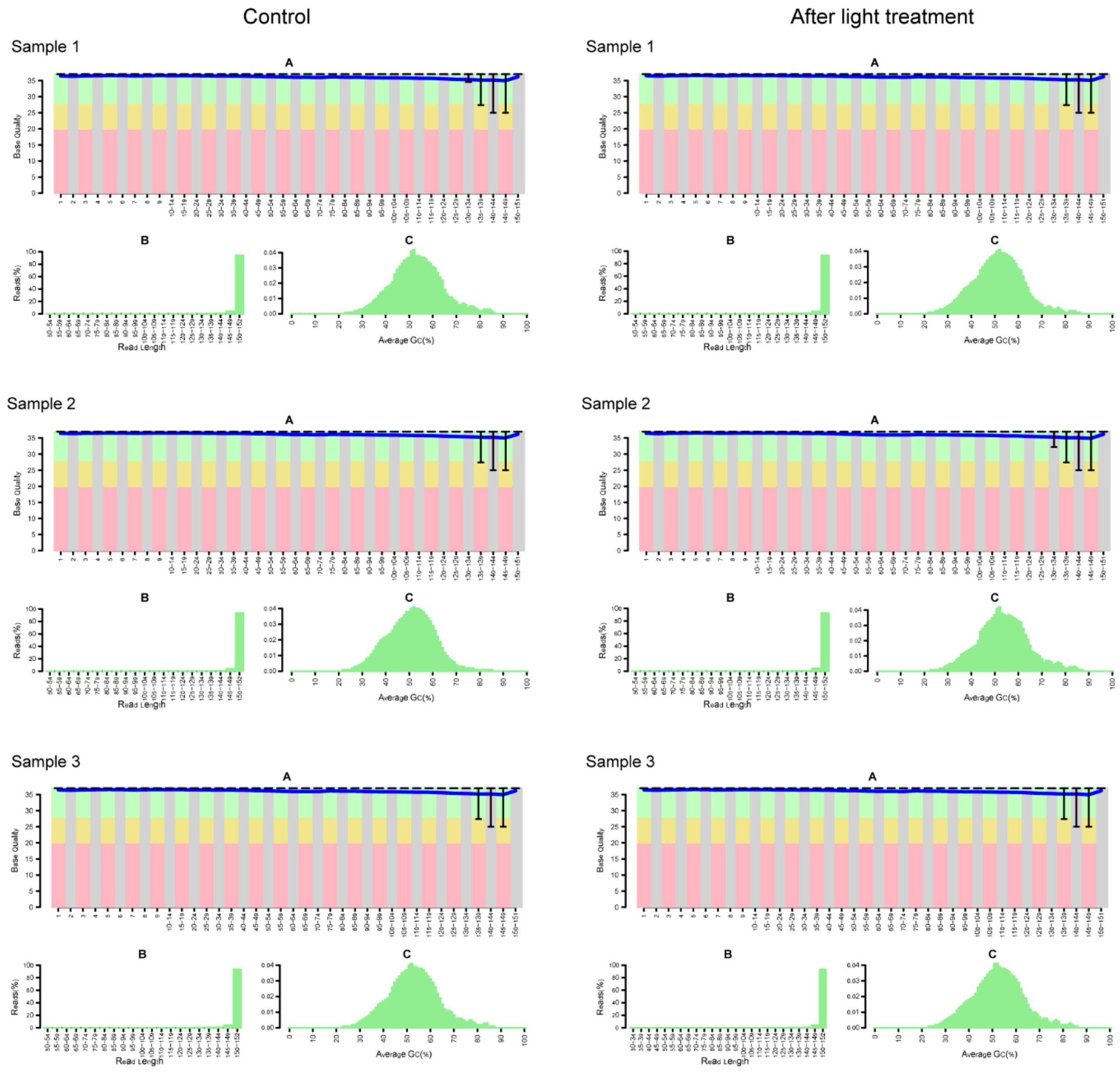

Figure B. Sample quality analysis of the control hippocampus area and after light therapy, using the GC content distribution: **(A)** The mass score distribution diagram of each base position of the sequence (the abscissa - the base position of the sequence and the ordinate - the mass score distribution of the base position). **(B)** Sequence length distribution diagram (the abscissa - the sequence length and the ordinate - the ratio of the number of sequences). **(C)** GC percentage sequence number density distribution map (the abscissa - the GC percentage and the ordinate - the sequence number ratio).

Control

Sample 1

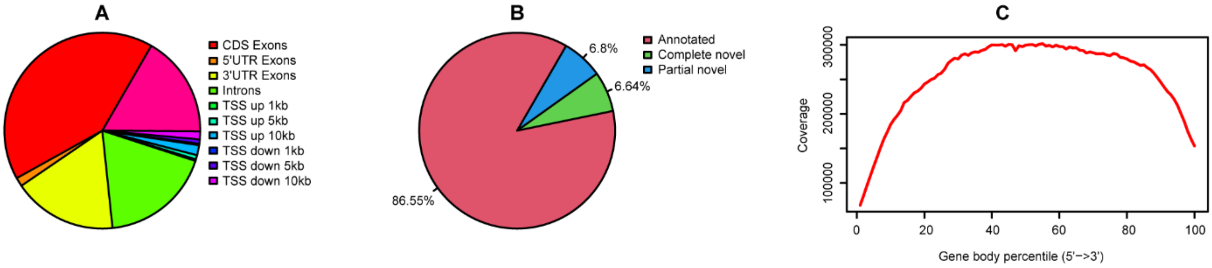

Sample 2

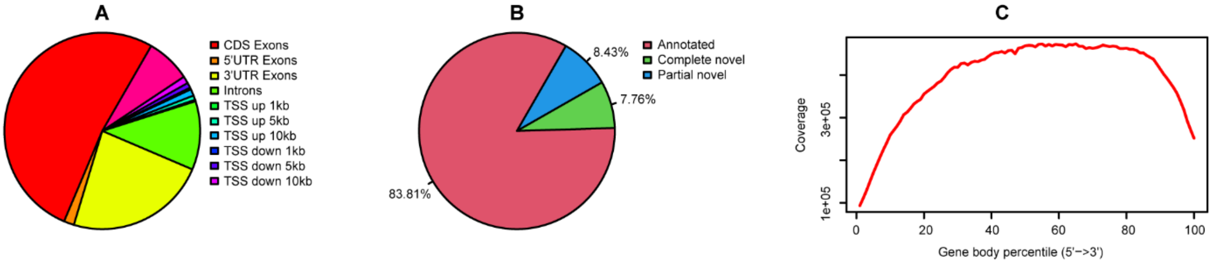

Sample 3

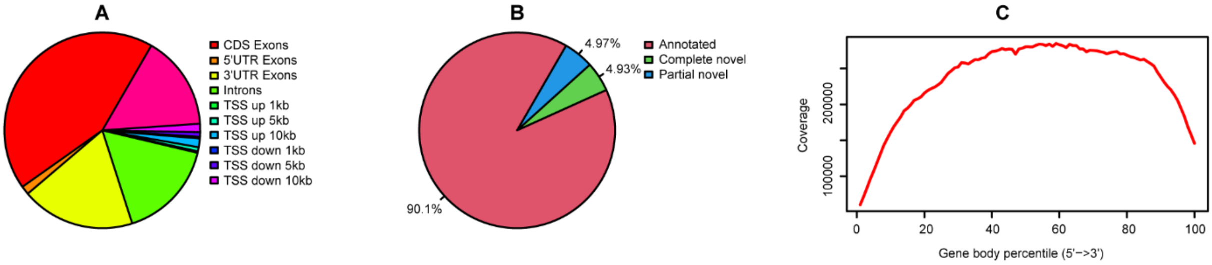

Experiment

Sample 1

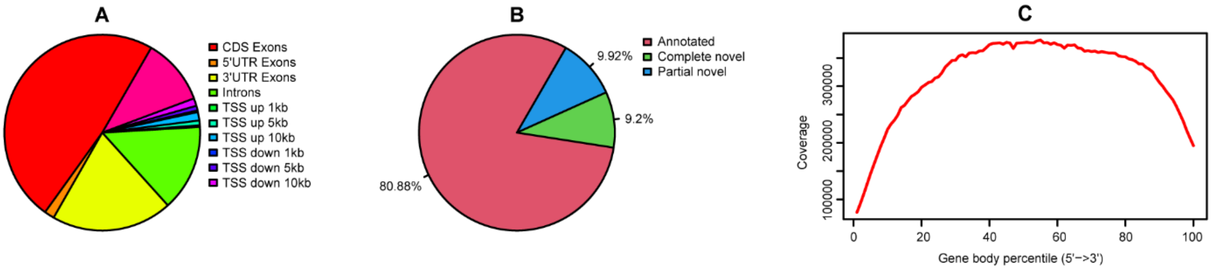

Sample 2

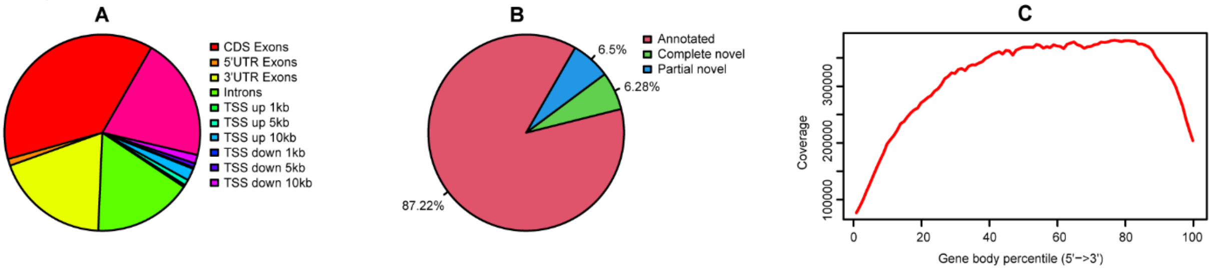

Sample 3

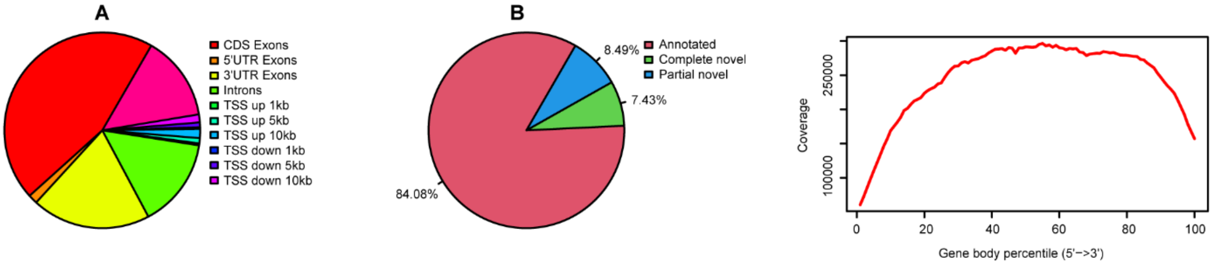

Figure C. ***Reads*** **compared to the results of the reference genome, the number of sequences of each chromosome is counted, and then the average depth is calculated within every 5kb of the reference genome, and log2 is taken to complete the statistics of the density distribution of the reference genome used to detect abnormalities in the distribution of sequencing sequences on the reference genome.**

(A) Pie chart of the distribution of *reads* comparison exons, introns, and gene interval regions. Detailed description: The calculated coverage of *reads* in different gene components for comparison of the reference genome (such as CDS exon, 5'UTR exon, 3'UTR exon, 3 'UTR exon, Intron), *reads* alignment distribution evaluation statistics the number of unique *reads* aligned to each region, that is if the same *reads* align to the same region, but the region may be annotated as exons and introns at the same time (two different transcript elements), the number of alignments of the *reads* in the priority genomic region is only recorded once according to a certain priority order, and the priority order of the region count is CDS exons > UTR exons > Introns. For example, if a read is mapped to a region belonging to CDS exon and intron, it will be marked as belonging to CDS exons.

(B) Splice site annotation statistical pie chart. Detailed description: The evaluation analysis process of variable splice site annotation is based on comparing the reference genome and known gene model annotation information of each sample, and comparing the known splice junction (cut site) to obtain the number and ratio of new splice junctions (cutting sites) in the current transcriptome. Each detected splice junction can be divided into 3 independent types: (1) annotated - all belong to the splice sites in the known gene model annotations, that is, including the 5-terminal splice site of the splice site and the 3 terminal cleavage site; (2) complete novel - all belong to new splicing sites, and neither end of the splicing site belongs to the annotated part in the known gene model; (3) partial novel - a certain part (5'SS or 3'SS) belongs to the cut site in the known gene model annotation, and another part (3'SS or 5'SS) belongs to the new cut site.

(C) Transcript coverage uniformity distribution map (the abscissa - the position of 100nt after normalization of the transcript length and the ordinate - the number of *reads* covered in each sub-region position). Detailed description: transcript coverage uniformity distribution evaluation. It is used to detect whether the sequencing *reads* within the transcript are uniform and whether there is a 5'/3' bias. The evaluation method is: during the analysis, all known transcripts are normalized to a region with a length of 100nt, and the coverage is calculated in each number of *reads* at the region location. Finally, a *reads* coverage distribution map describing the 5'/3' direction of the gene body is provided. There is no 5/3 deviation in the correct distribution state. The quality of the RNA-Seq sequencing experiment is very good and can be used for further data analysis.

Cortex, biological process

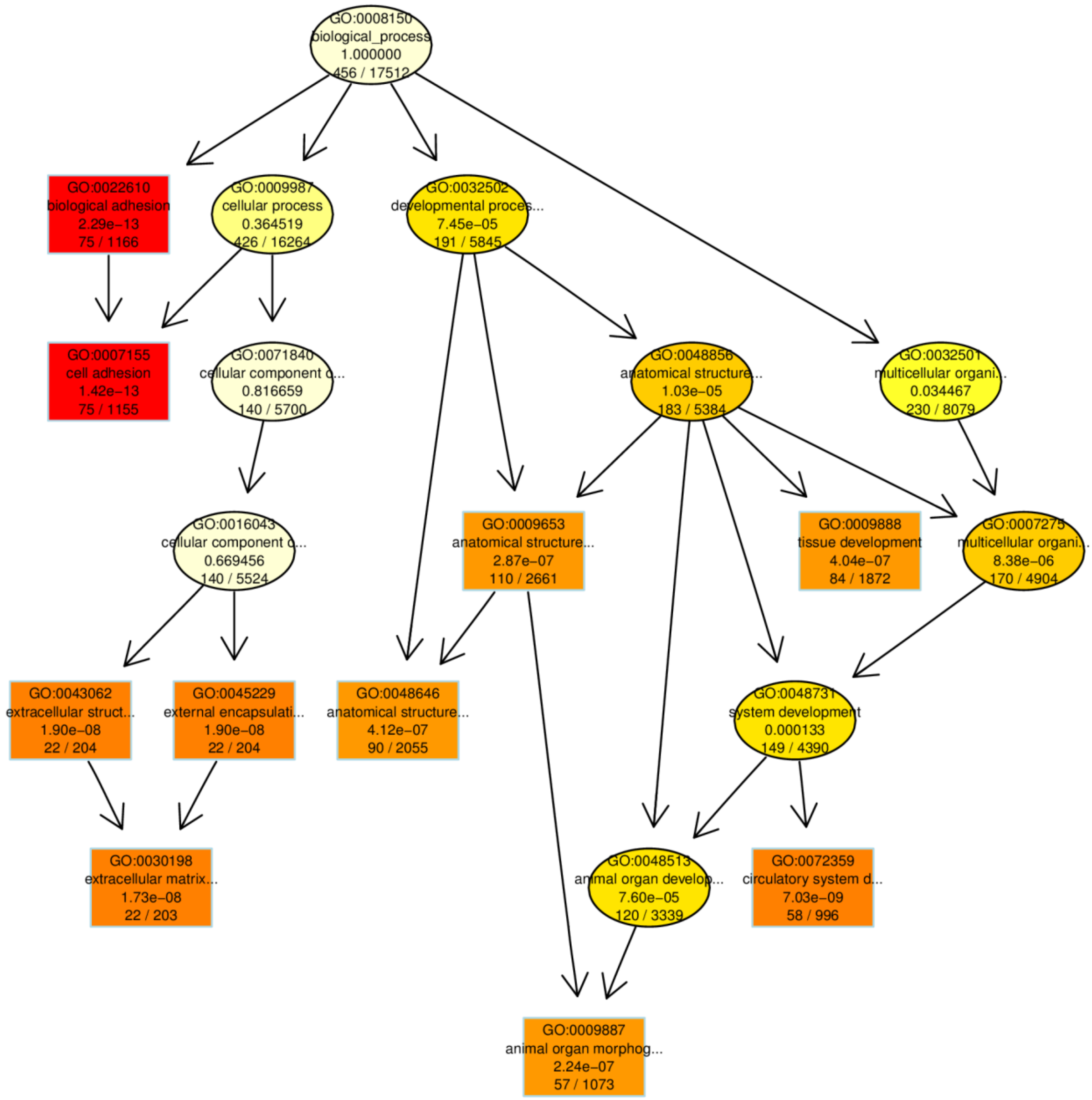

Fig. D. Significant enrichment tree chart of genes modulated by 808 nm light of cortex for gene ontology (GO) category of biological process, showing the top 10 GOs with significant enrichment (in the boxes), where each node represents a GO, the color depth indicates the degree of enrichment - the darker the color, the higher the degree of enrichment, and a GO name and *p*-value are displayed in each node. The GO details are available in https://www.informatics.jax.org/vocab/gene_ontology/.

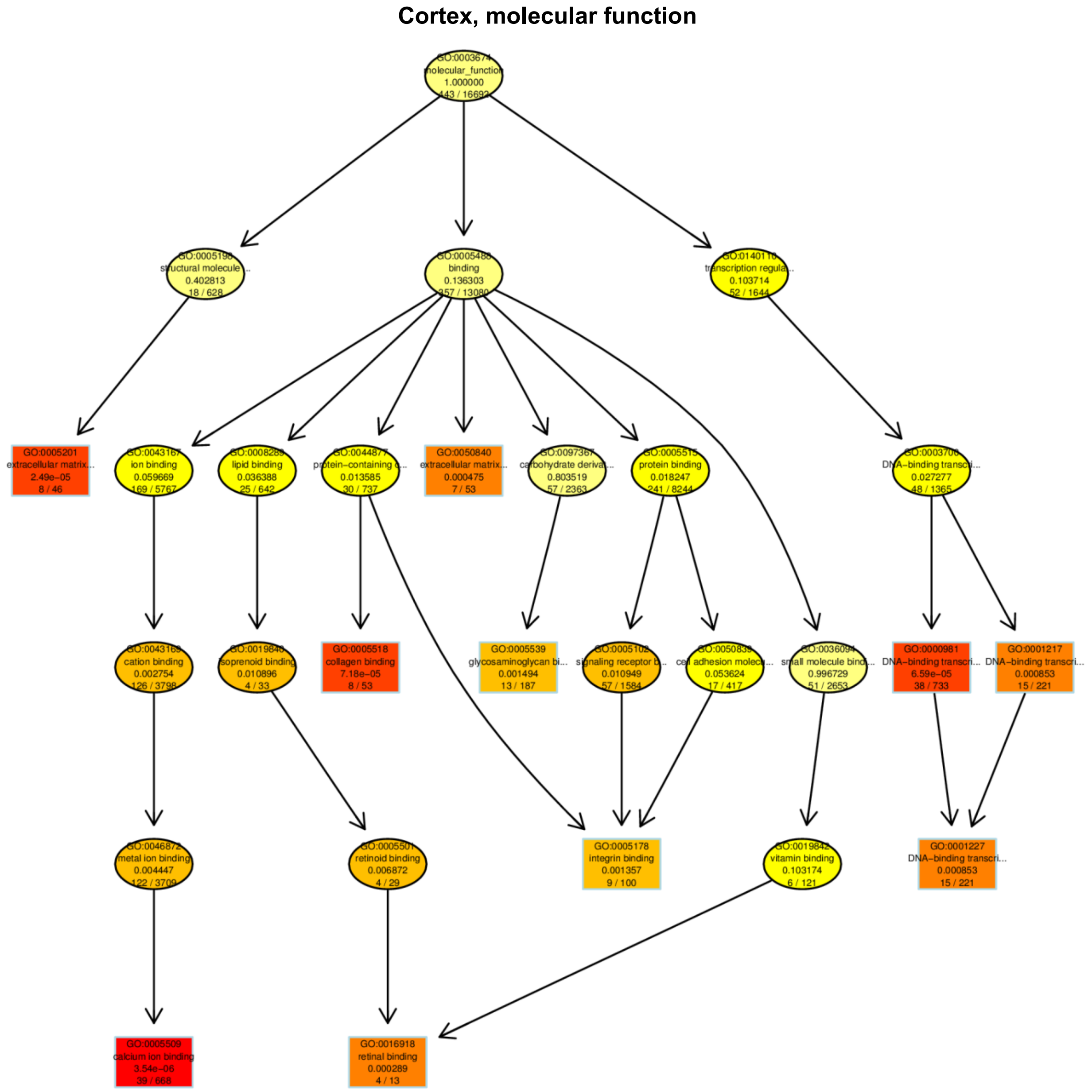

Fig. E. Significant enrichment tree chart of genes modulated by 808 nm light of cortex for gene ontology (GO) category of molecular function, showing the top 10 GOs with significant enrichment (in the boxes), where each node represents a GO, the color depth indicates the degree of enrichment - the darker the color, the higher the degree of enrichment, and a GO name and *p*-value are displayed in each node. The GO details are available in https://www.informatics.jax.org/vocab/gene_ontology/.

Investigating molecular function, we attempted to identify potential transcription factors (TFs), signal transducers, and activators of transcription that regulate differentially expressed metabolic genes. We performed the enrichment analysis to identify differentially expressed metabolic genes in comparison to the control untreated mice and those after the tPBMT course. They are 23 TFs identified in the cortex: activating TF 6 (ATF6), basic leucine zipper TF ATF-like 3 (BATF3), basic TF 3 (BTF3), general TF IIA 1-like (GTF2A1L), GTF2E polypeptide 1 α-subunit (GTF2E1), GTF2 I repeat domain-containing 1 (GTF2IRD1), MYC-associated zinc finger protein – a purine-binding TF (MAZ), NK-3 TF locus 1 Drosophila (NKX3.1), runt related TF 2 opposite strand 1 (RUNX2OS1), runt related TF 2 opposite strand 3 (RUNX2OS3), Sp7 TF 7 (SP7), trans-acting TF 8 (SP8), TF 15 (TCF15), TCF24, TF AP-2 α (TFAP2a), upstream TF 1 (USF1), yin yang 2 TF (YY2), GATA binding protein 2 (GATA2), v-rel reticuloendotheliosis viral oncogene homolog A - avian (RELA), signal transducer and activator of transcription 3 (STAT3), Jun proto-oncogene (JUN), aryl hydrocarbon receptor nuclear translocator-like (ARNTL), and cAMP responsive element binding protein 3-like 1 (CREB3L1).

Cortex, cellular component

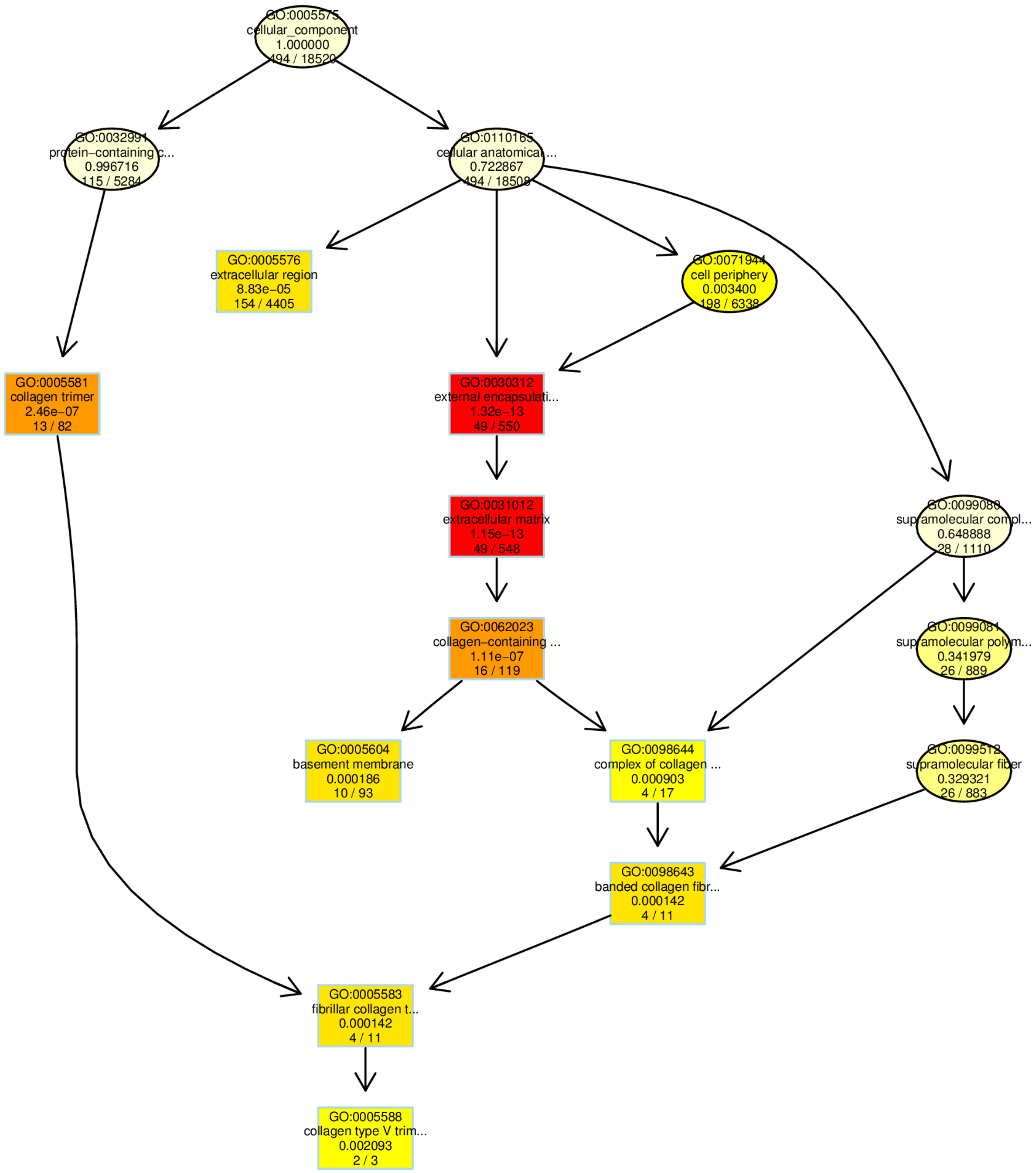

Fig. F. Significant enrichment tree chart of genes modulated by 808 nm light of cortex for gene ontology (GO) category of cellular component, showing the top 10 GOs with significant enrichment (in the boxes), where each node represents a GO, the color depth indicates the degree of enrichment - the darker the color, the higher the degree of enrichment, and a GO name and *p*-value are displayed in each node. The GO details are available in https://www.informatics.jax.org/vocab/gene_ontology/.

Hippocampus, biological process

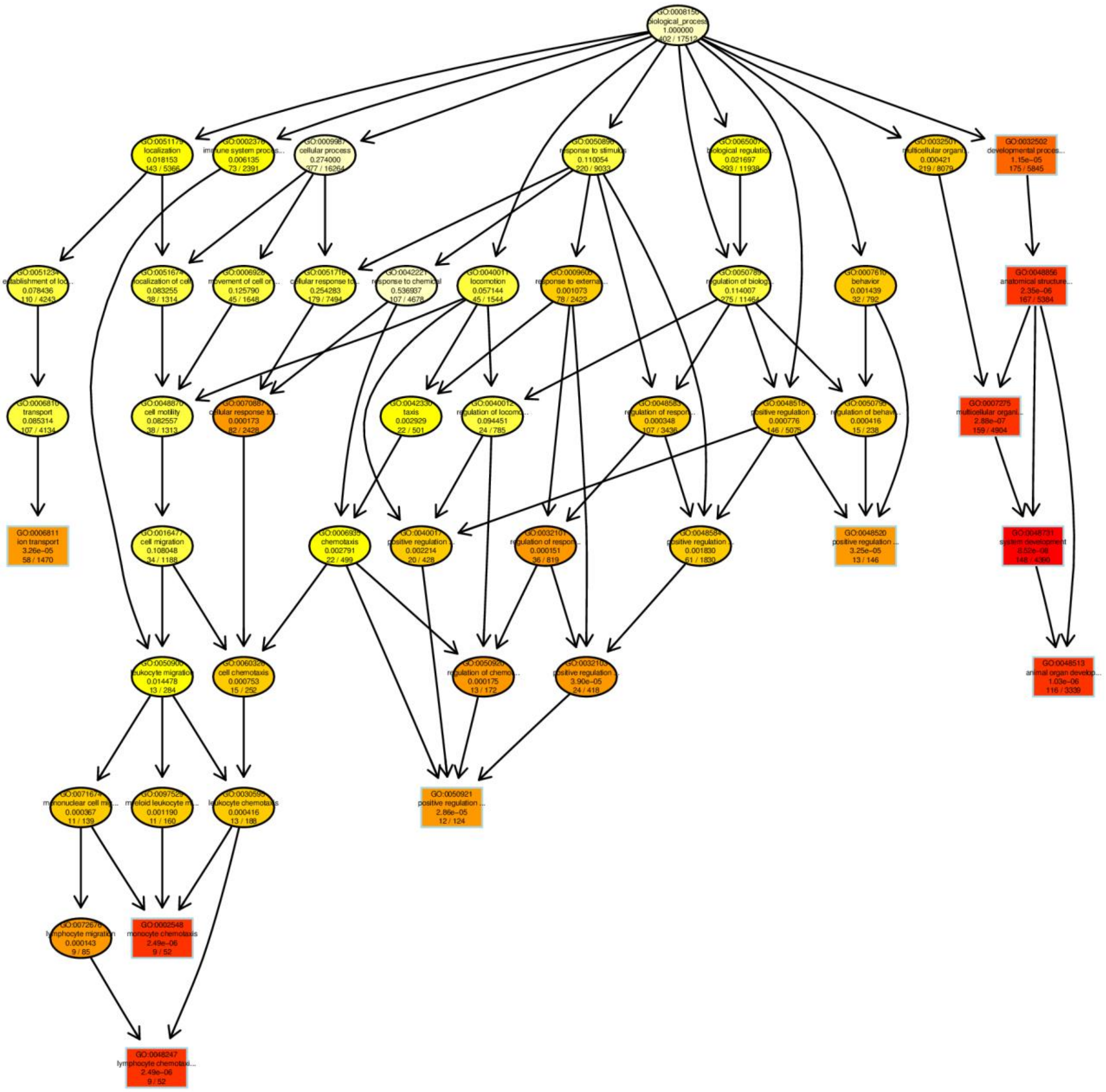

Fig. G. Significant enrichment tree chart of genes modulated by light of hippocampus for gene ontology (GO) category of biological process, showing the top 10 GOs with significant enrichment (in the boxes), where each node represents a GO, the color depth indicates the degree of enrichment - the darker the color, the higher the degree of enrichment, and a GO name and *p*-value are displayed in each node. The GO details are available in https://www.informatics.jax.org/vocab/gene_ontology/.

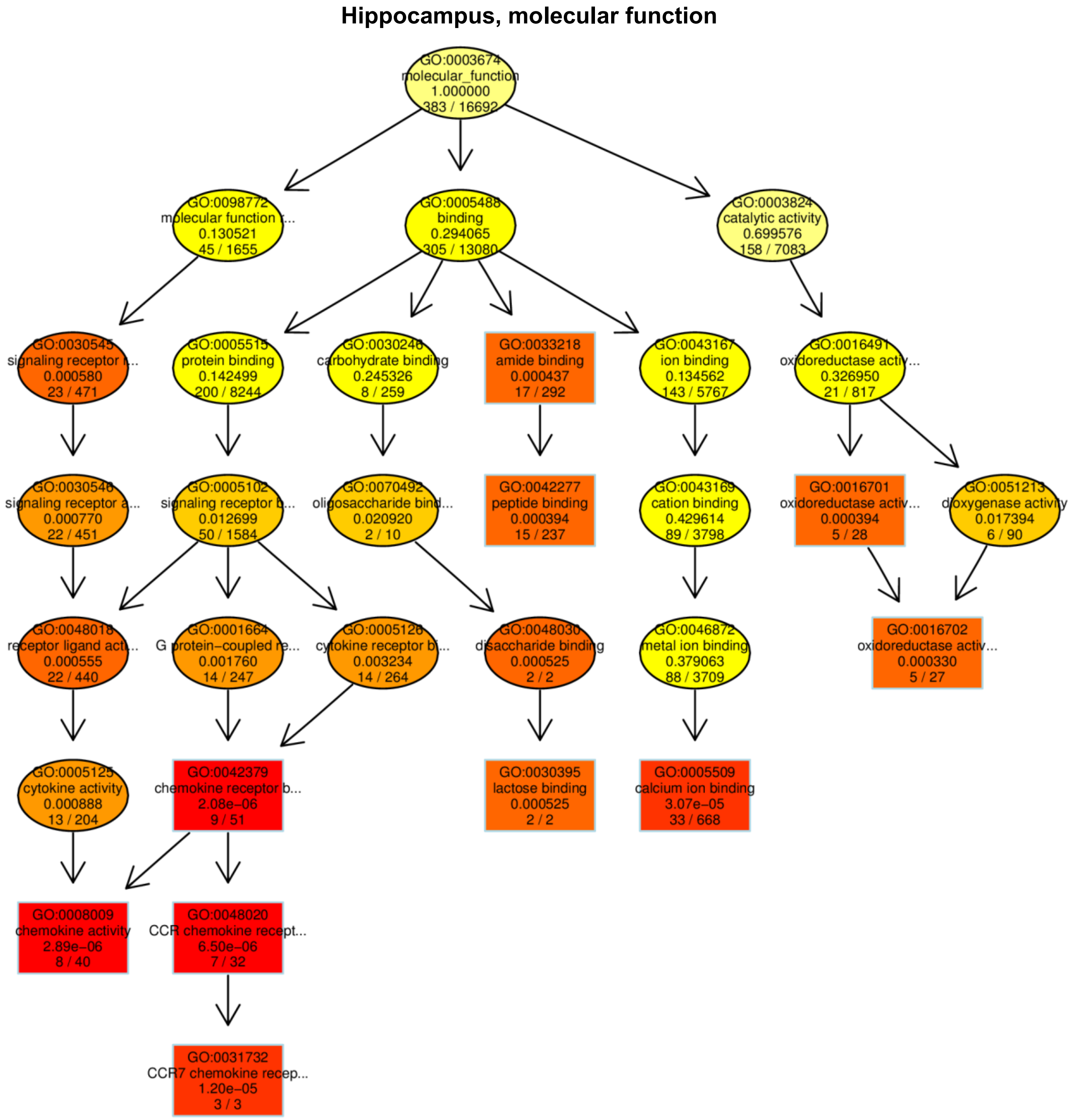

Fig. H. Significant enrichment tree chart of genes modulated by 808 nm light of hippocampus for gene ontology (GO) category of molecular function, showing the top 10 GOs with significant enrichment (in the boxes), where each node represents a GO, the color depth indicates the degree of enrichment - the darker the color, the higher the degree of enrichment, and a GO name and *p*-value are displayed in each node. The details are available in https://www.informatics.jax.org/vocab/gene_ontology/.

Investigating molecular function, we attempted to identify potential transcription factors (TFs), signal transducers, and activators of transcription that regulate differentially expressed metabolic genes. We performed the enrichment analysis to identify differentially expressed metabolic genes in comparison to the control untreated mice and those after the tPBMT course. They are 16 TFs identified in the hippocampus: BATF, E2F TF 4 (E2F4), GTF2A1, GTF2A1L, GTF3C4, GTF3C5, oligodendrocyte TF 2 (OLIG2), pre B cell leukemia TF interacting protein 1 (PBXIP1), POU domain class 3 TF 4 (POU3F4), TF Dp 2 (TFDP2), upstream TF 2 pseudogene 1 (USF2-PS1), CUGBP Elav-like family member 2 (CELF2), GATA2, ETS TF ELK3 (ELK3), CCAAT/enhancer binding protein Δ (CEBPD), and JUND.

Hippocampus, cellular component

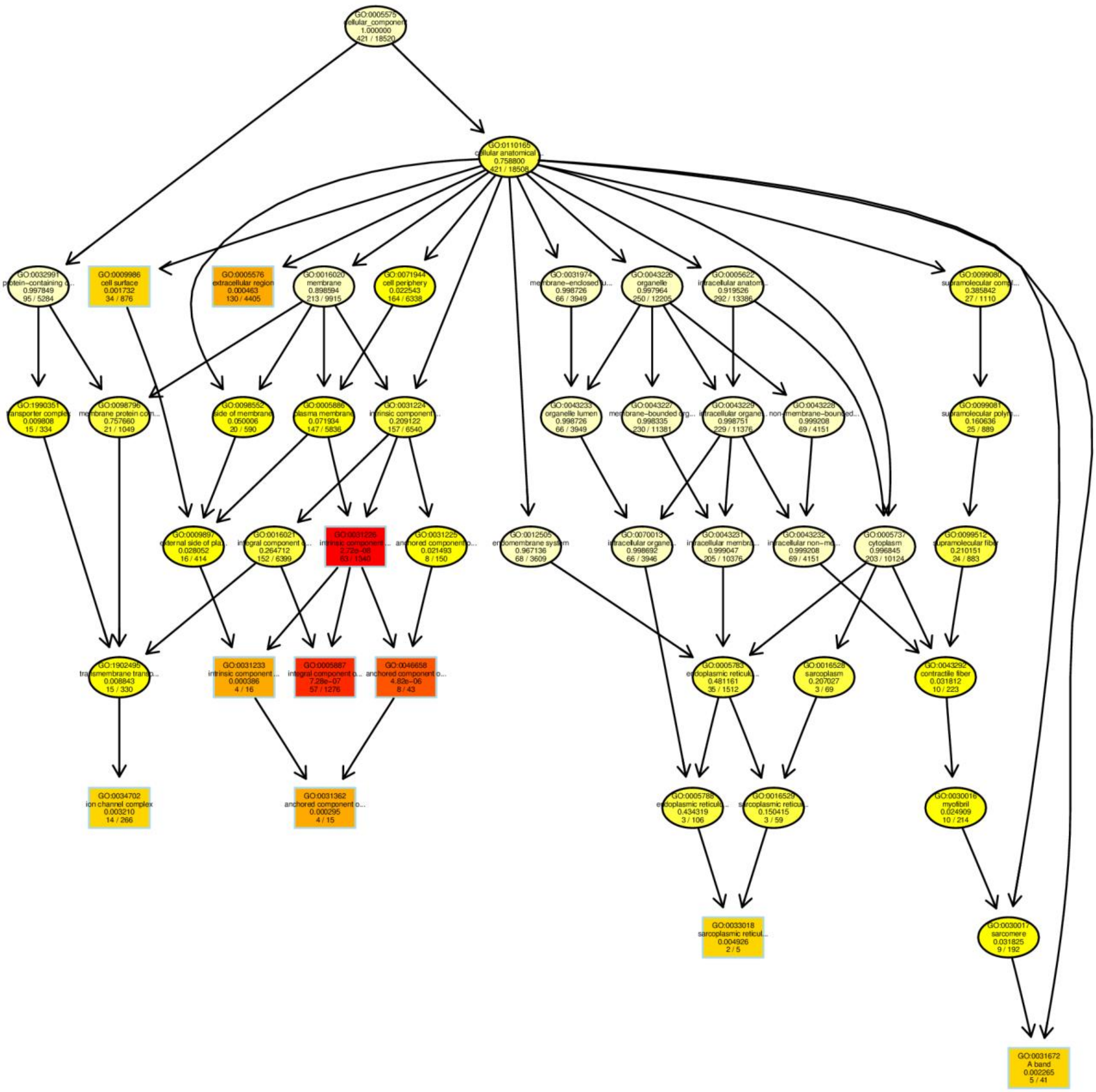

Fig. I. Significant enrichment tree chart of genes modulated by 808 nm light of hippocampus for gene ontology (GO) category of cellular component, showing the top 10 GOs with significant enrichment (in the boxes), where each node represents a GO, the color depth indicates the degree of enrichment - the darker the color, the higher the degree of enrichment, and a GO name and *p*-value are displayed in each node. The details are available in https://www.informatics.jax.org/vocab/gene_ontology/.

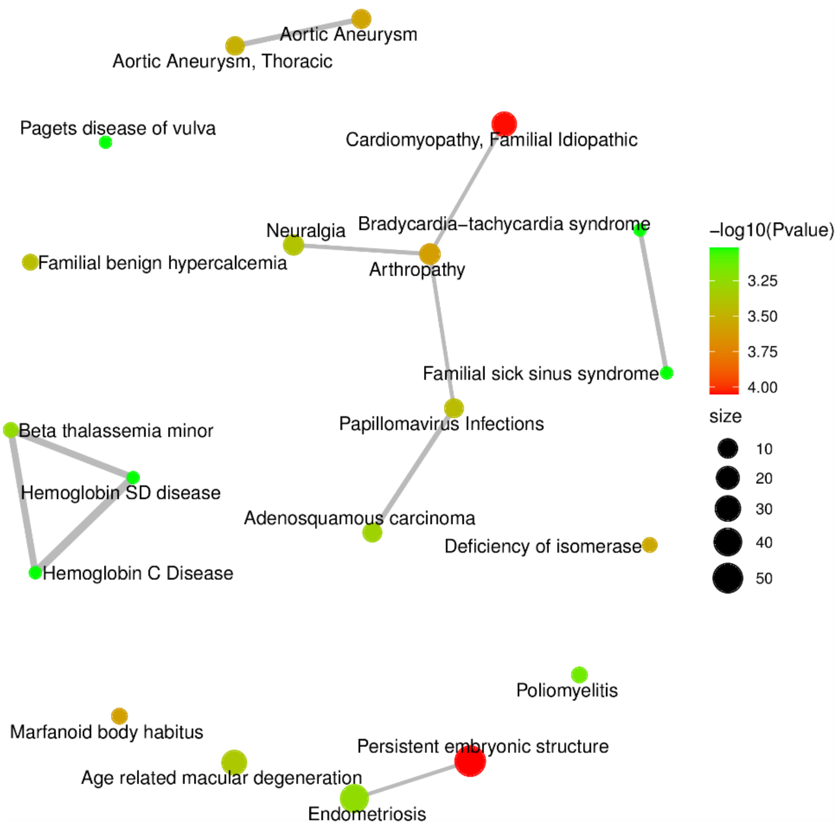

Fig. J. Network diagram of the first 20 diseases-diseases for the cortex area modulated by 808 nm light, according to the sorting information of –log10 (p-value), where the ratio of the number of overlapping genes to the number of unique genes of the two is ≥ 20%, the node size indicates the total number of candidate genes belonging to disease, and the color indicates –log10 (p-value).

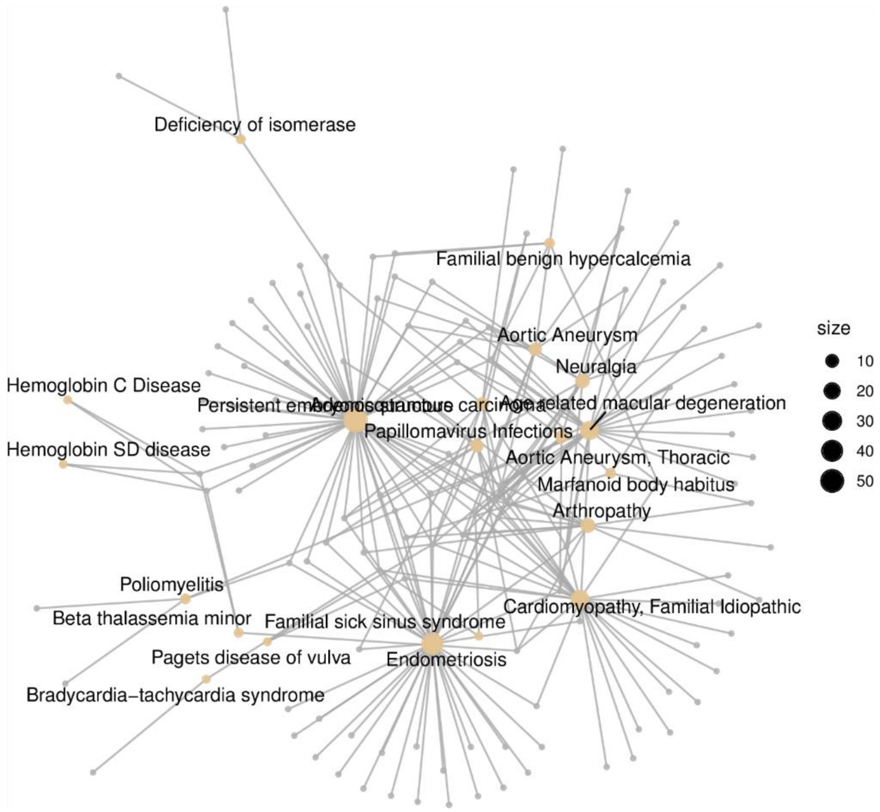

Fig. K. Network diagram of the top 20 diseases and candidate genes for the cortex area modulated by 808 nm light, according to the sorting information of –log10 (p-value), where the node size indicates the total number of candidate genes belonging to disease.

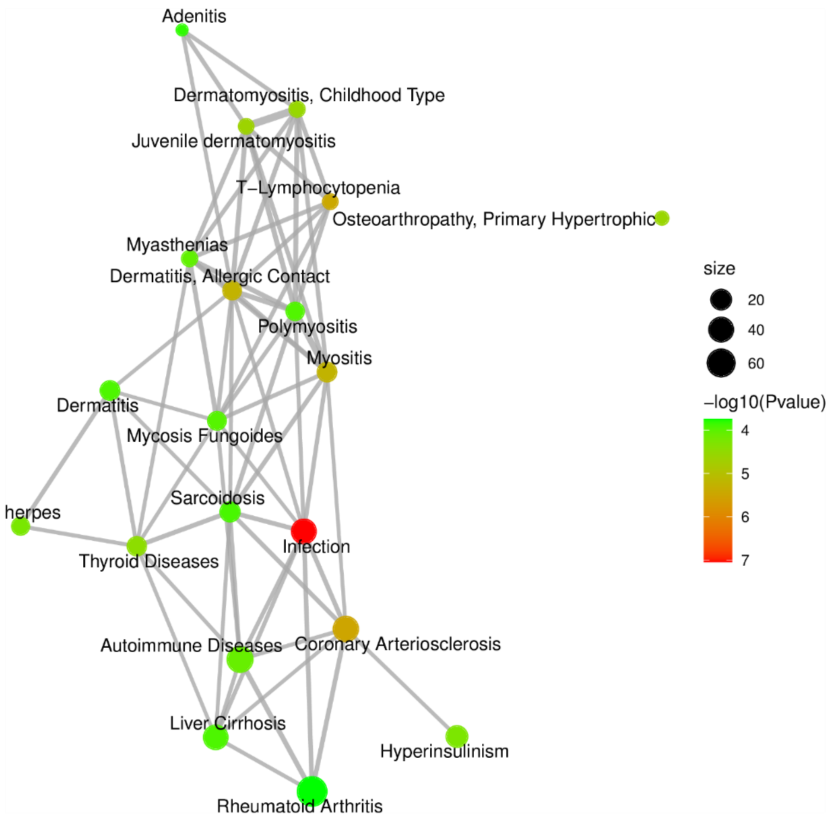

Fig. L. Network diagram of the first 20 diseases-diseases for the hippocampus area modulated by 808 nm light, according to the sorting information of –log10 (p-value), where the ratio of the number of overlapping genes to the number of unique genes of the two is ≥ 20%, the node size indicates the total number of candidate genes belonging to disease, and the color indicates –log10 (p-value).

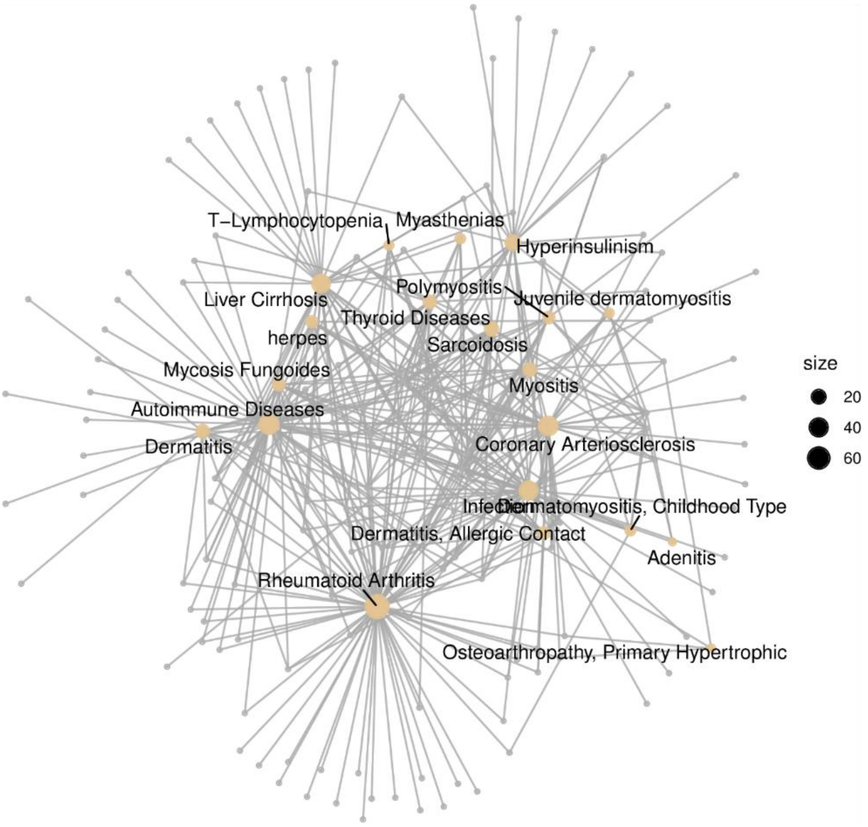

Fig. M. Network diagram of the top 20 diseases and candidate genes for the hippocampus area modulated by 808 nm light, according to the sorting information of –log10 (p-value), where the node size indicates the total number of candidate genes belonging to disease.

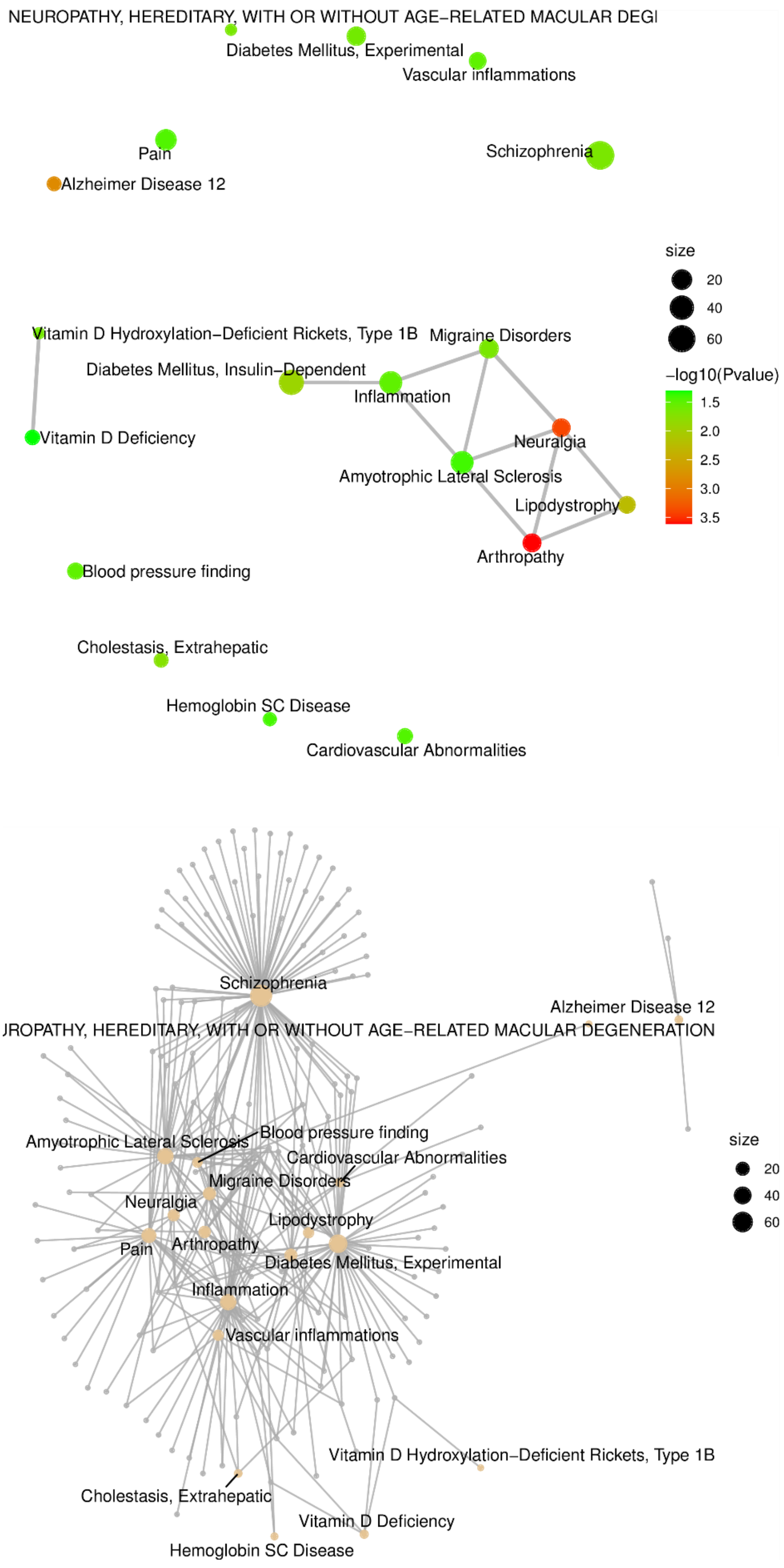

Fig. N. Network diagrams for the cortex modulated by 808 nm light, according to the sorting data of –log10 (*P*-value). (Up) Disease network diagram, where the ratio of the number of overlapping genes to the number of unique genes of the two is ≥ 20%, the node size indicates the total number of candidate genes belonging to the disease, and the color indicates –log10 (*P*-value). **(Down)** Network diagram of the disease and candidate genes, where the node size indicates the total number of candidate genes belonging to the disease.

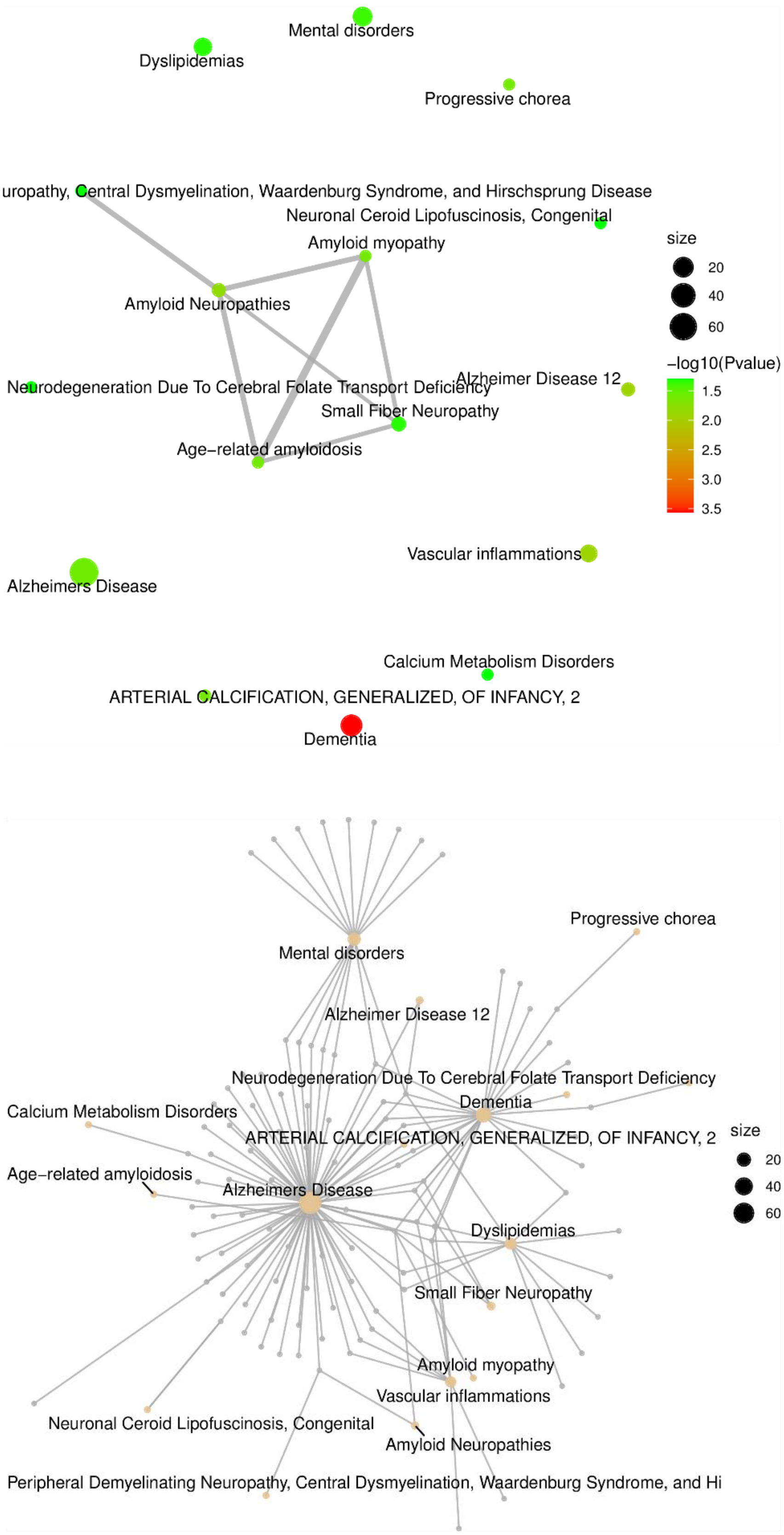

Fig. O. Network diagrams for the hippocampus modulated by 808 nm light, according to the sorting data of –log10 (*P*-value). (Up) Disease network diagram, where the ratio of the number of overlapping genes to the number of unique genes of the two is ≥ 20%, the node size indicates the total number of candidate genes belonging to the disease, and the color indicates –log10 (*P*-value). **(Down)** Network diagram of the disease and candidate genes, where the node size indicates the total number of candidate genes belonging to the disease.

Table B. Statistically significant enriched gene expression of Kyoto Encyclopedia-defined Genes and Genomes (KEGGs) involved in metabolic pathways modulated by 808 nm tPBMT for the cortex (C) and hippocampus (H).

Apoptosis

Symbol	C	H
Airn		
Bcl2l15		
Bmf		
Btg3		
Casp3		
Cav1		
Ccna1		
Ccnd1		
Cdkn1a		
Crebbp		
Ctnnb1		
Cyld		
Dcn		
Erbb2		
Gpx3		
Gsn		
Jun		
Mcl1		
Pdgfrb		
Plppr4		
Psen2		
Retsat		
Sat1		
Satb1		
Tgfb2		
Timp2		
Txnip		
Wee1		

Glycolysis

Symbol	C	H
9530052E02Rik		
Aldh9a1		
Artn		
Dcn		
Ext1		
Gm16379		
Gm8566		
Homer1		
Ndst3		
Omp		
Pfkfb1		
Plod2		
Pygl		
Ssr2		
Stc2		
Zfp292		

E2F targets

Symbol	C	H
Bard1		
Cdk4		
Cdkn1a		
Cenpe		
Hmgb3		
Mad2l1		
Mcm6		
Mre11a		
Pop7		
Psmc3ip		
Rrm2		
Shmt1		
Srsf2		
Tbrg4		
Tfrc		
Tipin		
Ube2s		
Wee1		

Cholesterol homeostasis

Symbol	C	H
Acat2		
Antxr2		
Atxn2		
Cpeb2		
Ctnnb1		
Dhcr7		
Errfi1		
Fasn		
Fdps		
Hmgcr		
Hmgcs1		
Idi1		
Jag1		
Ldlr		
Lpl		
Lss		
Mvd		
Mvk		
Nfil3		
S100a11		
Scd1		
Sqle		
Stard4		
Stx5a		

Fatty acid metabolism

Symbol	C	H
Acat2		
Alad		
Aldh9a1		
Elovl5		
Hmgcl		
Hmgcs1		
Hmgcs2		
Hpgd		
Hsd17b4		
Hsdl2		
Idh3g		
Idi1		
Mgll		
Prdx6		
Retsat		
Sco2		
Tdo2		

Oxidative phosphorylation

Symbol	C	H
Acadsb		
Atp6v1f		
Bcat2		
Cox15		
Gm7180		
Hadhb		
Idh1		
Idh3g		
Immt		
Mdh1		
Mrps11		
Ndufb7		
Oat		
Pdk4		
Pdp1		
Retsat		
Rhot1		

MYC targets V1

Symbol	C	H
AC117232.1		
2310002F09Rik		
4933406I18Rik		
Apex1		
Cdk4		
Ctps		
Eif4e		
Gm16546		
Gm8173		
Hddc2		
Hnrnpr		
Mad2l1		
Mcm6		
Myc		
Npm1		
Ppia		
Psmb2		
Rps10		
Srsf2		
Tyms		

MYC targets V2

Symbol	C	H
Gm26316		
Cdk4		
Npm1		
Tbrg4		

Angiogenesis

Symbol	C	H
Tnfrsf21		
Col3a1		
Jag1		
Lpl		
S100a3		
Slco2a1		
Spp1		
Vtn		

Pancreas β cells

Symbol	C	H
Akt3		
Chga		
Lmo2		
Nkx2-2		
Pak3		
Pax6		
Scgn		
Vdr		

HEME metabolism

Symbol	C	H
Alad		
Bcam		
E2f2		
Epb41		
Lmo2		
Mboat2		
Nek7		
Rbm5		
Slc6a9		
Snca		
Tfdp2		
Tfrc		
Ucp2		

DNA repair

Symbol	C	H
AC122379.2		
Ada		
Bcam		
Cda		
Cetn2		
Ercc4		
Gm10143		
Gm17954		
Gtf3c5		
Lig1		
Nme3		
Polr1d		
Polr2k-ps		
Rfc5		
Smad5		
Snapc4		
Tk2		
Tmed2		
Tyms		

TNF-α signaling via NFKB

Symbol	C	H
9530052E02Rik		
Bcl6		
Bhlhe40		
Btg1		
Ccnd1		
Cdkn1a		
Cebpd		
Dram1		
Gem		
Gpsm1		
Jag1		
Jun		
Kdm6b		
Ldlr		
Mt2		
Nfil3		
Nfkbia		
Pfkfb3		
Ptgs2		
Sat1		
Sdc4		
Sgk1		
Smad3		
Tnfaip6		
Traf1		
Trib1		
Tsc22d1		

PI3K/AKT/MTOR signaling

Symbol	C	H
Calr		
Cdk4		
Cdkn1a		
Nfkbib		
Nod1		
Plcb1		
Ppp2r1b		
Prkaa2		
Prkcb		
Raf1		
Rps6ka3		
Smad2		
Tcp11l1		

Hypoxia

Symbol	C	H
9530052E02Rik		
Bhlhe40		
Brs3		
Btg1		
Car12		
Cav1		
Cavin1		
Cdkn1a		
Chst3		
Crmp1		
Csrp2		
Dcn		
Ddit4		
Errfi1		
Ets1		
Ext1		
Fbp1		
Fos		
Gaa		
Gm16379		
Hs3st1		
Hspa5		
Itgb4		
Jun		
Klf6		
Large1		
Lox		
Map3k1		
Mxi1		
Nagk		
Nfil3		
Nr3c1		
Pam		
Pfkfb3		
Pgf		
Ppp1r3c		
Prkca		
Rora		
Rragd		
S100a3		
Sdc4		
Selenbp2		
Slc25a1		
Slc6a6		
Srpx		
Tmem45a		
Wsb1		
Zfp292		

Coagulation

Symbol	C	H
Comp		
Ctsk		
Dab2		
F8		
Fbn1		
Gsn		
Hmgcs2		
Lrp1		
Mmp14		
Mmp9		
Mst1		
Omp		
Pecam1		
Pros1		
Prss23os		
Serpine1		
Sparc		
Tmprss6		

IFN-α response

Symbol	C	H
Cnp		
Gbp3		
Gm36823		
Herc6		
Ifih1		
Il15		
Il7		
Lap3		
Ncoa7		
Olfr654		
Parp12		
Psmb9		
Psme2		
Rsad2		
Trafd1		
Trim14		
Txnip		
Uba7		

IFN-γ response

Symbol	C	H
Arid5b		
Bank1		
Btg1		
Casp3		
Ccl5		
Cdkn1a		
Gbp3		
Gch1		
Gm44104		
H2-Aa		
Herc6		
Il15		
Il7		
Irf4		
Lap3		
Nfkbia		
Nod1		
Parp12		
Psmb2		
Psmb9		
Ptgs2		
Ripk1		
Rsad2		
Sppl2a		
Stat3		
Tnfaip6		
Trafd1		
Trim14		
Txnip		

ROS pathway

Symbol	C	H
Ipcef1		
Gpx3		
Nqo1		
Prdx2		
Prdx6		

IL6/JAK/STAT3

Symbol	C	H
Il17ra		
Il1r1		
Jun		
Stat3		
Tnfrsf21		

KRAS signaling up

Symbol	C	H
Aldh1a2		
Apod		
AW551984		
BC051142		
Cidea		
Dcbld2		
Ets1		
Etv4		
Etv5		
F13a1		
Gadd45g		
Gpnmb		
Gprc5b		
Igf2		
Lcp1		
Lif		
Map3k1		
Mmp9		
Mpzl2		
Mycn		
Nr1h4		
Pecam1		
Peg3		
Plek2		
Ptgs2		
Satb1		
Scn1b		
Snap25		
Spp1		
Tor1aip2		
Tph1		
Traf1		
Trib1		
Wnt7a		
Zfp639		

Protein secretion

Symbol	C	H
Airn		
Ap1g1		
Atp6v1b1		
Bhlhe41		
Dst		
Golga4		
Mon2		
Pam		
Rps6ka3		
Scrn1		
Tmed2		

Allograft rejection

Symbol	C	H
Abi1		
Capg		
Cartpt		
Csk		
Ets1		
Gcnt1		
Ikbkb		
Il16		
Mmp9		
Npm1		
Prkcb		
Prkcg		
Tcp11l1		
Tgfb2		
Was		
Zap70		

KRAS signaling down

Symbol	C	H
4933423P22Rik		
Bard1		
Brdt		
Calca		
Ccna1		
Celsr2		
Cmbl		
Col2a1		
Cyp39a1		
Entpd7		
Gtf3c5		
Hsd11b2		
Htr1d		
Igfbp2		
Irs4		
Park2		
Pax3		
Rsad2		
Sgk1		
Sidt1		
Skil		
Slc16a7		
Slc30a3		
Slc38a3		
Tenm2		
Tgfb2		
Zbtb16		
Zfp112		

Inflammatory response

Symbol	C	H
Abi1		
Aplnr		
Best1		
Cdkn1a		
Fzd5		
Il1r1		
Irak2		
Ldlr		
Mmp14		
Nfkbia		
Prok2		
Raf1		
Scn1b		
Sema4d		
Slc31a1		
Slc31a2		
Slc7a2		
Tnfaip6		

Apical surface

Symbol	C	H
Afap1l2		
Cd160		
Rhcg		
Rtn4rl1		
Slc2a4		

Hedgehog signaling

Symbol	C	H
Adgrg1		
Gli1		
Hey2		
Nf1		
Scg2		

* Kirsten rat sarcoma viral oncogene homologue (KRAS); protoporphyrin IX iron complex (HEME) metabolism

Table C. Statistically significant enriched gene expression of Kyoto Encyclopedia-defined Genes and Genomes (KEGGs) involved in metabolic pathways modulated by 808 nm tPBMT for the cortex (C) and hippocampus (H).

Adipogenesis

Symbol	C	H
Acly		
Aldoa		
Araf		
Atl2		
Baz2a		
Bcl6		
Cidea		
Crat		
Dhcr7		
Enpp2		
Fzd4		
Gm44716		
Gpx3		
Idh1		
Idh3g		
Immt		
Itga7		
Lipe		
Lpcat3		
Lpl		
Mgll		
Nabp1		
Ndufb7		
Nmt1		
Pfkfb3		
Phldb1		
Qdpr		
Rab34		
Retsat		
Slc25a1		
Slc27a1		
Sqor		
Stom		
Ucp2		

Xenobiotic metabolism

Symbol	C	H
Aldh9a1		
Catsper3		
Cda		
Cyfip1		
Dhps		
Dhrs1		
Elovl5		
Entpd5		
Epha2		
Esr1		
Gad1		
Idh1		
Igfbp4		
Il1r1		
Nmt1		
Nqo1		
Pdk4		
Pgd		
Pros1		
Ptgds		
Pycr1		
Retsat		
Slc35d1		
Slc6a6		
Tdo2		
Tgfb2		
Vtn		

Myogenesis

Symbol	C	H
Acsl1		
Actn3		
Adcy9		
Aebp1		
Agl		
Aplnr		
Apod		
Bhlhe40		
Bhlhe41		
Camk2b		
Casq2		
Cdh13		
Cdkn1a		
Chn1os3		
Col1a1		
Col3a1		
Col6a2		
Col6a3		
Crat		
Dmpk		
Fabp3		
Fdps		
Fhl1		
Fkbp1b		
Gaa		
Gpx3		
Gsn		
Igfbp7		
Itga7		
Itgb1		
Itgb4		
Kifc3		
Large1		
Myl3		
Myoz1		
Nav2		
Nqo1		
Palb2		
Pick1		
Ppp1r3c		
Psen2		
Pvalb		
Scd1		
Schip1		
Sparc		
Tpm3		
Zfp862-ps		

NOTCH signaling

Symbol	C	H
Ccnd1		
Dcbld2		
Fzd5		
Heyl		
Jag1		
Maml2		
Prkca		
Psen2		

Apical junction

Symbol	C	H
AC161252.1		
Actn3		
Adam23		
Akt3		
Arhgef6		
Cadm3		
Calb2		
Cdh1		
Cdh4		
Cldn14		
Cldn15		
Cldn5		
Col16a1		
Crat		
Fbn1		
Fscn1		
Gm15577		
Ikbkg		
Insig1		
Itgb1		
Itgb4		
Layn		
Mmp9		
Mpzl2		
Msn		
Mvd		
Nectin2		
Nectin4		
Nf1		
Pcdh1		
Pecam1		
Traf1		
Vasp		
Vav2		
Wasl		
Wnk4		
Ywhah		

Androgen response

Symbol	C	H
Arid5b		
Ccnd1		
Elovl5		
Gpsm1		
Hmgcr		
Hmgcs1		
Hpgd		
Idi1		
Insig1		
Nkx3-1		
Rps6ka3		
Sat1		
Scd1		
Sgk1		
Ssr2		
Tsc22d1		

P53 pathway

Symbol	C	H
Abat		
Aen		
AW551984		
Btg1		
Cdh13		
Cdkn1a		
Cyfip1		
Dcxr		
Ddit4		
Dram1		
Epha2		
Fbxw7		
Fgf13		
Hist1h1c		
Irak1		
Itgb4		
Jun		
Kif13b		
Mapkapk3		
Mt2		
Nnat		
Osgin1		
Perp		
Pgm2l1		
Phlda3		
Plk3		
Ralgds		
Retsat		
Rrad		
S100a3		
Sat1		
Steap3		
Stom		
Tprkb		
Traf4		
Trafd1		
Tsc22d1		
Txnip		
Zbtb16		
Zfp36l1		
Zfp862-ps		

Bile acid metabolism

Symbol	C	H
Abca4		
Acsl1		
Aldh9a1		
Ar		
Atxn1		
Bmp6		
Cyp39a1		
Hsd17b4		
Idh1		
Idi1		
Lipe		
Nr3c2		
Paox		
Paqr8		
Pex11a		
Pex19		
Pipox		
Pxmp2		
Rbp1		
Retsat		
Slc27a2		
Ttr		

MTORC1 signaling

Symbol	C	H
Acly		
Aldoa		
Bhlhe40		
Bub1		
Calr		
Cdkn1a		
Coro1a		
Ddit4		
Dhcr7		
Elovl5		
Hmgcr		
Hmgcs1		
Hspa5		
Idh1		
Idi1		
Immt		
Insig1		
Ldlr		
mt-Rnr1		
Nfil3		
Nfkbib		
Nmt1		
Pik3r3		
Plod2		
Pno1		
Polr3g		
Ppia		
Psat1		
Qdpr		
Rrm2		
Scd1		
Sdf2l1		
Slc6a6		
Sqle		
Stard4		
Sytl2		
Tfrc		
Xbp1		

G2M checkpoint

Symbol	C	H
Arid4a		
Bard1		
Cbx1		
Ccnd1		
Cdk4		
Cenpe		
E2f4		
Fbxo5		
Hist2h2bb		
Hmgb3		
Hus1		
Mad2l1		
Mcm6		
Meis1		
Meis2		
Nsd2		
Nup98		
Smad3		
Sqle		
Srsf2		
Tmem129		
Ube2s		

Estrogen response early

Symbol	C	H
Abat		
Adcy1		
Adcy9		
Ar		
Bhlhe40		
Calb2		
Ccnd1		
Celsr2		
Chpt1		
Dhcr7		
Elovl5		
Esrp2		
Fasn		
Fhl2		
Gfra1		
Igf1r		
Igfbp4		
Inpp5f		
Kazn		
Lrig1		
Med13l		
Nav2		
Nbl1		
Npy1r		
Olfm1		
Pex11a		
Pgr		
Rapgefl1		
Ret		
Scnn1a		
Sh3bp5		
Slc16a1		
Slc22a21		
Slc27a2		
Slc7a2		
Stc2		
Tbc1d30		
Tfap2c		
Tgif2		
Tjp3		
Xbp1		
Zfp185		

TGF-β signaling

Symbol	C	H
Bmpr2		
Ctnnb1		
Id3		
Ski		
Skil		
Smad3		
Smurf1		

Unfolded protein response

Calr		
Ddit4		
Fam57a		
Hspa5		
Nabp1		
Psat1		
Tmem189		
Xbp1		

Estrogen response late

Symbol	C	H
Aldh3b3		
Batf		
Cav1		
Ccna1		
Ccnd1		
Cdh1		
Celsr2		
Chpt1		
Dhcr7		
Elovl5		
Emp2		
Hmgcs2		
Igfbp4		
Large1		
Llgl2		
Mest		
Nbl1		
Npy1r		
Olfm1		
Perp		
Pgr		
Plxnb1		
Ppif		
Prkar2b		
Prss23os		
Rabep1		
Rapgefl1		
Ret		
Scnn1a		
Sgk1		
Slc16a1		
Slc22a21		
Slc27a2		
Tmprss3		
Xbp1		
Xrcc3		

IL-2-STAT5 signaling

Symbol	C	H
Airn		
Batf		
Bhlhe40		
Bmpr2		
Capg		
Capn3		
Casp3		
Col6a1		
Ctbp2		
Cyfip1		
Etv4		
Fam126b		
Igf1r		
Lrig1		
Lrrc8c		
Nfil3		
Plec		
Rora		
Rragd		
Slc39a8		
Spp1		
Tnfrsf21		
Tnfrsf8		
Traf1		
Ttc39b		
Xbp1		

Mitotic spindle

Symbol	C	H
Abi1		
Akap13		
Anln		
Apc		
Arhgef3		
Cdc42ep4		
Cdk1		
Cenpe		
Cenpj		
Dock4		
Dst		
Dync1h1		
Dynll2		
Ect2		
Epb41		
Ezr		
Fam57a		
Fgd4		
Fscn1		
Gm31749		
Gsn		
Kntc1		
Llgl1		
Mapre1		
Marcks		
Mid1		
Mid1ip1		
Nck2		
Net1		
Nf1		
Prex1		
Pxn		
Rapgef5		
Rasa2		
Smc3		
Sorbs2		
Trdn		
Wasl		

Spermatogenesis

Symbol	C	H
Ccna1		
Chfr		
Cnih2		
Gad1		
Gsg1		
Hspa2		
Ncaph		
Strbp		
Tle4		

WNT/β-catenin signaling

Symbol	C	H
Adam17		
Ctnnb1		
Hey2		
Jag1		
Psen2		
Wnt1		
Wnt5b		
Wnt6		

Table D. Statistically significant enriched gene expression of Kyoto Encyclopedia-defined Genes and Genomes (KEGGs) involved in metabolic pathways modulated by 808 nm tPBMT for the cortex (C) and hippocampus (H).

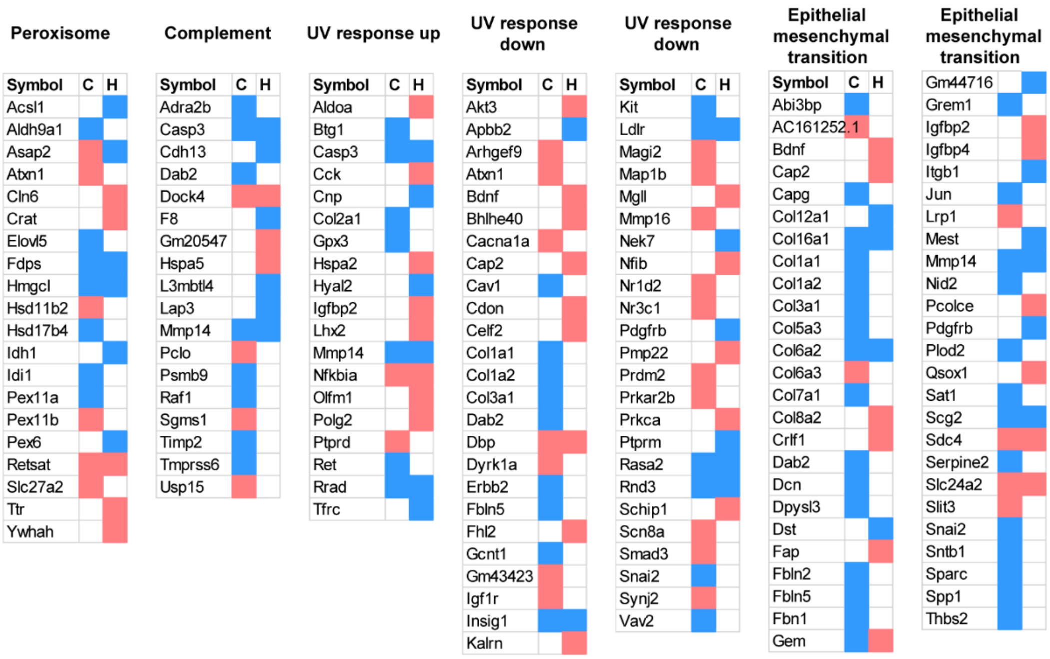

Analysed genes

Apoptosis pathway (107 genes)

AC122372.3, Add1, Airn, Anxa1, Atf3, Bax, Bcap31, Bcl2l1, Bcl2l11, Bgn, Bmf, Bmp2, Brca1, Btg2, Btg3, Casp2, Casp3, Casp6, Casp9, Cav1, Ccna1, Ccnd1, Ccnd2, Cd44, Cdc25b, Cdk2, Cdkn1a, Cdkn1b, Crebbp, CT030184.4, Ctnnb1, Cyld, Dap, Dap3, Dcn, Ddit3, Dffa, Diablo, Dnaja1, Dpyd, Egr3, Emp1, Eno2, Erbb2, Erbb3, Etf1, F2, F2r, Fdxr, Fez1, Gadd45a, Gch1, Gm43423, Gm8566, Gpx3, Gpx4, Gsn, Gsr, Gstm7, Gucy2e, Hgf, Hmgb2, Il1b, Irf1, Jun, Krt18, Lef1, Lgals3, Lmna, Madd, Mcl1, Mmp2, Nedd9, Nefh, Pak1, Pdgfrb, Pea15a, Plcb2, Plppr4, Ppp1r3e, Ppp2r5b, Ppp3r1, Ppt1, Psen1, Psen2, Rara, Retsat, Rhob, Rhot2, Rnasel, Sat1, Satb1, Slc20a1, Smad7, Sod2, Sptan1, Sqstm1, Tap1, Tgfb2, Timp1, Timp2, Timp3, Top2a, Txnip, Vdac2, Wee1, Zranb3.

Cholesterol homeostasis pathway (48 genes)

1700082M22Rik, Abca2, Acat2, Acss2, Actg1, Alcam, Aldoc, Antxr2, Anxa5, Atf3, Atf5, Atxn2, Cbs, Cct2, Cd9, Chka, Cpeb2, Ctnnb1, Dhcr7, Ech1, Errfi1, Fasn, Fdps, Gusb, Hmgcr, Hmgcs1, Idi1, Jag1, Ldlr, Lgals3, Lgmn, Lpl, Lss, Mal2, Mvd, Mvk, Nfil3, Pcyt2, Pnrc1, Pparg, S100a11, Scd1, Sema3b, Sqle, Stard4, Stx5a, Tm7sf2, Trp53inp1.

MYC targets V1 pathway (170 genes)

2310002F09Rik, 4933406I18Rik, Abce1, AC117232.1, AC162801.2, Acp1, Aimp2, Apex1, Aqp11, Arhgap25, Bub3, C1qbp, Cad, Canx, Cbx3, Ccna2, Cct2, Cct4, Cct5, Cct7, Cdc20b, Cdc45, Cdk2, Cdk4, Cfap46, Cnbp, Cops5, Cstf2, CT485612.11, Ctps, Cul1, Ddx21, Dek, Dhx15, Eef1b2, Eif1ax, Eif2s1, Eif2s2, Eif3b, Eif3d, Eif3j1, Eif4a1, Eif4e, Eif4g2, Eif4h, Eprs, Erh, Etf1, Exosc7, Fam120a, Fbl, G3bp1, Gm16546, Gm4950, Gm8173, Got2, Hdac2, Hddc2, Hdgf, Hnrnpa1, Hnrnpa2b1, Hnrnpd, Hnrnpr, Hnrnpu, Hprt, Hsp90ab1, Iars, Ifrd1, Ilf2, Impdh2-ps, Klhl22, Kpna2, Kpnb1, Ldha, Lsm2, Lsm7, Mad2l1, Mcm2, Mcm5, Mcm6, Mcph1, Mrpl23, Mrpl9, Mrps18b, Myc, Nap1l1, Ncbp2, Nkiras1, Nolc1, Nop16, Nop56, Npm1, Orc2, Pabpc4, Pcna, Pgk1, Phb, Phb2, Pold2, Ppia, Ppm1g, Prdx3, Prdx4, Prpf31, Prps2, Psma2, Psma4, Psma6, Psma7, Psmb2, Psmc4, Psmd1, Psmd14, Psmd3, Psmd7, Psmd8, Ptges3, Pwp1, Rack1, Rad23b, Ran, Ranbp1, Rfc4, Rnf207, Rpl18, Rpl34, Rps10, Rps3, Rps5, Rps6, Rrm1, Rrp9, Ruvbl2, Serbp1, Set, Sf3a1, Sf3b3, Slc25a3, Smarcc1, Snrpa, Snrpa1, Snrpb2, Snrpd2, Snrpd3, Srm, Srsf1, Srsf2, Srsf3, Srsf7, Ssb, Ssbp1, Stard7, Syncrip, Tardbp, Tfdp1, Tra2b, Trim28, Tufm, Txnl4a, Tyms, Uba2, Vbp1, Vdac1, Vdac3, Xpa, Xpo1, Xpot, Xrcc6, Ywhae, Ywhaq.

MYC targets V2 pathway (46 genes)

AC117232.1, AC162801.2, Aimp2, Bysl, Cbx3, Cdk4, Dctpp1, Dusp2, Exosc5, Farsa, Gm26316, Gm28306, Gm8173, Grwd1, Hk2, Ipo4, Las1l, Mcm5, Mphosph10, Mrto4, Mybbp1a, Myc, Noc4l, Nolc1, Nop16, Nop2, Nop56, Npm1, Pes1, Phb, Plk1, Plk4, Ppan, Pprc1, Prmt3, Pus1, Rcl1, Rrp9, Slc29a2, Srm, Supv3l1, Tbrg4, Tcof1, Tfb2m, Ung, Utp20.

DNA repair pathway (108 genes)

1700018L02Rik, 1700121C08Rik, AC122379.2, AC155713.1, Ada, Adrm1, Alyref, Aqp11, Arl6ip1, Bcam, Bcap31, Brf2, Cant1, Cda, Cetn2, Clp1, Cmpk2, Cox17, Cstf3, Dad1, Dctn4, Ddb2, Dgcr8, Dguok, Eif1b, Ercc1, Ercc2, Ercc4, Ercc8, Gm10143, Gm16253, Gm17954, Gm29331, Gm44898, Gm9172, Gpx4, Gtf2a2, Gtf2f1, Gtf2h1, Gtf2h3, Gtf3c5, Hcls1, Hprt, Impdh2-ps, Itpa-ps1, Lig1, Ncbp2, Nelfb, Nelfcd, Nelfe, Nfx1, Nme3, Npr2, Nt5c, Nt5c3, Nudt21, P3h2, Pcna, Pde4b, Pold1, Pold3, Poll, Polr1d, Polr2a, Polr2c, Polr2e, Polr2i, Polr2j, Polr2k-ps, Polr3c, Prim1, Rad51, Rad52, Rae1, Rev3l, Rfc2, Rfc3, Rfc4, Rfc5, Rnmt, Rpa2, Rpa3, Sac3d1, Sdcbp, Sec61a1, Sf3a3, Smad5, Snapc4, Srsf6, Ssrp1, Stx3, Supt4a, Supt5, Taf10, Taf12, Taf6, Tars2, Tk2, Tmed2, Trp53, Tsg101, Tyms, Umps, Upf3b, Usp11, Vps37b, Vps37d, Znrd1.

Notch signaling pathway (27 genes)

Arrb1, Ccnd1, Cul1, Dcbld2, Dll1, Dtx1, Dtx2, Dtx4, Fbxw11, Fzd1, Fzd5, Fzd7, Gm15429, Hes1, Heyl, Jag1, Kat2a, Lfng, Maml2, Notch2, Prkca, Psen2, Sap30, Skp1a, Tcf7l2, Wnt2, Wnt5a.

Hypoxia pathway (165 genes)

9530052E02Rik, AC091237.1, Ackr3, Adm, Adora2b, Ak4, Aldoa, Aldob, Aldoc, Ampd3, Angptl4, Ankzf1, Anxa2, Atf3, Atp7a, B3galt6, Bcan, Bcl2, Bgn, Bhlhe40, Brs3, Btg1, Car12, Casp6, Cav1, Cavin1, Ccng2, Cdkn1a, Cdkn1b, Cdkn1c, Chst2, Chst3, Cp, Crmp1, Csrp2, Cxcr4, Dcn, Ddit3, Ddit4, Dnajc13, Dtna, Dusp1, Edn2, Efna1, Efna3, Egfr, Eno2, Eno3, Errfi1, Ets1, Ext1, Fanca, Fbp1, Fos, Fosl2, Foxo3, Gaa, Gapdh, Gbe1, Gcnt2, Gfm1, Gm11534, Gm16379, Gm27401, Gm4832, Gm4907, Gm5855, Gpc1, Gpc4, Gpi1, Grhpr, Has1, Hdlbp, Hexa, Hk1, Hk2, Hs3st1, Hspa5, Ids, Igfbp1, Igfbp3, Ilvbl, Inha, Itgb4, Jun, Kdelr3, Kdm3a, Kif5a, Klf6, Klf7, Klhl24, Large1, Ldha, Lox, Map3k1, Mxi1, Myh9, Nagk, Ncan, Ndrg1, Ndst1, Ndst2, Nfil3, Noct, Nr3c1, P4ha1, Pam, Pck1, Pdk1, Pfkfb3, Pfkl, Pfkp, Pgam2, Pgf, Pgk1, Phkg1, Pklr, Pkp1, Pnrc1, Ppargc1a, Ppfia4, Ppp1r15a, Ppp1r3c, Prdx5, Prkca, Pygm, Rbpj, Rhobtb3, Rora, Rragd, Ruvbl2, S100a3, Sap30, Scarb1, Sdc2, Sdc3, Sdc4, Selenbp2, Serpine1, Siah2, Slc22a5, Slc25a1, Slc2a1, Slc2a3, Slc2a5, Slc37a4, Slc6a6, Srpx, Stc2, Tgfbi, Tiparp, Tmem45a, Tnfaip3, Tpbg, Tpd52, Tpi1, Ugp2, Vegfa, Vhl, Vldlr, Wsb1, Xpnpep1, Zfp292, Zfp36, Zranb3.

Interferon-α response pathway (58 genes)

Adar, Cd47, Cd74, Cmpk2, Cmtr1, Cnp, Csf1, Ddx60, Dhx58, Gbp2b, Gbp3, Gm11131, Gm11694, Gm20496, Gm36823, Gmpr, Helz2, Herc6, Ifi35, Ifih1, Ifit2, Il15, Il7, Irf1, Irf2, Irf7, Irf9, Lap3, Lgals3bp, Mov10, Mvb12a, Ncoa7, Nub1, Oas3, Oasl1, Ogfr, Olfr654, Parp12, Pnpt1, Psma3, Psmb9, Psme1, Psme2, Rb1, Rnf31, Rsad2, Samd9l, Sell, Slc25a28, Stat2, Tap1, Tdrd7, Trafd1, Trim14, Trim26, Txnip, Uba7, Wars.

Interferon-γ response pathway (126 genes)

Adar, Arid5b, Arl4a, Auts2, Bank1, Bhlhe41, Bpgm, Btg1, C1ra, Casp3, Ccl5, Cd74, Cdkn1a, Cfb, Ciita, Cmklr1, Cmpk2, Cmtr1, Csf2rb2, Ddx27, Ddx58, Ddx60, Dhx58, Fanca, Gbp3, Gbp6, Gch1, Gm11694, Gm20496, Gm44104, Gm6300, H2-Aa, H2-DMa, H2-M5, H2-Q10, Helz2, Herc6, Hif1a, Ifi35, Ifih1, Ifit2, Il15, Il18bp, Il2rb, Il7, Irf1, Irf2, Irf4, Irf5, Irf7, Irf8, Irf9, Itgb7, Jak2, Lap3, Lats2, Lcp2, Lgals3bp, Lysmd2, March1, Mettl7b, Mthfd2, Mvp, Mx1, Myd88, Nampt, Ncoa3, Nfkb1, Nfkbia, Nlrc5, Nod1, Nup93, Oas3, Oasl1, Ogfr, P2ry14, Parp12, Pde4b, Peli1, Pfkp, Pla2g4a, Pml, Pnpt1, Psma2, Psma3, Psmb10, Psmb2, Psmb9, Psme1, Psme2, Ptgs2, Ptpn1, Ptpn2, Ptpn6, Rapgef6, Rbck1, Ripk1, Rnf31, Rsad2, Samd9l, Selp, Serping1, Slc25a28, Socs3, Sod2, Sppl2a, Sri, St3gal5, St8sia4, Stat1, Stat2, Stat3, Tap1, Tdrd7, Tnfaip2, Tnfaip3, Tnfaip6, Tor1b, Trafd1, Trim14, Trim26, Txnip, Upp1, Vamp8, Vcam1, Wars.

Fatty acid metabolism pathway (121 genes)

Acaa1a, Acadm, Acads, Acadvl, Acat2, Aco2, Acot3, Acot8, Acsl1, Acsl4, Acsl5, Acsm3, Acss1, Adh7, Adipor2, Adsl, Alad, Aldh3a2, Aldh9a1, Aldoa, Aoc3, Apex1, Auh, Bcat2, Bckdhb, Blvra, Bmpr1b, Car2, Car4, Catsper3, Ccdc58, Cd36, Cel, Cfap126, Cidea, Cpt1a, Cpt2, Crat, Cyp1a1, Dld, Dlst, Ech1, Echs1, Ehhadh, Elovl5, Eno2, Eno3, Ephx1, Erp29, Etfdh, Fabp2, Fasn, Fh1,

Gabarapl1, Gad2, Glul, Gm16379, Gm3160, Gm43398, Gm7889, Gpd1, Gpd2, Grhpr, Gstz1, Hadh, Hadhb, Hao2, Hccs, Hmgcl, Hmgcs1, Hmgcs2, Hpgd, Hsd17b10, Hsd17b4, Hsdl2, Hsp90aa1, Hsph1, Idh1, Idh3b, Idh3g, Idi1, Il4i1, Kmt5a, Ldha, Mcee, Mdh1, Mdh2, Me1, Metap1, Mgll, Mlycd, Nbn, Ostc, Pcbd1, Pdha1, Pdhb, Ppara, Prdx6, Psme1, Ptprg, Rap1gds1, Rdh12, Rdh16, Reep6, Retsat, Rps6-ps1, S100a10, Sco2, Sdha, Serinc1, Slc22a21, Sms, Sucla2, Suclg1, Suclg2, Tdo2, Trp53inp2, Tsix, Ugdh, Urod, Ywhah.

KRAS signaling up pathway (146 genes)

4930519F16Rik, Abcb1b, AC122372.3, AC153845.3, Ace, Adam17, Adgra2, Adgrg3, Akt2, Aldh1a2, Aldh1a3, Angptl4, Ano1, Anxa10, Apod, Arg1, Avl9, AW551984, BC051142, Bmp2, Bpgm, Btc, C3ar1, Cab39l, Car2, Cbr4, Cbx8, Ccnd2, Ccser2, Cd37, Cdadc1, Cfb, Cidea, Cmklr1, Cpe, Crot, CT025616.1, Cxcr4, Dcbld2, Dnmbp, Dusp6, Emp1, Eng, Epb41l3, Ephb2, Ets1, Etv1, Etv4, Etv5, Evi5, F13a1, Fbxo4, Fcer1g, Fgf9, Flt4, Fuca1, Gabra3, Gadd45g, Gfpt2, Gm12079, Gm20496, Gm9200, Gng11, Gpnmb, Gprc5b, Gypc, Hdac9, Hkdc1, Hsd11b1, Igf2, Igfbp3, Ikzf1, Il1b, Il2rg, Inhba, Irf8, Itga2, Itgb2, Jup, Kcnn4, Kif5c, Laptm5, Lat2, Lcp1, Lif, Mafb, Map3k1, Map4k1, Mapk14, Mmd, Mmp11, Mmp9, Mpzl2, Mtmr10, Mycn, Nin, Nr1h4, Nrp1, Nxpe1-ps, Pcsk1n, Pecam1, Peg3, Pigr, Plek2, Ppp1r15a, Prdm1, Prelid3b, Prkg2, Prrx1, Ptbp2, Ptcd2, Ptgs2, Rabgap1l, Reln, Rhobtb3, Satb1, Scg3, Scg5, Scn1b, Sdccag8, Sema3b, Snap25, Snap91, Sox9, Sparcl1, Spon1, Spp1, Spry2, Tlr8, Tmem100, Tmem158, Tnfaip3, Tnfrsf1b, Tnnt2, Tor1aip2, Tph1, Traf1, Trib1, Trib2, Tspan13, Tspan7, Ush1c, Wnt7a, Yrdc, Zfp639, Zmat4.

KRAS signaling down pathway (135 genes)

4933423P22Rik, Abcg4, AC139382.1, AC154635.1, Adra2c, Ambn, Arhgdig, Arpp21, Asb7, Atp4a, Atp6v1b1, Bard1, Bmpr1b, Brdt, Btg2, Cacna1f, Calca, Capn9, Ccdc106, Ccna1, Cd207, Cdh16, Cdkal1, Celsr2, Chrng, Chst2, Ckm, Clstn3, Cmbl, Cntfr, Col2a1, Copz2, Coq8a, Cpa2, Cpeb3, Cyp39a1, Dlk2, Dtnb, Edar, Edn2, Efhd1, Entpd7, Epha5, Fgf16, Fgfr3, Fggy, Fshb, Gamt, Gdnf, Gm11940, Gm43618, Gp1ba, Gpr19, Gpr3, Gprc5c, Grid2, Gtf3c5, Hc, Hnf1aos1, Hsd11b2, Htr1b, Htr1d, Igfbp2, Irs4, Itgb1bp2, Kcnd1, Kcnn1, Kcnq2, Klhdc8a, Krt1, Krt15, Lfng, Mast3, Mfsd13b, Mfsd6, Msh5, Mthfr, Myh7, Myo15, Myot, Nos1, Npy4r, Nr4a2, Nr6a1, Nrip2, Ntf3, Nudt10, Oxt, P2ry4, Park2, Pax3, Pax4, Pde6b, Pdk2, Pkp1, Plag1, Pnmt, Rgs11, Rsad2, Ryr1, Selenop, Serpinb2, Sgk1, Shox2, Sidt1, Skil, Slc12a3, Slc16a7, Slc30a3, Slc38a3, Slc6a3, Smpx, Sncb, Sphk2, Stag3, Synpo, Tcf7l1, Tenm2, Tex15, Tfap2b, Tfcp2l1, Tg, Tgfb2, Thnsl2, Thrb, Tlx1, Tnni3, Tshb, Ugt2b34, Vps50, Wnt16, Ybx2, Zbtb16, Zc2hc1c, Zfp112.

Inflammatory response pathway (120 genes)

Abca1, Abi1, AC154635.1, Acvr1b, Acvr2a, Adm, Adora2b, Adrm1, Ahr, Aplnr, Atp2c1, Axl, Best1, Btg2, C3ar1, Calcrl, Ccl5, Ccr7, Cd82, Cdkn1a, Chst2, Clec5a, Cmklr1, Csf1, Csf3, Cx3cl1, Cybb, Dcbld2, Fanca, Ffar2, Fzd5, Gabbr1, Gch1, Gm11787, Gm12090, Gm27008, Gm29808, Gm42486, Gm4832, Gnat2, Gp1ba, Has2, Hif1a, Hpn, Hrh1, Il15, Il18r1, Il18rap, Il1b, Il1r1, Il2rb, Inhba, Irak2, Irf1, Irf7, Itgb8, Kcnj2, Kif1b, Klf6, Lck, Lcp2, Ldlr, Lif, Lpar1, Lta, Met, Mmp14, Msr1, Mxd1, Myc, Nampt, Nfkb1, Nfkbia, Nlrp3, Nod2, Oprk1, Osm, P2rx4, P2rx7, P2ry2, Pde4b, Pik3r5, Prok2, Psen1, Ptafr, Ptger4, Ptgir, Ptpre, Raf1, Rasgrp1, Rgs1, Rhog, Rnf144b, Scarf1, Scn1b, Selenos, Sell, Sema4d, Serpine1, Sgms2, Slamf1, Slc11a2, Slc1a2, Slc31a1, Slc31a2, Slc4a4, Slc7a1, Slc7a2, Sri, Stab1, Tacr1, Timp1, Tlr1, Tlr2, Tlr3, Tnfaip6, Tnfrsf1b, Tnfsf15, Tpbg, Vip.

E2F targets pathway (170 genes)

4930555F03Rik, AC117232.1, AC161609.1, AC173210.4, Ak2, Anp32e, Asf1a, Asf1b, Aurka, Bard1, Brca1, Brca2, Brms1l, Cbx5, Ccnb2-ps, Ccp110, Cdc20, Cdc25a, Cdc25b, Cdca3, Cdk1, Cdk4, Cdkn1a, Cdkn1b, Cdkn2c, Cenpe, Chek1, Chek2, Cit, Cks1b, Cnot9, Cse1l, CT485612.11, Ctcf, Ctps, Dck, Dclre1b, Dctpp1, Ddx39, Dek, Depdc1a, Dlgap5, Dnmt1, Dscc1, Eed, Eif2s1, Exosc8, Ezh2, Fcho2, Gins1, Gm13992, Gm14820, Gm2788, Gm31518, Gm31749, Gm6969, Hells, Hist1h2al, Hmgb2, Hmgb3, Hnrnpd, Hus1, Ilf3, Ing3, Ipo7, Jpt1, Kif18b, Kif2c, Kpna2, Lbr, Lig1, Lmnb1, Luc7l3, Lyar, Mad2l1, Mcm2, Mcm3, Mcm5, Mcm6, Melk, Mki67, Mms22l, Mre11a, Msh2, Mthfd2, Mybl2, Myc, Naa38, Nap1l1, Nasp, Nbn, Ncapd2, Nolc1, Nop56, Nudt21, Nup107, Nup153, Nup205, Orc2, Orc6, Pan2, Pcna, Pds5b, Plk1, Plk4, Pms2, Pnn, Pold1, Pold2, Pold3, Pole, Pop7, Ppm1d, Ppp1r8, Prdx4, Prps1, Psmc3ip, Pttg1, Rab24, Racgap1, Rad1, Rad50, Rad51c, Ran, Ranbp1, Rbbp7, Rfc1, Rfc2, Rfc3, Rnaseh2a, Rpa1, Rpa2, Rpa3, Rrm2, Shmt1, Slbp, Smc1a, Smc3, Smc4, Smc6, Snapc3, Snrpb, Spag5, Spc25, Srsf1, Srsf2, Ssrp1, Stag1, Syncrip, Tbrg4, Tcf19, Tfrc, Timeless, Tipin, Tk1, Tmem129, Tmpo, Top2a, Tra2b, Trip13, Trp53, Tubb5, Tubg1, Ube2s,

Ubr7, Ung, Wdr90, Wee1, Xpo1, Xrcc6.

Complement pathway (135 genes)

A930018P22Rik, AC130831.1, Adam9, Adra2b, Akap10, Anxa5, Atox1, C1ra, C3, Car2, Casp3, Casp9, Ccl5, Cd36, Cda, Cdh13, Cdk5r1, Cfb, Col4a2, Cp, Cpm, Cr2, Crry-ps, Csrp1, Ctsc, Ctsh, Ctso, Dab2, Dgkg, Dock10, Dock4, Dock9, Dpp4, Dusp6, Dyrk2, Ehd3, F2, F5, F7, F8, Fcer1g, Fn1, Gata3, Gca, Gm11762, Gm11787, Gm20481, Gm20547, Gm5881, Gnai2, Gnat2, Gnb2, Gnb4, Gngt2, Gp1ba, Gp9, Gpd2, Grb2, Gzmb, Hpcal4, Hspa5, Irf1, Irf2, Irf7, Itih1, Jak2, Kcnip3, Kif2a, Klkb1, Kynu, L3mbtl4, Lamp2, Lap3, Lck, Lcp2, Lgals3, Lgmn, Lrp1, Lta4h, Ltf, Me1, Mmp12, Mmp13, Mmp14, Mmp15, Msrb1, Mt3, Notch4, Pclo, Pdp1, Pfn1, Pik3ca, Pik3cg, Pik3r5, Pla2g4a, Pla2g7, Plek, Ppp4c, Prcp, Prep, Prkcd, Prss36, Psen1, Psmb9, Rabif, Raf1, Rasgrp1, Rbsn, Rce1, Rhog, Rnf4, Scg3, Serpinb2, Serpinc1, Serpine1, Serping1, Sgms1, Sh2b3, Spock2, Src, Stx4a, Timp1, Timp2, Tmem189, Tmprss6, Tnfaip3, Tyw1, Ubxn8, Usp14, Usp15, Usp16, Usp8, Was, Xpnpep1, Zeb1.

Unfolded protein response pathway (90 genes)

4933427G23Rik, Aldh18a1, Arfgap1, Asns, Atf3, Atf6, Atp6v0d1, Bag3, Calr, Cebpg, Chac1, Cks1b, Cnot4, Cnot6, Dcp1a, Dcp2, Dctn1, Ddit4, Dkc1, Dnaja4, Dnajb9, Edc4, Edem1, Eef2, Eif2ak3, Eif2s1, Eif4a1, Eif4a2, Eif4a3, Eif4e, Eif4g1, Ern1, Exoc2, Exosc5, Exosc9, Fam57a, Fkbp14, Fus, Gm14143, Gm44352, Gosr2, Herpud1, Hist1h2al, Hsp90b1, Hspa5, Iars, Igfbp1, Kdelr3, Khsrp, Kif5b, Lsm1, Lsm4, Mthfd2, Nabp1, Nfya, Nfyb, Nolc1, Nop14, Nop56, Npm1, Paip1, Pdia6, Pop4, Preb, Psat1, Rps14, Rrp9, Sdad1, Sec11a, Sec31a, Serp1, Shc1, Slc1a4, Slc7a5, Spcs3, Srpr, Ssr1, Stc2, Tatdn2, Tmem189, Tspyl2, Ttc37, Tubb2a, Vegfa, Wfs1, Xbp1, Xpot, Ywhaz, Zbtb17, Zfp738.

Glycolysis pathway (160 genes)

1700018L02Rik, 9530052E02Rik, Abcb6, AC091237.1, Adora2b, Agl, Agrn, Ak4, Akr1a1, Aldh7a1, Aldh9a1, Aldoa, Aldob, Alg1, Angptl4, Ankzf1, Arpp19, Artn, Aurka, B3galt6, B3gat1, B4galt1, B4galt2, B4galt7, Bpnt1, Cacna1h, Casp6, Cd44, Cdk1, Cfap126, Chpf, Chpf2, Chst1, Chst12, Chst2, Chst5, Cldn3, Cldn9, Cln6, Cog2, Copb2, Crmp1, Cxcr4, Dcn, Ddit4, Depdc1a, Dld, Efna3, Egfr, Elf3, Eno2, Ext1, Ext2, Fam149b, Fkbp4, Fut8, G6pdx, Gal3st1, Gale, Gclc, Gfpt1, Glce, Gm13992, Gm15243, Gm16379, Gm4832, Gm5855, Gm6741, Gm8566, Gmppa, Gmppb, Gne, Got1, Got2, Gpc1, Gpc4, Gpr87, Gusb, Hax1, Hdlbp, Hk2, Homer1, Hs2st1, Hs6st2, Hspa5, Idh1, Igfbp3, Itgb4, Kdelr3, Kif20a, Kif2a, Lct, Ldha, Lhpp, Lhx9, Lrfn4, Mdh1, Mdh2, Me1, Me2, Med24, Met, Miox, Mpi, Mxi1, Nasp, Ndst3, Nt5e, Omp, P4ha1, Pam, Paxip1, Pfkfb1, Pfkp, Pgam2, Pgk1, Pkm, Pkp2, Plod1, Plod2, Ppfia4, Ppia, Prps1, Psmc4, Pygb, Pygl, Qsox1, Rars, Rbck1, Rhobtb3, Rpe, Rragd, Ruvbl2, Sap30, Sdc2, Sdc3, Slc16a3, Slc22a5, Slc25a10, Slc25a13, Slc35a3, Slc35f6, Slc37a4, Sox9, Spag4, Ssr2, Stc2, Taldo1, Tgfa, Tgfbi, Tpbg, Tpi1, Tpst1, Ubxn8, Ugp2, Vcan, Vegfa, Vldlr, Xylt2, Zfp292.

TNFα signaling via NF-kB pathway (141 genes)

9530052E02Rik, Abca1, AC154635.1, Ackr3, Amz2, Atf3, B4galt1, B4galt5, Bcl2a1d, Bcl3, Bcl6, Bhlhe40, Bmp2, Btg1, Btg2, Btg3, Ccl5, Ccnd1, Ccnl1, Cd44, Cd83, Cdkn1a, Cebpd, Clcf1, Csf1, Cxcl1, Ddx58, Dennd5a, Dnajb4, Dram1, Dusp1, Dusp2, Dusp4, Efna1, Egr1, Egr2, Egr3, Ehd3, Eif1, Fjx1, Fos, Fosb, Fosl1, Fosl2, Gadd45a, Gch1, Gem, Gfpt2, Gm28592, Gm29808, Gm9947, Gpsm1, Hes1, Ier2, Ier5, Ifih1, Ifit2, Il1b, Il6st, Inhba, Irf1, Jag1, Jun, Kdm6b, Klf2, Klf6, Kynu, Lamb3, Ldlr, Lif, Litaf, Map2k3, Map3k8, Marcks, Mcl1, Mt2, Mxd1, Myc, Nampt, Nfat5, Nfe2l2, Nfil3, Nfkb1, Nfkbia, Nfkbie, Ninj1, Nr4a2, Nr4a3, Panx1, Pde4b, Pdlim5, Pfkfb3, Phlda2, Plek, Plk2, Pmepa1, Pnrc1, Ppp1r15a, Ptger4, Ptgs2, Ptpre, Ptx3, Rel, Relb, Rhob, Sat1, Sdc4, Serpinb2, Serpinb6b, Serpine1, Sgk1, Slc2a3, Slc2a6, Smad3, Socs3, Sod2, Spsb1, Sqstm1, Stat5a, Tank, Tap1, Tgif1, Tiparp, Tlr2, Tmem189, Tnc, Tnfaip2, Tnfaip3, Tnfaip6, Tnfaip8, Tnip1, Tnip2, Traf1, Trib1, Trip10, Tsc22d1, Tubb2a, Vegfa, Yrdc, Zc3h12a, Zfp36.

Reactive oxygen species pathway (41 genes)

Abcc1, Atox1, Cat, Cdkn2d, Egln2, Ercc2, Fes, Ftl1, G6pdx, Gclc, Gclm, Gm11505, Gm8566, Gpx3, Gpx4, Gsr, Hmox2, Ipcef1, Lamtor5, Lsp1, Mbp, Ndufs2, Nqo1, Oxsr1, Pdlim1, Pfkp, Ppm1n, Prdx1, Prdx2, Prdx4, Prdx6, Ptpa, Rhobtb3, Sbno2, Scaf4, Selenos, Sod2, Srxn1, Stk25, Txnrd1, Txnrd2.

Androgen response pathway (73 genes)

Abhd2, AC117232.1, AC122372.3, AC153845.3, Acsl3, Adamts1, Adrm1, Akt1, Aldh1a3, Arid5b, B4galt1, Bmpr1b,

Camkk2, Ccnd1, Ccnd3, Cdc14b, CT030161.3, Dnajb9, Dopey1, E030030I06Rik, Elk4, Ell2, Elovl5, Gm23623, Gnat2, Gpsm1, Gsr, Hmgcr, Hmgcs1, Homer2, Hpgd, Idi1, Inpp4b, Insig1, Itgav, Jak1, Krt19, Lman1, Ndrg1, Ngly1, Nkx3-1, Pdlim5, Pias1, Pmepa1, Ptk2b, Rab4a, Rps6ka3, Sat1, Scd1, Sec24d, Selenop, Sgk1, Slc26a2, Sms, Spcs3, Spdef, Srf, Srp19, Ssr2, Steap4, Stk39, Tefm, Tmem50a, Tmprss2, Tnfaip8, Tpd52, Tsc22d1, Uap1, Ube2i, Ube2j1, Xrcc5, Xrcc6, Zmiz1.

Heme metabolism pathway (152 genes)

1700034H15Rik, 2810407A14Rik, Abcb6, Abcg2, AC156802.2, Acp5, Acsl6, Add1, Add2, Adipor1, Agpat4, Aimp2, Alad, Alas2, Aldh6a1, Ank1, Aqp3, Arhgef12, Arl2bp, Asns, Atg4a, Atp6v0a1, B4galt3, Bcam, Blvra, Blvrb, Bpgm, Btg2, Btrc, C3, Car1, Car2, Cast, Cat, Ccdc28a, Ccnd3, Cdc27, Cir1, Clcn3, Ctns, Ctse, Dcaf10, Dcaf11, Dcun1d1, Dmtn, E2f2, Ell2, Epb41, Epb42, Epor, Ezh1, Fbxo7, Fbxo9, Fn3k, Foxj2, Foxo3, Gapvd1, Gata1, Gclc, Gclm, Gde1, Glrx5, Gm10244, Gm15756, Gmps, Gypc, Hdgf, Hmbs, Htatip2, Htra2, Igsf3, Isca1, Kat2b-ps, Kdm7a, Kel, Klf3, Lamp2, Lmo2, Lpin2, Lrfn4, Map2k3, March2, March8, Mark3, Mboat2, Mfhas1, Minpp1, Mocos, Mospd1, Mpp1, Mxi1, Myl4, Narf, Nek7, Nfe2, Nfe2l1, Nr3c1, Nudt4, Optn, Osbp2, Pdzk1ip1, Picalm, Pigq, Ppp2r5b, Pqlc1, Prdx2, Psmd9, Rad23a, Rap1gap, Rbm38, Rbm5, Rcl1, Rhd, Rnf123, Rnf19a, Sdcbp, Sec14l1, Selenbp2, Sidt2, Slc11a2, Slc22a4, Slc22a5, Slc25a37, Slc2a1, Slc30a10, Slc4a1, Slc6a8, Slc6a9, Slc7a11, Smox, Snca, Sptb, Synj1, Tal1, Tfdp2, Tfrc, Tmcc2, Tmem9b, Tns1, Top1, Trim10, Tspan5, Tyr, Ubac1, Ubl4a, Ucp2, Urod, Usp15, Vezf1, Ypel5, Zfp935, Zranb3.

Bile acid metabolism pathway (82 genes)

4930544I03Rik, Abca1, Abca2, Abca3, Abca4, Abca5, Abca8a, Abca8b, Abca9, Abcd1, Abcd2, Abcd3, Abcg4, Abcg8, Acsl1, Acsl5, Akr1d1, Aldh8a1, Aldh9a1, Apoa1, Ar, Atxn1, Bcar3, Bmp6, Cat, Ch25h, Crot, Cyp27a1, Cyp39a1, Cyp46a1, Cyp7a1, Cyp7b1, Dio1, Ephx2, Fdxr, Gclm, Gm8566, Gnmt, Gnpat, Hacl1, Hao1, Hsd17b4, Idh1, Idh2, Idi1, Lck, Lipe, Lonp2, Nedd4, Npc1, Nr1h4, Nr1i2, Optn, Paox, Paqr8, Pex1, Pex11a, Pex11g, Pex12, Pex13, Pex16, Pex19, Pex6, Pex7, Phyh, Pipox, Pnpla8, Prdx5, Pxmp2, Rbp1, Retsat, Rxrg, Scp2-ps2, Slc23a2, Slc27a2, Slc29a1, Slco1a1, Tfcp2l1, Ttr.

Xenobiotic metabolism pathway (136 genes)

Abcc2, Abcc3, Abcd2, Aco2, Acp1, Acp2, Adh5, Adh7, Ahcy, Alas1, Aldh2, Aldh9a1, Aox1, Ap4b1, Arg1, Arpp19, Asl, Atoh8, Bcar1, Blvrb, Car2, Casp6, Cat, Catsper3, Cd36, Cda, Cfb, Crot, Cyfip1, Cyp1a1, Cyp1a2, Cyp27a1, Cyp2e1, Cyp2j9, Cyp2s1, Cyp4f13, Dcxr, Ddah2, Ddc, Dhps, Dhrs1, Ech1, Elovl5, Enpep, Entpd5, Epha2, Ephx1, Esr1, Etfdh, F11, Fbp1, G6pc, Gabarapl1, Gad1, Gch1, Gckr, Gclc, Gcnt2, Gm26666, Gm3160, Gm4756, Gnmt, Gsr, Gss, Gstt2, Hacl1, Hes6, Hgfac, Hprt, Hsd11b1, Idh1, Igf1, Igfbp1, Igfbp4, Il1r1, Irf8, Itih1, Itih4, Jup, Kynu, Lcat, Leap2, Lonp1, Lpin2, Lrfn4, Man1a, Mccc2, Mpp2, Mthfd1, Ndrg2, Nfs1, Ninj1, Nmt1, Npc1, Nqo1, Pdk4, Pdlim5, Pgd, Pgrmc1, Pink1, Pmm1, Por, Ppp1r14a, Pros1, Psmb10, Ptgds, Ptges3, Pycr1, Rap1gap, Retsat, Sar1b, Serpine1, Sertad1, Shmt2, Slc12a4, Slc1a5, Slc22a1, Slc35b1, Slc35d1, Slc6a12, Slc6a6, Smox, Ssr3, Tat, Tdo2, Tgfb2, Tmbim6, Tnfrsf1a, Tpst1, Ttpa, Tyr, Ugdh, Upb1, Upp1, Vtn, Zfp677.

Estrogen response early pathway (158 genes)

1700108N11Rik, 4930556N13Rik, A930001C03Rik, Abat, Abca3, Abhd2, Ablim1, AC151530.1, AC155241.2, Adcy1, Adcy9, Aff1, Aldh3b3, Amfr, Aqp3, Ar, Arl3, B4galt1, Bag1, Bcl11b, Bcl2, Bhlhe40, Blvrb, C430049E01Rik, Calb2, Calcr, Cant1, Car12, Cbfa2t3, Ccnd1, Cd44, Celsr2, Chml, Chpt1, Cish, Cldn7, Cxcl12, Cyp26b1, Dhcr7, Dhrs2, Dhrs3, Dlc1, Dynlt3, Egr3, Elf3, Elovl2, Elovl5, Esrp2, Fam102a, Farp1, Fasn, Fhl2, Fkbp4, Flnb, Fos, Gfra1, Gja1, Gm11940, Gm13170, Gm36823, Gm37103, Hes1, Hr, Igf1r, Igfbp4, Il17rb, Il6st, Inhbb, Inpp5f, Itpk1, Jak2, Kazn, Kcnk15, Kcnk5, Kdm4b, Krt15, Krt18, Krt19, Lad1, Lrig1, Mast4, Med13l, Med24, Mindy1, Mlph, Mpped2, Mrpl4, Myb, Mybbp1a, Mybl1, Myc, Myof, Nadsyn1, Nav2, Nbl1, Ncor2, Npy1r, Nrip1, Nxt1, Olfm1, Olfml3, P2ry2, Palb2, Pdlim3, Pdzk1, Pex11a, Pgr, Ppif, Prss23os, Rab17, Rapgefl1, Rara, Rasgrp1, Reep1, Ret, Retreg1, Rhobtb3, Scarb1, Scnn1a, Sec14l2, Sema3b, Sh3bp5, Siah2, Slc16a1, Slc19a2, Slc1a1, Slc1a4, Slc22a21, Slc24a3, Slc26a2, Slc27a2, Slc2a1, Slc39a6, Slc7a2, Slc7a5, Smok2b, Sox3, Stc2, Svil, Syngr1, Tbc1d30, Tfap2c, Tgif2, Tiam1, Tiparp, Tjp3, Tmem164, Tmprss3, Tob1, Tpbg, Tsku, Ttc39a, Ugcg, Unc119, Wfs1, Wwc1, Xbp1, Zfp185.

Estrogen response late pathway (151 genes)

1700108N11Rik, 3110082I17Rik, 4930432L08Rik, Abca3, Abhd2, AC151530.1, Aff1, Agr2, Aldh3a2, Aldh3b3, Amfr, Arl3, Ascl1, Ass1, Atp2b4, Bag1, Batf, Bcl2, Blvrb, Btg3, C430049E01Rik, Calcr, Car12, Car2, Cav1, Ccna1, Ccnd1, Cd44, Cd9,

Cdc20, Cdc6, Cdh1, Celsr2, Chml, Chpt1, Cish, Ckb, Cpe, Cxcl12, Cxcl14, Cyp26b1, Dcxr, Dhcr7, Dhrs2, Dlg5, Dnajc1, Dnajc12, Dusp2, Dynlt3, Egr3, Elovl5, Emp2, Fam102a, Farp1, Fgfr3, Fkbp4, Flnb, Fos, Gale, Gm11940, Gm17118, Gm37103, Gm45233, Hmgcs2, Homer2, Hprt, Hr, Hspa4l, Idh2, Igfbp4, Il17rb, Il6st, Itpk1, Jak2, Kcnk5, Kif20a, Klk11, Krt19, Lamc2, Large1, Llgl2, Lsr, Ltf, Mapk13, Mdk, Mest, Mettl3, Mocs2, Myb, Myof, Nab2, Nbl1, Ncor2, Npy1r, Nrip1, Nxt1, Olfm1, Palb2, Pdlim3, Pdzk1, Perp, Pgr, Pkp3, Plk4, Plxnb1, Ppif, Prkar2b, Prlr, Prss23os, Ptger3, Ptpn6, Rabep1, Rapgefl1, Ret, Rnaseh2a, Scarb1, Scnn1a, Scube2, Sema3b, Sgk1, Siah2, Slc16a1, Slc1a4, Slc22a21, Slc24a3, Slc26a2, Slc27a2, Slc29a1, Slc2a8, Slc7a5, Smok2b, Snx10, Sox3, St14, Stil, Tfap2c, Tiam1, Tjp3, Tmprss3, Tnnc1, Tob1, Top2a, Tpbg, Trim29, Tspan13, Ugdh, Unc13b, Wfs1, Xbp1, Xrcc3, Zfp36.

Pancreas β-cells pathway (33 genes)

2010107G23Rik, Abcc8, Akt3, Chga, Dcx, Dpp4, Elp4, Fanca, Foxa2, Foxo1, Gcg, Gm44352, Hnf1aos1, Ins2, Insm1, Isl1, Lmo2, Mafb, Nkx2-2, Nkx6-1, Pak3, Pax4, Pax6, Pcsk1, Pklr, Scgn, Sec11a, Slc2a2, Srp9, Sst, Stxbp1, Syt13, Vdr.

UV response up pathway (125 genes)

9130227L01Rik, Abcb1b, Acaa1a, Alas1, Aldoa, Amd1, Ap2s1, Apom, Aqp3, Arrb2, Asns, Atf3, Atp6v1f, Bak1, Bcl2l11, Bmp2, Btg1, Btg2, Btg3, Car2, Casp3, Cck, Ccnd3, Cdc34, Cdc5l, Cdk2, Cdkn1c, Cdkn2a, Cebpg, Chka, Chrna5, Clcn2, Cltb, Cnp, Col2a1, Cxcl1, Cyb5b, Cyb5r1, Cyp1a1, Ddx21, Dgat1, Dlg4, Dnaja1, Dnajb1, Eif2s3x, Eif5, Eno2, Ephx1, Fgf18, Fkbp4, Fos, Fosb, Furin, Gch1, Ggh, Gls, Gm10143, Gm11787, Gm15589, Gm16546, Gm3160, Gpx3, Grina, H2-T3, Hist1h2al, Hnrnpu, Hspa13, Hspa2, Htr7, Hyal2, Igfbp2, Il6st, Irf1, Lhx2, Mapk8ip2, Mark2, Mgat1, Mmp14, Mrpl23, Msx1, Nfkbia, Nptx2, Nptxr, Nupl1, Nxf1, Olfm1, Onecut1, Pdap1, Pdlim3, Polg2, Ppat, Ppif, Ppp1r2, Ppt1, Prkaca, Prkcd, Prpf3, Psmc3, Ptprd, Rab27a, Rasgrp1, Ret, Rfc4, Rhob, Rpn1, Rrad, Rxrb, Shox2, Sigmar1, Slc25a4, Slc6a12, Slc6a8, Sod2, Sqstm1, Stard3, Stip1, Stk25, Tap1, Tfrc, Tgfbrap1, Tmbim6, Tuba4a, Tyro3, Urod, Ykt6.

UV response down pathway (123 genes)

Abcc1, Acvr2a, Adora2b, Akt3, Amph, Anxa2, Anxa4, Apbb2, Arhgef9, Atp2b4, Atp2c1, Atrn, Atrx, Atxn1, Bckdhb, Bdnf, Bhlhe40, Bmpr1a, Cacna1a, Cap2, Cav1, Cdc42bpa, Cdkn1b, Cdon, Celf2, Col11a1, Col1a1, Col1a2, Col3a1, Dab2, Dbp, Ddah1, Dlc1, Dlg1, Dusp1, Dyrk1a, Efemp1, Erbb2, Fbln5, Fhl2, Fzd2, Gcnt1, Gja1, Gm29808, Gm38211, Gm43423, Gm43618, Gm5149, Gm9077, Has2, Ica1, Id1, Igf1r, Igfbp5, Inpp4b, Insig1, Irs1, Kalrn, Kcnma1, Kit, Lamc1, Ldlr, Lpar1, Magi2, Map1b, Map2k5, Mapk14, Met, Mgll, Mios, Mmp16, Mrps31, Msh3, Mta1, Myc, Nek7, Nfib, Nfkb1, Nipbl, Notch2, Nr1d2, Nr3c1, Nrp1, Pccb, Pcyox1, Pdgfrb, Pdlim5, Pex14, Phf3, Pias3, Pik3cd, Pik3r3, Plcb4, Pmp22, Pparg, Prdm2, Prkar2b, Prkca, Pten, Ptprm, Rasa2, Rbpms, Rgs4, Rnd3, Schip1, Scn8a, Sdc2, Serpine1, Sipa1l1, Slc7a1, Smad3, Smad7, Snai2, Sntg1, Sri, Syne1, Synj2, Tgfbr2, Togaram1, Vav2, Vldlr, Wdr37, Zmiz1.

Angiogenesis pathway (28 genes)

AC091237.1, Apoh, Ccnd2, Col3a1, Fgfr1, Fstl1, Gm38211, Itgav, Jag1, Jag2, Lpl, Lrpap1, Msx1, Nrp1, Pdgfa, Pf4, Postn, Prg2, S100a3, Slco2a1, Spp1, Thbd, Timp1, Tnfrsf21, Vav2, Vcan, Vegfa, Vtn.

Apical junction pathway (163 genes)

1700010K23Rik, 2600006K01Rik, 6820431F20Rik, AC161252.1, AC162938.3, Acta1, Actb, Actg1, Actg2, Actn3, Actn4, Adam15, Adam23, Adam9, Akt2, Akt3, Alox8, Amh, Amigo1, Arhgef6, Atp1a3, B4galt1, Baiap2, Cadm3, Calb2, Cap1, Cd276, Cdh1, Cdh15, Cdh3, Cdh4, Cdh6, Cdk8, Cldn14, Cldn15, Cldn4, Cldn5, Cldn6, Cldn7, Cldn9, Cnn2, Col16a1, Col17a1, Col9a1, Crat, CT030161.3, Ctnnd1, Cx3cl1, Dhx16, Dlg1, Dsc1, Egfr, Epb41l2, Evl, Exoc4, Fbn1, Flnc, Fscn1, Gamt, Gm14550, Gm15577, Gm15589, Gm16897, Gm21986, Gm28100, Gm37988, Gm42957, Gnai2, Grb7, Gtf2f1, Hadh, Hras, Icam5, Ikbkg, Inppl1, Insig1, Irs1, Itga2, Itga3, Itga9, Itgb1, Itgb4, Jam3, Jup, Krt33b, Lamb3, Lamc2, Layn, Ldlrap1, Lima1, Map3k20, Map4k2, Mapk11, Mapk13, Mapk14, Mdk, Mmp2, Mmp9, Mpzl1, Mpzl2, Msh3, Msn, Mvd, Myh10, Myh9, Nectin2, Nectin3, Nectin4, Nexn, Nf1, Nf2, Nfasc, Nlgn2, Nrap, Nrtn, Nrxn2, Parva, Pbx2, Pcdh1, Pdzd3, Pecam1, Pfn1, Pik3cb, Pik3r3, Plcg1, Ppp2r2c, Pten, Ptprc, Rasa1, Rhof, Rras, Rsu1, Sdc3, Shc1, Sirpa, Slc16a5, Slc30a3, Slit2, Speg, Src, Stx4a, Syk, Sympk, Taok2, Tgfbi, Thbs3, Tial1, Tmem8b, Tnfrsf11b, Traf1, Tro, Tspan4, Tubg1, Vasp, Vav2, Vcam1, Vcan, Vcl, Vwf, Wasl, Wnk4, Ywhah, Zyx.

Apical surface pathway (34 genes)

Adam10, Adipor2, Afap1l2, Atp6v0a4, B4galt1, Brca1, Cd160, Crocc, Cx3cl1, Dcbld2, Efna5, Ephb4, Flot2, Gas1, Gata3, Gm11787, Gpr160, Gstm5, Il2rb, Il2rg, Mal, Mdga1, Nars2, Ncoa6, Pkhd1, Rhcg, Rtn4ip1, Rtn4rl1, Scube1, Slc2a4, Slc34a3, Srpx, Sulf2, Tmem8b.

Hedgehog signaling pathway (32 genes)

Ache, Adgrg1, Amot, Cdk5r1, Cntfr, Crmp1, Dpysl2, E030030I06Rik, Gli1, Hey1, Hey2, L1cam, Ldb1, Myh9, Nf1, Nkx6-1, Nrcam, Nrp1, Nrp2, Pml, Ptch1, Rasa1, Rtn1, Scg2, Shh, Slit1, Tle1, Tle3, Trdn, Unc5c, Vegfa, Vldlr.

mTORC1 signaling pathway (166 genes)

AC091237.1, 1700082M22Rik, AC162801.2, Acaca, Acly, Acsl3, Actr2, Actr3, Adipor2, Ak4, Aldoa, Arpc5l, Asns, Aurka, Bhlhe40, Btg2, Bub1, Cacybp, Calr, Canx, Ccng1, Cct6a, Cd9, Cdc25a, Cdkn1a, Cops5, Coq7, Coro1a, Ctsc, Cxcr4, Cyb5b, Dapp1, Dbt, Ddit3, Ddit4, Ddx39, Dhcr7, Edem1, Eef1e1, Eif2s2, Elovl5, Eprs, Etf1, Fdxr, G6pdx, Gapdh, Gbe1, Gclc, Gga2, Gm26666, Gm35986, Gm37103, Gm37988, Gm4907, Gm5855, Gmps, Got1, Gpi1, Gsk3b, Gsr, Gtf2h1, Hk2, Hmbs, Hmgcr, Hmgcs1, Hprt, Hsp90b1, Hspa4, Hspa5, Idh1, Idi1, Ifrd1, Igfbp5, Immt, Insig1, Itgb2, Ldha, Ldlr, Lgmn, Lta4h, M6pr, Map2k3, Mcm2, Me1, Mllt11, Mpp5, Mthfd2, Mthfd2l, mt-Rnr1, Nampt, Nfil3, Nfkbib, Nfyc, Nmt1, Nufip1, Nup205, P4ha1, Pdap1, Pdk1, Pfkl, Pgk1, Pik3r3, Pitpnb, Plk1, Plod2, Pno1, Polr3g, Ppa1, Ppia, Ppp1r15a, Prdx1, Psat1, Psma3, Psma4, Psmb5, Psmc4, Psmd12, Psmd13, Psmd14, Psme3, Qdpr, Rab1a, Rdh12, Rhobtb3, Rit1, Rpa1, Rpn1, Rrm2, Rrp9, Scd1, Sdf2l1, Sec11a, Serp1, Serpinh1, Shmt2, Sla, Slc1a4, Slc1a5, Slc2a1, Slc2a3, Slc37a4, Slc6a6, Slc7a11, Slc7a5, Sqle, Sqstm1, Ssr1, Stard4, Stip1, Sytl2, Tbk1, Tfrc, Tm7sf2, Tomm40, Tpi1, Tuba4a, Tubg1, Txnrd1, Uchl5, Ufm1, Ung, Uso1, Vldlr, Wars, Xbp1, Ykt6.

Mitotic spindle pathway (176 genes)

0610038B21Rik, 2700099C18Rik, 6430550D23Rik, Abi1, Abl1, Abr, AC173210.4, Actn4, Akap13, Als2, Anln, Apc, Arf6, Arfgef1, Arfip2, Arhgap27, Arhgap29, Arhgdia, Arhgef12, Arhgef2, Arhgef3, Arhgef7, Arl8a, Atg4b, Aurka, Avpr2, Bcar1, Bcl2l11, Bcr, Bin1, Brca2, Bub1, Capzb, Ccnb2-ps, Cdc27, Cdc42bpa, Cdc42ep1, Cdc42ep2, Cdc42ep4, Cdk1, Cdk5rap2, Cenpe, Cenpf, Cenpj, Cep131, Cep192, Cep57, Cep72, Ckap5, Clasp1, Clip1, Clip2, Cntrl, Cntrob, Coq5, Csnk1d, CT030184.4, Cttn, Cyth2, Dlg1, Dlgap5, Dock4, Dst, Dync1h1, Dynll2, Ect2, Epb41, Epb41l2, Ezr, Fam57a, Farp1, Fbxo5, Fgd4, Fgd6, Flna, Flnb, Fscn1, Gm26740, Gm31749, Gm6204, Gm8242, Gm826, Gsn, Hdac6, Hook3, Itsn1, Katna1, Katnb1, Kif15, Kif1b, Kif20b, Kif2c, Kif3b, Kif3c, Kif5b, Kifap3, Klc1, Kntc1, Kptn, Lats1, Llgl1, Lmnb1, Lrpprc, Mapre1, Marcks, Mark4, Mid1, Mid1ip1, Myh10, Myh9, Myo1e, Myo9b, Nck2, Nedd9, Net1, Nf1, Nin, Notch2, Numa1, Nxpe1-ps, Pafah1b1, Palld, Pcm1, Pcnt, Pdlim5, Pif1, Pkd2, Plekhg2, Plk1, Ppp4r2, Prc1, Prex1, Pxn, Rab3gap1, Racgap1, Ralbp1, Ranbp9, Rapgef5, Rapgef6, Rasa1, Rasa2, Rfc1, Rhof, Rhot2, Sac3d1, Sass6, Slco6d1, Smc1a, Smc3, Smc4, Sorbs2, Sptan1, Sptbn1, Ssh2, Stau1, Synpo, Taok2, Tbcd, Tiam1, Tlk1, Top2a, Tpx2, Trdn, Ttk, Tuba4a, Tubd1, Tubgcp2, Tubgcp3, Tubgcp5, Tubgcp6, Vcl, Wasf1, Wasf2, Wasl, Ywhae.

Oxidative phosphorylation pathway (133 genes)

4930412C18Rik, 9630013K17Rik, Acaa1a, Acadm, Acadsb, Acadvl, Aco2, Afg3l2, Aifm1, Alas1, Aldh6a1, Atp1b1, Atp6ap1, Atp6v0b, Atp6v0c, Atp6v1e1, Atp6v1f, Atp6v1h, Bax, Bcat2, Bckdha, Bdh2, Cfap126, Cfap46, Cox15, Cox17, Cox6b1, Cox7a2, Cox7c, Cpt1a, Crx, Cs, Cyb5r3, Cycs, Dld, Dlst, Ech1, Echs1, Efcab5, Emc8, Etfa, Etfdh, Fh1, Glud1, Gm11585, Gm45233, Gm7180, Got2, Gpi1, Gpx4, Hadha, Hadhb, Hccs, Hsd17b10, Htra2, Idh1, Idh2, Idh3a, Idh3b, Idh3g, Immt, Isca1, Iscu, Ldha, Lrpprc, Maob, Mdh1, Mdh2, Mfn2, Mpc1, Mpp5, Mrpl15, Mrpl35, Mrps11, Mrps15, Mrps22, Mrps30, Mtx2, Ndufa8, Ndufb2, Ndufb6, Ndufb7, Ndufb8, Ndufc2, Ndufs1, Ndufs2, Ndufs3, Ndufs4, Ndufs6, Ndufs7, Ndufv1, Ndufv2, Oat, Opa1, Oxa1l, Pdha1, Pdhb, Pdhx, Pdk4, Pdp1, Phb2, Phyh, Pmpca, Por, Prdx3, Retsat, Rhot1, Rhot2, Sdha, Sdhb, Slc25a11, Slc25a12, Slc25a20, Slc25a3, Slc25a4, Slc25a5, Sucla2, Suclg1, Supv3l1, Tcirg1, Timm10, Timm13, Timm23, Timm50, Timm9, Toml1l, Tomm22, Uqcrc1, Uqcrc2, Uqcrfs1, Vdac1, Vdac2, Vdac3.

WNT/β-catenin signaling pathway (38 genes)

Adam17, Axin1, Axin2, Ccnd2, Ctnnb1, Cul1, Dkk1, Dkk4, Dll1, Dvl2, Frat1, Fzd1, Fzd8, Hdac11, Hdac2, Hdac5, Hey1, Hey2, Jag1, Jag2, Kat2a, Lef1, Maml1, Myc, Ncor2, Ncstn, Nkd1, Notch4, Numb, Psen2, Ptch1, Rbpj, Skp2, Tcf7, Trp53, Wnt1, Wnt5b, Wnt6.

Coagulation pathway (93 genes)

Adam9, Anxa1, Apoa1, Arf4, C1ra, C3, C8a, C8b, Capn2, Casp9, Cd9, Cfb, Cfi, Comp, Cpn1, Csrp1, Ctse, Ctsh, Ctsk, Ctso, Dab2, Dct, Dpp4, Dusp14, Dusp6, F11, F12, F13b, F2, F8, Fbn1, Fga, Fgg, Fn1, Furin, Gda, Gm20547, Gm37035, Gnb2, Gp1ba, Gp9, Gsn, Hmgcs2, Hpn, Htra1, Iscu, Itga2, Itih1, Klf7, Klkb1, Lamp2, Lefty2, Lgmn, Lrp1, Lta4h, Masp2, Mmp11, Mmp14, Mmp15, Mmp2, Mmp3, Mmp7, Mmp9, Msrb2, Mst1, Omp, Pecam1, Pef1, Pf4, Plek, Prep, Proc, Pros1, Prss23os, Pzp, Rabif, Rapgef3, Rgn, Serpinb2, Serpinc1, Serpine1, Serping1, Sh2b2, Sirt2, Sparc, Thbd, Thbs1, Timp1, Timp3, Tmprss6, Usp11, Vwf, Wdr1.

P53 pathway (159 genes)

Abat, Abcc5, Abhd4, Acvr1b, Ada, Aen, Alox8, Ankra2, Apaf1, Atf3, Atp5g1, AW551984, B430305J03Rik, Baiap2, Bak1, Bax, Bmp2, Btg1, Btg2, C430049E01Rik, Ccdc97, Ccnd2, Ccnd3, Ccng1, Ccnk, Ccp110, Cd81, Cd82, Cdh13, Cdk5r1, Cdkn1a, Cdkn2a, Cdkn2aip, Cebpa, Cgrrf1, Clca2, Coq8a, Csrnp2, Cyfip1, Dcxr, Ddb2, Ddit3, Ddit4, Dgka, Dnttip2, Dram1, Ei24, Epha2, Ephx1, Eps8l2, F2r, Fbxw7, Fdxr, Fgf13, Fos, Foxo3, Fuca1, Gadd45a, Gls2, Gm44367, Hdac3, Hexim1, Hint1, Hist1h1c, Hras, Hspa4l, Ier5, Ikbkap, Inhbb, Ip6k2, Irak1, Iscu, Itgb4, Jag2, Jun, Kif13b, Krt17, Lactb, Lif, Mapkapk3, Mdm2, Mknk2, Mt2, Mxd1, Mxd4, Ndrg1, Nhlh2, Ninj1, Nnat, Nol8, Nudt15, Osgin1, P3h2, Pcna, Pdgfa, Perp, Pgm2l1, Phlda3, Pidd1, Pitpnc1, Plk2, Plk3, Plxnb2, Pmm1, Ppm1d, Ppp1r15a, Prkab1, Prmt2, Ptpre, Pvt1, Rab40c, Rack1, Rad51c, Ralgds, Rchy1, Retsat, Rhbdf2, Rpl18, Rpl36, Rps12, Rrad, Rrp8, S100a10, S100a3, Sat1, Sec61a1, Sertad3, Sesn1, Slc19a2, Slc35d1, Slc3a2, Slc7a11, Sp1, St14, Steap3, Stom, Tap1, Tax1bp3, Tgfa, Tm7sf3, Tnni1, Tob1, Tprkb, Traf4, Trafd1, Trp53, Trp63, Tsc22d1, Tspyl2, Txnip, Upp1, Vamp8, Vdr, Wrap73, Wwp1, Zbtb16, Zfp36l1, Zfp862-ps, Zmat3.

G2/M checkpoint pathway (168 genes)

2700099C18Rik, Abl1, AC173210.4, Amd1, Arid4a, Atf5, Atrx, Aurka, Bard1, Bcl3, Brca2, Bub1, Bub3, Casp8ap2, Cbx1, Ccdc97, Ccna2, Ccnb2-ps, Ccnd1, Ccnt1, Cdc20, Cdc25a, Cdc25b, Cdc27, Cdc45, Cdc6, Cdc7, Cdk1, Cdk4, Cdkn1b, Cdkn2c, Cenpe, Cenpf, Chek1, Chmp1a, Cks1b, Ctcf, Cul1, Cul3, Cul4a, Cul5, Dbf4, Ddx39, Dkc1, Dr1, E2f1, E2f2, E2f4, Efna5, Ewsr1, Exo1, Ezh2, Fbxo5, Fcho2, Foxn3, G3bp1, Gm13992, Gm14820, Gm16133, Gm26726, Gm31749, Gm6204, Gm826, H2afv, Hif1a, Hira, Hist1h2al, Hist2h2bb, Hmgb3, Hmgn2, Hnrnpd, Hnrnpu, Hspa8, Hus1, Ilf3, Jpt1, Katna1, Kif15, Kif20b, Kif2c, Kif5b, Kmt5a, Knl1, Kpna2, Kpnb1, Lbr, Lig3, Lmnb1, Mad2l1, Map3k20, Mapk14, Marcks, Mcm2, Mcm3, Mcm5, Mcm6, Meis1, Meis2, Mir3535, Mki67, Mtf2, Mybl2, Myc, Nasp, Nolc1, Notch2, Nsd2, Numa1, Nup50, Nup98, Odf2, Orc5, Orc6, Pafah1b1, Pccb, Pds5b, Plk1, Plk4, Pml, Pole, Polq, Prc1, Prmt5, Prpf4b, Pttg1, Pura, Racgap1, Rad23b, Rad54l, Rbm14, Rpa2, Rps6ka5, Sap30, Sfpq, Slc12a2, Slc35f6, Slc38a1, Slc7a1, Slc7a5, Smad3, Smarcc1, Smc1a, Smc2, Smc4, Sqle, Srsf1, Srsf2, Stag1, Stil, Syncrip, Tfdp1, Tle3, Tmem129, Tmpo, Tnpo2, Top1, Top2a, Tpx2, Tra2b, Traip, Ttk, Ube2c, Ube2s, Uck2, Upf1, Wrn, Xpo1, Zfp771.

TGF-β signaling pathway (47 genes)

AC158606.2, Acvr1, Apc, Arid4b, Bcar3, Bmp2, Bmpr1a, Bmpr2, Ccdc97, Cdh1, Cdk9, Cdkn1c, Ctnnb1, D030025P21Rik, Dbt, E330011O21Rik, Eng, Fkbp1a, Furin, Gm37401, Hipk2, Id1, Id3, Lefty2, Msh3, Ncor2, Nog, Pmepa1, Ppm1a, Ppp1cc, Ppp1r15a, Serpine1, Ski, Skil, Slc20a1, Smad1, Smad3, Smad6, Smad7, Smurf1, Smurf2, Sptbn1, Tgfbr1, Tgif1, Thbs1, Trim33, Wwtr1.

IL-2 STAT5 signaling pathway (148 genes)

1500004A13Rik, Abcb1b, AC138307.1, Ager, Ahcy, Ahnak, Ahr, Airn, Alcam, Anxa4, Aplp1, Arhgap26, Arl4a, Batf, Batf3, Bcl2, Bcl2l1, Bhlhe40, Bmp2, Bmpr2, Capg, Capn3, Car2, Casp3, Ccnd2, Ccnd3, Cd44, Cd81, Cd83, Cdc6, Cdkn1c, Cish, Ckap4, Col6a1, Csf1, Cst7, Ctbp2, Ctsz, Cyfip1, Dcps, Dennd5a, Dhrs3, Ecm1, Emp1, Eno3, Enpp1, Eomes, Etfbkmt, Etv4, Fam126b, Flt3l, Furin, Gabarapl1, Galm, Gata1, Gbp3, Glipr2, Gm14612, Gm16033, Gm20708, Gm28592, Gm37039, Gpr65, Gpr83, Gpx4, Hipk2, Hk2, Hopx, Huwe1, Igf1r, Ikzf2, Ikzf4, Il18r1, Il1r2, Il2rb, Irf4, Irf6, Irf8, Itgae, Itgav, Jak1, Klf6, Lclat1, Lif, Lrig1, Lrrc8c, Ltb, Map3k8, Map6, Mapkapk2, Mxd1, Myc, Myo1c, Myo1e, Ncoa3, Ncs1, Ndrg1, Nfil3, Nfkbiz, Nop2, Nrp1, Nt5e, P2rx4, P4ha1, Pdcd2l, Phtf2, Plagl1, Plec, Pou2f1, Ptch1, Pth1r, Ptrh2, Pus1, Rabgap1l, Rhob, Rhoh, Rora, Rragd, Scn9a, Sell, Selp, Serpinb6a, Serpinc1, She, Slc1a5, Slc29a2, Slc2a3, Slc39a8, Smpdl3a, Snx9, Socs2, Spp1, Srgn, St3gal4, Swap70, Tiam1, Tlr7, Tmem30b, Tnfrsf1b, Tnfrsf21, Tnfrsf4, Tnfrsf8, Tnfsf11, Traf1, Ttc39b, Uck2, Umps, Xbp1.

IL-6/JAK/STAT3 signaling pathway (49 genes)

Acvr1b, Acvrl1, Bak1, Ccdc97, Cd36, Cd44, Cd9, Cntfr, Csf1, Csf2rb, Gm21970, Gm43919, Grb2, Hax1, Il17ra, Il17rb, Il18r1, Il1b, Il1r1, Il1r2, Il2rg, Il6st, Il7, Inhbe, Irf1, Irf9, Itga4, Jun, Lepr, Ltb, Map3k8, Myd88, Pdgfc, Pf4, Pik3r5, Ptpn1, Ptpn11, Ptpn2, Pzp, Socs3, Stam2, Stat1, Stat2, Stat3, Tlr2, Tnfrsf1a, Tnfrsf1b, Tnfrsf21, Tyk2.

Peroxisome pathway (79 genes)

Abcb1b, Abcc5, Abcc8, Abcd1, Abcd2, Abcd3, Acaa1a, Acot8, Acsl1, Acsl4, Acsl5, Aldh9a1, Asap2, Atxn1, Cacna1b, Cadm1, Cat, Catsper3, Cdk7, Cel, Cln6, Cnbp, Crat, Ctbp1, Ctps, Dhrs3, Dio1, Dlg4, Ech1, Ehhadh, Elovl5, Ephx2, Ercc1, Etfdh, Fdps, Fis1, Gm34086, Gm8566, Gnpat, Hao2, Hmgcl, Hras, Hsd11b2, Hsd17b4, Ide, Idh1, Idh2, Idi1, Lonp2, Mlycd, Msh2, Mvp, Nr1i2, Pex11a, Pex11b, Pex13, Pex14, Pex2, Pex5, Pex6, Prdx1, Prdx5, Rdh12, Retsat, Rxrg, Scp2-ps2, Sema3c, Slc23a2, Slc25a19, Slc25a4, Slc27a2, Smarcc1, Sod2, Syne2, Top2a, Ttr, Ugt2b34, Vps4b, Ywhah.

Allograft rejection pathway (119 genes)

Aars, Abce1, Abi1, AC158606.2, Ache, Acvr2a, Akt1, Apbb1, Bcl3, Brca1, Capg, Cartpt, Ccdc97, Ccl5, Ccnd2, Ccnd3, Ccr2, Ccr5, Cd247, Cd3e, Cd3g, Cd4, Cd47, Cd74, Cd79a, Csf1, Csk, Cxcr3, Dars, Degs1, Dyrk3, Egfr, Eif3d, Eif3j1, Eif4g3, Eif5a, Elf4, Ets1, F2, F2r, Fgr, Flna, Galnt1, Gbp2b, Gcnt1, Gm11787, Gm20547, Gm26666, Gpr65, Gzmb, H2-Aa, H2-DMa, H2-DMb2, H2-M5, H2-Q10, Hcls1, Hdac9, Hif1a, Igsf6, Ikbkb, Il15, Il16, Il18rap, Il1b, Il2rb, Il2rg, Il4, Il7, Inhba, Inhbb, Irf4, Irf7, Irf8, Itgal, Itgb2, Itk, Jak2, Lck, Lcp2, Lif, Ltb, Ly75, Map4k1, Mmp9, Mrpl3, Mtif2, Nlrp3, Nos2, Npm1, Pf4, Prkcb, Prkcg, Psmb10, Ptpn6, Ptprc, Rars, Rpl39, Rpl3l, Rps9, Slc39a11, Socs5, Spi1, St8sia4, Stab1, Stat1, Tap1, Tap2, Tcp11l1, Tgfb2, Timp1, Tlr1, Tlr2, Tlr3, Tpd52, Ube2d1, Ube2n, Wars, Was, Zap70.

Myogenesis pathway (163 genes)

Ablim1, Ache, Acsl1, Acta1, Actn3, Adcy9, Aebp1, Agl, Agrn, Akt2, Ankrd2, Aplnr, Apod, Atp2a1, Atp6ap1, Bag1, Bdkrb2, Bhlhe40, Bhlhe41, Bin1, Cacna1h, Camk2b, Casq1, Casq2, Ccdc97, Cd36, Cdh13, Cdkn1a, Cenpq, Chn1os3, Chrnb1, Chrng, Ckb, Ckm, Cnn3, Col15a1, Col1a1, Col3a1, Col4a2, Col6a2, Col6a3, Crat, Cryab, Csrp3, Des, Dmpk, Dtna, Efs, Eif4a2, Eno3, Ephb3, Erbb3, Fabp3, Fdps, Fgf2, Fhl1, Fkbp1b, Flii, Foxo4, Fxyd1, Gaa, Gm15589, Gm20708, Gnao1, Gpx3, Gsn, Hdac5, Hrc, Hspb2, Ifrd1, Igf1, Igfbp3, Igfbp7, Itga7, Itgb1, Itgb4, Kcnh1, Kifc3, Klhl7, Lama2, Large1, Ldb3, Lpin1, Lrfn4, Lsp1, Lypd5, Mapre3, Mef2a, Mef2c, Mef2d, Mras, Mybpc3, Mybph, Myh1, Myh2, Myh3, Myh4, Myh7, Myh8, Myh9, Myl1, Myl3, Myl4, Myl6b, Myl7, Mylpf, Myo1c, Myog, Myom1, Myom2, Myoz1, Nav2, Ncam1, Nos1, Nqo1, Oxtr, Palb2, Pde4dip, Pdlim7, Pgam2, Pick1, Pkia, Plxnb2, Ppfia4, Ppp1r3c, Psen2, Ptgis, Pvalb, Pygm, Reep1, Rit1, Ryr1, Scd1, Schip1, Sgca, Sgcd, Sgcg, Sh2b1, Sirt2, Slc6a8, Sln, Smtn, Sod3, Sparc, Spdef, Speg, Sptan1, St5, Stc2, Svil, Tagln, Tead4, Tnnc1, Tnnc2, Tnni1, Tnni2, Tnnt2, Tnnt3, Tpm2, Tpm3, Vipr1, Wwtr1, Zfp862-ps.

Adipogenesis pathway (148 genes)

Abca1, Abcb8, Acadm, Acads, Acly, Aco2, Adipor2, Agpat3, Aifm1, Ak2, Aldh2, Aldoa, Angptl4, Aplp2, Araf, Arl4a, Atl2, Atp1b3, Baz2a, Bckdha, Bcl2l13, Bcl6, Bhlhe41, C3, Carm1, Cat, Catsper3, Cavin1, Cavin2, Ccng2, Cd151, Cd36, Cdkn2c, Cfap126, Cfap46, Chchd10, Chuk, Cidea, Cmpk1, Col15a1, Coq3, Coq9, Cpt2, Crat, Cs, D17Wsu92e, Dgat1, Dhcr7, Dhrs7b, Dld, Dnajb9, Dram2, Ech1, Echs1, Elmod3, Enpp2, Ephx2, Esrra, Esyt1, Fzd4, G3bp2, Gadd45a, Gbe1, Ghitm, Gm11585, Gm29331, Gm35986, Gm43398, Gm44716, Gm45233, Gm4756, Gm8566, Gpat4, Gpd2, Gphn, Gpx3, Gpx4, Hadh, Idh1, Idh3a, Idh3g, Immt, Itga7, Itsn1, Lama4, Lep, Lipe, Lpcat3, Lpl, Map4k3, Mccc1, Mdh2, Me1, Mgll, Miga2, Mrpl15, Mtch2, Nabp1, Ndufb7, Ndufs3, Nmt1, Omd, Palb2, Pex14, Pfkfb3, Pfkl, Phldb1, Phyh, Pim3, Por, Pparg, Ppp1r15b, Prdx3, Preb, Ptcd3, Ptger3, Qdpr, Rab34, Reep6, Retsat, Rmdn3, Rpl15, Rreb1, Samm50, Scarb1, Scp2-ps2, Sdhb, Slc1a5, Slc25a1, Slc25a10, Slc27a1, Slc5a6, Sparcl1, Sqor, Stat5a, Stom, Suclg1, Taldo1, Tank, Tkt, Tob1, Ubc, Ubqln1, Uck1, Ucp2, Uqcrc1, Vegfb, Ywhag.

Spermatogenesis pathway (89 genes)

4930415F15Rik, AC153845.3, Ace, Acrbp, Adad1, Agfg1, Arl4a, Art3, Aurka, Braf, Bub1, Ccna1, Ccnb2-ps, Cct6b, Cdk1, Cftr, Chfr, Chrm4, Clgn, Clpb, Clvs1, Cnih2, Coil, Csnk2a2, Dbf4, Ddx25, Dmrt1, Dnajc1, Elovl3, Ezh2, Gad1, Gfi1, Gm16286, Gm8488, Gmcl1, Grm8, Gsg1, Gstm5, Hspa1l, Hspa2, Hspa4l, Ide, Ip6k1, Jam3, Kif2c, Lpin1, Mast2, Mfsd13b, Mlf1, Mtor, Ncaph, Nefh, Nf2, Nos1, Npy5r, Oaz3, Odf1, Pacrg, Pcsk1n, Pcsk4, Pebp1, Pgs1, Phkg2, Pomc, Prkar2a, Rad17, Rfc4, Scg3,

Scg5, Sept4, She, Sirt1, Slc12a2, Slc2a5, Snap91, Spata6, Stam2, Strbp, Sycp1, Taldo1, Tekt2, Tle4, Tnni3, Tsn, Ttk, Vdac3, Ybx2, Zc2hc1c, Zc3h14.

Epithelial mesenchymal transition pathway (153 genes)

6820431F20Rik, A930018P22Rik, Abi3bp, AC154816.1, AC161252.1, Anpep, Aplp1, Basp1, Bdnf, Bgn, Cadm1, Cald1, Cap2, Capg, Ccdc97, Cd44, Cdh6, Col11a1, Col12a1, Col16a1, Col1a1, Col1a2, Col3a1, Col4a2, Col5a3, Col6a2, Col6a3, Col7a1, Col8a2, Colgalt1, Comp, Crlf1, Cxcl12, Dab2, Dcn, Dkk1, Dpysl3, Dst, Ecm1, Ecm2, Efemp2, Eln, Eno2, Fap, Fbln2, Fbln5, Fbn1, Fermt2, Fgf2, Flna, Fmod, Fn1, Fstl1, Fstl3, Fuca1, Fzd8, Gadd45a, Gas1, Gem, Gja1, Gm10334, Gm16091, Gm16126, Gm38211, Gm43423, Gm44716, Gpc1, Gpx7, Grem1, Htra1, Igfbp2, Igfbp3, Igfbp4, Il15, Inhba, Itga2, Itgav, Itgb1, Jun, Lama1, Lama2, Lamc1, Lamc2, Lox, Loxl2, Lrp1, Magee1, Matn3, Mest, Mfap5, Mmp14, Mmp2, Mmp3, Msx1, Nid2, Notch2, Nt5e, P3h1, Pcolce, Pdgfrb, Pdlim4, Pfn2, Plod1, Plod2, Plod3, Pmepa1, Pmp22, Postn, Prrx1, Pthlh, Ptx3, Qsox1, Rgs4, Rhob, Sat1, Scg2, Sdc4, Serpine1, Serpine2, Serpinh1, Sfrp1, Sfrp4, Sgcb, Sgcd, Sgcg, Slc24a2, Slc6a8, Slit2, Slit3, Snai2, Sntb1, Sparc, Spock1, Spp1, Tagln, Tgfbi, Thbs1, Thbs2, Timp1, Timp3, Tnc, Tnfaip3, Tnfrsf11b, Tpm1, Tpm2, Vcam1, Vcan, Vegfa, Vegfc, Vim, Wipf1, Wnt5a, Zfp677.

PI3K/AKT/mTOR signaling pathway (89 genes)

AC158606.2, AC160995.1, Acaca, Actr2, Actr3, Akt1, Akt1s1, Ap2m1, Arf1, Arhgdia, Arpc3, Atf1-ps, Atp6v1e1, C430049E01Rik, Cab39, Cab39l, Calr, Cast, Cdk1, Cdk2, Cdk4, Cdkn1a, Cdkn1b, Cltc, Csnk2b, Cxcr4, Dapp1, Dbt, Ddit3, Dusp3, E2f1, Egfr, Eif4e, Fgf6, Gna14, Grb2, Grk3, Gsk3b, Hras, Hsp90b1, Il2rg, Il4, Itpr2, Lck, Map2k3, Map2k6, Mapk8, Mapk9, Mknk1, Mknk2, Myd88, Nfkbib, Nod1, Pak4, Pdk1, Pfn1, Pik3r3, Pikfyve, Pin1, Pitx2, Plcb1, Plcg1, Ppp1cc, Ppp2r1b, Prkaa2, Prkar2a, Prkcb, Pten, Ptpn11, Raf1, Ripk1, Rit1, Rps12-ps9, Rps6ka1, Rps6ka3, Rptor, Sla, Slc2a1, Slco1a6, Smad2, Sqstm1, Stat2, Tbk1, Tcp11l1, Tiam1, Tnfrsf1a, Ube2n, Vav3, Ywhab.

Protein secretion pathway (85 genes)

Abca1, AC137843.1, AC140409.5, Adam10, Airn, Anp32e, Ap1g1, Ap2b1, Ap2m1, Ap2s1, Arcn1, Arf1, Arfgap3, Arfgef1, Arfgef2, Atp1a1, Atp6v1b1, Atp6v1h, Atp7a, Bet1, Bhlhe41, Bnip3, Cav2, Cd63-ps, Clcn3, Cln5, Clta, Cltc, Cog2, Copb1, Copb2, Cope, Ctsc, Dst, Egfr, Ergic3, Galc, Gbf1, Gm5865, Gm6969, Gm8566, Gnas, Golga4, Gosr2, Ica1, Kif1b, Krt18, Lamp2, Lman1, M6pr, Mcph1, Mon2, Napa, Ocrl, Pam, Ppt1, Rab14, Rab22a, Rab9, Rer1, Rps6ka3, Scamp3, Scrn1, Sec22b, Sec24d, Sec31a, Snap23, Stam, Stx12, Stx16, Stx7, Tmed2, Tmx1, Tom1l1, Tpd52, Tsg101, Tspan8, Uso1, Vamp3, Vamp4, Vps45, Vps4b, Yipf6, Ykt6, Zfp367.
